# TPX2-mediated autophagy maintains cancer stemness in LUAD: bioinformatic screening and functional validation

**DOI:** 10.3389/fonc.2026.1724797

**Published:** 2026-06-02

**Authors:** Mingzheng Jiang, Hongli Ye, Jiwei Li, Jing Wu, Xiaoyi Hu, Qiuyue Long, Liang Bu, Zhancheng Gao, Yali Zheng

**Affiliations:** 1 Department of Respiratory, Critical Care and Sleep Medicine, Xiang’ an Hospital of Xiamen University, School of Medicine, Xiamen University, Xiamen, China; 2 Institute of Chest and Lung Diseases, Xiamen University, Xiamen, China; 3 Department of Thoracic Surgery and Oncology, the First Affiliated Hospital of Guangzhou Medical University, State Key Laboratory of Respiratory Disease & National Clinical Research Center for Respiratory Disease, Guangzhou, China; 4 Department of Respiratory and Critical Care Medicine, Peking University People’s Hospital, Beijing, China; 5 Department of Thoracic Surgery, Xiang an Hospital of Xiamen University, School of Medicine, Xiamen University, Xiamen, China

**Keywords:** autophagy, lung adenocarcinoma, therapeutic target, TPX2, tumor stemness

## Abstract

Targeting protein for Xklp2 (TPX2) is a known mitotic regulator overexpressed in lung adenocarcinoma (LUAD), yet its role in cancer stemness and autophagy remains unclear. Through integrative bioinformatic analysis of TCGA and GTEx datasets, we discovered that TPX2 overexpression in LUAD correlates not only with poorer overall, disease-free, disease-specific, and progression-free survival but also significantly associates with elevated stemness scores (RNAss/DNAss) and increased expression of CSC markers SOX2 and c−MYC. Functionally, CRISPR-Cas9-mediated TPX2 knockout in PC9 and H1975 cells led to marked reductions in sphere-forming capability and CSC marker expression, while TPX2 overexpression had the opposite effect. Mechanistic exploration through Co-immunoprecipitation coupled with mass spectrometry (IP-MS) and transcriptome analysis revealed a correlation between TPX2 and autophagy regulation in LUAD cells. TPX2 knockdown inhibited autophagic flux, as evidenced by decreased Beclin−1 and accumulation of p62 and LC3-II levels, together with increased GFP+/mRFP+ (yellow) LC3 puncta. Conversely, TPX2 overexpression enhanced autolysosome formation. Importantly, treating TPX2-overexpressing cells with the autophagy inhibitor chloroquine significantly reversed their enhanced migration, proliferation, and stemness marker expression. *In vivo*, TPX2 silencing in xenograft models reduced tumor growth and altered autophagy marker profiles. These findings unveil a novel mechanism whereby TPX2 promotes CSC properties in LUAD by driving autophagy, offering a promising therapeutic avenue targeting TPX2-mediated autophagy.

## 1 Introduction

Targeting protein for Xenopus kinesin-like protein 2 (TPX2) is a microtubule-associated protein essential for mitotic spindle assembly, primarily through the activation of the cell cycle kinase protein Aurora A, which plays a key role in chromosomal instability and tumorigenesis (1, 2).TPX2 upregulation has been reported in various cancers, including colon, renal cell carcinoma, pancreatic, breast, and hepatocellular carcinoma, and is frequently associated with poor prognosis (3–7). Mechanistically, elevated TPX2 expression can induce centrosome amplification and DNA polyploidy, leading to uncontrolled cell proliferation, impaired apoptosis, and enhanced tumor progression (8–10).

In lung adenocarcinoma (LUAD), the predominant subtype of non-small cell lung cancer (NSCLC), TPX2 overexpression has been linked to enhanced metastasis and malignant progression. Functional studies indicate that TPX2 knockdown reduces LUAD cell proliferation and migration, suggesting its potential role in tumor aggressiveness (8, 9, 11). In recent years, targeted therapy and immunotherapy have significantly improved the survival rate of advanced NSCLC patients, with a 5-year overall survival (OS) rate reaching 20% to 30% (12, 13). Therefore, understanding the mechanisms by which TPX2 contributes to LUAD progression is crucial for developing novel therapeutic strategies.

Autophagy, a highly conserved cellular process responsible for degrading and recycling intracellular components, plays a dual role in cancer. In the early tumorigenesis, autophagy functions as a tumor suppressor by maintaining the integrity of the cellular environment and preventing the accumulation of damaged organelles and proteins. However, in advanced cancers, autophagy facilitates tumor survival by providing metabolic substrates under stress conditions, such as hypoxia and nutrient deprivation (14).

Emerging evidence suggests that autophagy is critical for the maintenance of cancer stem cells (CSCs) (15), a subpopulation of tumor cells responsible for tumor initiation, metastasis, and therapy resistance (16). By degrading damaged cellular components, autophagy prevents CSC senescence and sustains their self-renewal capacity, thereby contributing to tumor persistence and recurrence (17). Consequently, targeting autophagy represents a promising strategy to eradicate CSCs and improve therapeutic efficacy in LUAD. However, whether TPX2 regulates autophagy and its impact on CSC maintenance in LUAD remains unclear, warranting further investigation.

In this study, we investigated the role of TPX2 in regulating autophagy and cancer stemness in LUAD. We identified TPX2 as a potential prognostic biomarker for LUAD progression using public datasets and clinical samples. Functional assays demonstrated that TPX2 overexpression enhances autophagic flux and promotes the expression of stemness-related genes, while inhibition of autophagy using chloroquine attenuates TPX2-mediated oncogenic effects. These findings suggest that TPX2 promotes stem-like properties in LUAD cells through the activation of autophagy, highlighting its potential as a therapeutic target in LUAD treatment.

## 2 Materials and methods

### 2.1 TCGA pan-cancer atlas data download and analysis

RNA-sequencing data for pan-cancer samples was obtained from The Cancer Genome Atlas (TCGA) and Genotype-Tissue Expression (GTEx) databases via UCSC Xena (https://xenabrowser.net/). The “Gene DE” module of tumor immune estimation resource, version 2 (TIMER2) web server (https://cistrome.shinyapps.io/timer/) was explored with the input of “TPX2” to explore the expression differences of TPX2 gene between tumor and normal tissues in 33 cancer types (ACC, BLCA, BRCA, CESC, CHOL, COAD, DLBC, ESCA, GBM, HNSC, KICH, KIRC, KIRP, LAML, LGG, LIHC, LUAD, LUSC, MESO, OV, PAAD, PCPG, PRAD, READ, SARC, SKCM, STAD, TGCT, THCA, THYM, UCEC, UCS, UVM). Normalized transcriptomic expression values (log2[transcripts per million (TPM) + 1]) were used for differential expression analysis. For cancer types with limited normal tissue samples in TCGA (including DLBC, GBM, PRAD, TGCT, THYM), we incorporated GTEx normal tissue data using the GEPIA2 web tool (http://gepia2.cancer-pku.cn/) (18), under the settings of p-Value cutoff = 0.01, log2 fold change (FC) cutoff = 1, and “Match TCGA normal and GTEx data” were set as criteria. Additionally, violin plots of the TPX2 expression in different pathological stages of TCGA tumors through the “pathological stage plot” module of GEPIA2 were obtained. The log2 (transcripts per million (TPM) + 1) transformed expression data were applied for the violin plots.

### 2.2 Survival and prognostic analysis

To analyze the DFI (Disease Free Interval), DSS (Disease Specific Survival), OS (Overall Survival), and PFI (Progression Free Interval) of patients with lung adenocarcinoma, 517 patient samples from TCGA were analyzed by PanCanSurvPlot (https://smuonco.shinyapps.io/PanCanSurvPlot/) (19).

### 2.3 Tumor stemness score analysis

We downloaded a standardzed pan-cancer dataset: TCGA Pan-Cancer (PANCAN, N = 10535, G = 60499) from the UCSC database. We further extracted TPX2 gene expression data from each sample. Then, we obtained the RNA stemness score (RNAss) and DNA stemness score (DNAss) for each tumor from a previous published dataset (20). After integrating the sample stemness index with gene expression data, further transformed each gene value using log2 (X + 0.001) through the “limma” R package, and finally obtained the expression data of 33 cancer types. To assess the relationship between TPX2 and stemness scores, we conducted Spearman’s correlation analysis and visualized the results using the “corrplot” R package. Additionally, we utilized the “ggplot2” package in R to compute the Spearman correlation between TPX2 and key cancer stem cell markers, including OCT-4 [POU5F1], TCF4, ALDH1A1, C-Myc [MYC], β-catenin [CTNNB1], cyclin D1 [CCND1], SOX2, NANOG and CD133 [PROM1] (21, 22). All gene symbols were standardized according to the HUGO Gene Nomenclature Committee (HGNC) nomenclature.

### 2.4 CRISPR-Cas9 gene knock-out

The human lung adenocarcinoma cell lines H1975 and PC9 were purchased from Procell (Wuhan, China). To generate TPX2-knockout (sgTPX2) LUAD cell lines, we employed the CRISPR-Cas9 system. First, 293T cells were transfected with the pLV-CAS9-Blast plasmid along with the lentiviral packaging plasmids psPAX2 and pMD2.G for lentivirus production (all the plasmids were purchased from the Addgene). The resulting lentiviral supernatant was harvested and used to infect NCI-H1975 and PC9 cells, followed by selection with blasticidin to establish stable Cas9-expressing cell lines. Next, two single-guide RNA (sgRNA) sequences targeting exon 5 and exon 6 of the TPX2 gene were designed and cloned into sgRNA expression plasmids (sgRNA1 (exon 5): 5’-CCAATTTGGAGAATAAGTT-3’; sgRNA2 (exon 6): 5’-GCAGAAAAAGAAAATCTTGT-3’). A non-targeting sgRNA was used as a negative control (sgNC). Lentiviruses carrying sgTPX2 or sgNC constructs were produced and used to infect Cas9-expressing LUAD cells. Successful TPX2 knockout was confirmed by RT-PCR and Western blot.

### 2.5 Establishment of stable TPX2 partial depletion and overexpressing cell line

DNA fragments targeting TPX2 were designed and synthesized (**Supplementary Table 1**), and then cloned into the AgeI/EcoRI sites of the pLKO.1 RNAi vector to generate lentiviral plasmids for partial depletion of TPX2 (shTPX2) and a negative control (TPX2-shNC)(puromycin resistance). The TPX2 coding sequence was cloned into the lentiviral expression vector (pLenti-CMV-TPX2-3FLAG-Blast), and the corresponding empty vector (pLenti-CMV-Empty-Blast) served as the negative control (TPX2-NC). Lentiviral particles were generated by co-transfecting the expression or RNAi plasmids with the packaging vectors psPAX2 and pMD2.G into HEK293T cells using Lipofectamine™ 3000 (Invitrogen). After 48 h, the viral supernatant was collected, filtered, and used to infect H1975 and PC9 cells in the presence of polybrene (8 μg/mL). Infected cells were cultured for an additional 48 h and then subjected to puromycin or blasticidin selection to establish stable TPX2-partial depletion and overexpressing cell lines. The efficiency of TPX2 overexpression was confirmed in the selected cells by qPCR and Western blotting.

### 2.6 Sample preparation for immunoprecipitation-mass spectrometry

H1975 cells expressing TPX2-Flag or the corresponding non-expressing control cells were washed with cold PBS and lysed in RIPA buffer (Beyotime Biotechnology, P0013C) supplemented with PMSF (Thermo Scientific) and protease inhibitors (Roche, 04693132001) for 10min on ice. Lysates were sonicated for 30s and incubated on ice for 1h, followed by centrifugation to collect the supernatant. For immunoprecipitation, cell lysates were incubated with anti-Flag antibody (66008-4-Ig, Proteintech) at 4°C with gentle rotation, followed by incubation with 50 µl protein A/G magnetic beads (MedChemExpress, cat# HY-K0202) for 3h at 4°C. Beads were washed extensively, and bound proteins were subjected to downstream mass spectrometry analysis. TPX2-Flag-non-expressing cells were processed in parallel and used as a background control to identify nonspecific binders.

Mass spectrometry was performed on an Orbitrap Fusion Lumos Tribrid mass spectrometer (Thermo Scientific) operated in data-dependent acquisition (DDA) mode. Proteomic data were analyzed using Proteome Discoverer software (v2.4, Thermo Fisher Scientific). Tandem mass spectra were searched against the UniProt human protein database using the SEQUEST HT and Mascot search engines. Peptide and protein identifications were filtered using a false discovery rate (FDR) threshold of ≤1% based on the Percolator validation algorithm.

A total of 4,137 proteins were identified in the TPX2-associated IP-MS dataset. To reduce background and focus on biologically relevant candidates, proteins were filtered based on their preferential presence in TPX2-Flag immunoprecipitates compared with the non-expressing control samples. From this dataset, autophagy-related candidates were identified by intersecting the 4,137 proteins with a curated autophagy-related gene set obtained from the Human Autophagy Database (http://autophagy.lu), yielding a subset of 337 proteins. These 337 autophagy-related proteins were subsequently subjected to KEGG pathway enrichment analysis using the clusterProfiler package in R (v4.3.1). Pathway enrichment significance was assessed using adjusted *p* values based on the Benjamini–Hochberg correction. The corresponding protein lists are provided in **Supplementary Table 2**.

### 2.7 Identification of DEGs and KEGG enrichment analysis

Total RNA was isolated from samples using the RNA Extraction Kit (AG, 21017). RNA integrity was verified using an Agilent 2100 Bioanalyzer (RNA Integrity Number ≥ 7.0), with concentration determined by Qubit fluorometric quantification. Poly(A) and RNA was enriched using oligo (dT) magnetic beads prior to library preparation.

RNA sequencing libraries were constructed using the BGI RNA Library Prep Kit following manufacturer’s protocols. High-throughput sequencing was performed on DNBSEQ-T7/G50 platforms using a paired-end 150 bp (PE150) strategy. Raw sequencing data underwent quality control processing using SOAPnuke (BGI) to remove low-quality reads and adaptor sequences. Clean reads were aligned to the reference genome using HISAT2/Bowtie2, with gene expression quantification performed using RSEM or StringTie algorithms.

The autophagy-related genes (AGs) were collected from the Human Autophagy Database (http://autophagy.lu) and used for annotations in the volcano plot. The differential expression analysis was conducted using the limma package in R (version 4.3.1). The raw read counts from PC9 cells were normalized using the voom transformation. Differentially expressed genes were identified based on the following criteria: p-value ≤ 0.1 and log (Foldchange) ≥ 0.3. The differentially expressed genes were subjected to KEGG pathway enrichment analysis using the clusterProfiler package. The results were visualized with the ggplot2 package, focusing on identifying significantly enriched pathways. The transcriptomics data have been included in the **Supplementary Table 3**.

### 2.8 Tumor spheroid formation assay

To evaluate the self-renewal ability of LUAD cells, a tumor spheroid formation assay was performed. Cells were suspended in serum-free culture medium supplemented with 1% penicillin-streptomycin, 20 ng/ml epidermal growth factor, 20 ng/mL basic fibroblast growth factor, and 2% B27. Single-cell suspensions were seeded at a density of 1000 cells/well in 24-well ultra-low attachment plates. After 7 days of culture, the number of tumor spheres with a diameter >50 μm was counted under an inverted microscope (MF52-N, Mshot).

### 2.9 Single-cell cloning assay

For single-cell colony formation analysis, cells were enzymatically dissociated into a single-cell suspension and seeded into 6-well plates at a density of 1000 cells/well. Cells were cultured at 37 °C with 5% CO_2_ for 1–2 weeks until colonies were visible. The resulting colonies were fixed with 4% paraformaldehyde and incubated at 4°C for 30min, then stained with 0.1% crystal violet (Beyotime, China) for 40 min at room temperature. After staining, plates were washed, and excess liquid was removed using filter paper before imaging and quantification.

### 2.10 Cell invasion assay

The Matrigel invasion assay was conducted using Matrigel-coated Transwell chambers (354480, Corning, USA). A total of 2 × 10^4^ cells in serum-free medium were seeded into the upper chamber, while 600 μL of complete medium containing 20% FBS was added to the lower chamber as a chemoattractant. In the treatment group, cells were treated with chloroquine (CQ, 10μM) for 24h before analysis. After incubation, non-invading cells were removed from the upper chamber, and invaded cells were fixed with 4% paraformaldehyde (Meilunbio, China) and stained with 0.1% crystal violet (Beyotime, China) for 40min at room temperature. Invaded cells were visualized and counted under a microscope (MF52-N, Mshot).

### 2.11 Wound healing assay

The wound healing assay was used to assess cell migration capacity. PC9 and H1975 cells transfected with the indicated plasmids were seeded into 6-well plates and cultured until the cells reached 90% confluence. A sterile 200μL pipette tip was used to create a scratch wound across the monolayer. The cells were then incubated in culture medium (containing 10% FBS and 1%PS) or medium supplemented with CQ (10 μM). Since wound healing in 10% FBS may allow limited cell proliferation, Transwell migration assays were performed in parallel to exclude proliferation-related effects. Images were taken at 0, 12, and 24 hours using an inverted microscope and the wound closure rate was analyzed using ImageJ (V1.8.0.112), based on the relative blank area at each time point.

### 2.12 Reverse transcription and quantitative real-time PCR

Total RNA was extracted using the SteadyPure Universal RNA Extraction Kit (Accurate biology, China) following the manufacturer’s instructions. Complementary DNA (cDNA) was synthesized using Evo M-MLV RT Premix (Accurate biology, China). Quantitative PCR (qRT-PCR) was conducted using Hieff ^®^ qPCR SYBR Green Master Mix (Yeasen, China) on a real-time PCR system (BIO RAD, USA). Gene expression levels were calculated using the 2^−^ΔΔCt method, with β-actin as an internal control. Primers used in this study are listed in **Supplementary Table 4**.

### 2.13 Western blot analysis

LUAD cells were lysed in radioimmunoprecipitation assay (RIPA) buffer (Beyotime) supplemented with PMSF and a protease inhibitor cocktail (PIC). Protein concentrations were measured using the bicinchoninic acid assay according to the manufacturer’s instructions (23225, Thermo Fisher Scientific). Equal amounts of protein (25μg per sample) were separated by 8 - 12.5% sodium dodecyl sulfate–polyacrylamide gel electrophoresis (SDS-PAGE) and transferred onto a polyvinylidene difluoride (PVDF) membrane (Bio-Rad, USA). The membranes were blocked in 5% nonfat milk (BD, USA) and incubated with the primary antibodies at 4 °C overnight. Finally, the membranes were incubated with horseradish peroxidase (HRP)-conjugated secondary antibodies and visualized using an Azure C300 (Azure Biosystems, USA) imaging system. The following primary antibodies were used: LC3B (ab192890, Abcam), Beclin1 (ab207612, Abcam), SOX2 (3579T, Cell Signaling Technology), p62 (5114S, Cell Signaling Technology), c-MYC (10828-1-AP, Proteintech), and β-Actin (66009-1-Ig, Proteintech). The secondary antibody (anti-Rabbit, SA00001-2) was purchased from Proteintech. The grayscale intensity ratios of Western blot bands were quantified using ImageJ software (1.54p), and the resulting data were statistically analyzed in GraphPad Prism 8.0.1.

### 2.14 Transmission electron microscopy

H1975 and PC9 cells were treated with or without LC3 for 24 h and then collected after trypsin digestion. The cells were fixed overnight with 2.5% glutaraldehyde at 4°C, post-fixed with 1% osmium tetroxide (OsO_4_) for 2 h, dehydrated through graded ethanol (70%, 80%, 90% and 95%) and acetone, embedded overnight in liquid epoxy resin, made ultra-thin sections and stained with uranyl acetate and lead citrate. The autophagosomes in H1975 and PC9 cells were observed under transmission electron microscope.

### 2.15 GFP-mRFP-LC3 puncta assay

For autophagic flux analysis, LUAD cells were seeded onto sterile coverslips in 24-well plates at a density allowing 60–80% confluence before transfection. Cells were then transduced with GFP-mRFP-LC3 adenovirus (HanBio Technology, Shanghai, China) for 24 h. After washing with PBS, live cells were maintained in phenol-red-free culture medium and imaged directly without fixation or permeabilization. GFP and mRFP signals were acquired using a Zeiss confocal microscope and merged for quantification. Because GFP fluorescence is selectively quenched upon fusion with acidic lysosomes, whereas mRFP remains stable, GFP+/mRFP+ (yellow) puncta identify autophagosomes, while RFP-only (red) puncta mark autolysosomes, thereby enabling visualization of autophagic flux.

### 2.16 Subcutaneous tumor transplantation assay in BALB/c nude mice

All animal experiments were conducted in compliance with the guidelines for the Care and Use of Laboratory Animals of the National Institutes of Health, and were approved by the Animal Ethics Committee of Xiamen University (approval no. XMULAC20250016). The sgNC or sgTPX2 cells (5×10^6^ cells per mouse) were subcutaneously injected into the right armpit of 8-week-old BALB/c nude mice. Tumor size was measured every 4 days and tumor volume was calculated using the formula: V = (length×width^2^)/2. Mice were euthanized when tumor volume reached 1000 mm^3^ (Mice were euthanized by overdose inhalation of isoflurane. Briefly, animals were placed in an induction chamber and exposed to 3–4% isoflurane for anesthesia, followed by continuous exposure to 5% isoflurane until respiratory and cardiac arrest occurred. Death was confirmed by the absence of respiration and heartbeat, as well as the loss of corneal and pedal reflexes). Tumor specimens were collected for further analysis. Tumor sizes and masses were measured and then fixed in 4% paraformaldehyde for hematoxylin-eosin (H&E) and immunohistochemical (IHC) analysis. All procedures were performed under the ethical guidelines outlined in the Declaration of Helsinki.

### 2.17 H&E and IHC staining

For H&E staining, tissues were fixed in 4% paraformaldehyde for 24 hours and subjected to gradient ethanol dehydration and paraffin embedding. Paraffin-embedded tissues were sectioned into 4-μm-thick slices, deparaffinized, and subjected to H&E staining before mounting.

For IHC staining, paraffin-embedded tumor sections were incubated at 37 °C overnight, deparaffinized, and treated with 3% hydrogen peroxide for 15 minutes to quench endogenous peroxidase activity. Antigen retrieval was performed by microwave heating, followed by natural cooling for 40 minutes. Tissue sections were incubated with primary antibodies against TPX2, p62, LC3B, Beclin1, followed by biotin-labeled secondary antibodies. Immunoreactivity was visualized using the Leica Aperio Versa 200 digital pathology scanner (Leica Biosystems, Germany), and images were acquired with Aperio Image Scope 12.3 software.

### 2.18 Statistical analysis

All statistical analyses were conducted using GraphPad Prism 9.5 (GraphPad Software, USA). Normality was assessed using the Shapiro–Wilk test. Parametric tests (Student’s *t*-test or one-way ANOVA with Tukey’ *post hoc* test) were used for normally distributed data and presented as mean ± SD. Non-parametric tests (Mann-Whitney U, Wilcoxon signed-rank, or Kruskal-Wallis test) were applied to non-normal data, which are reported as median (IQR). Statistical significance was set at *p < 0.05.

## 3 Results

### 3.1 TPX2 is highly expressed in LUAD and correlates with prognosis

**Figure 1:**
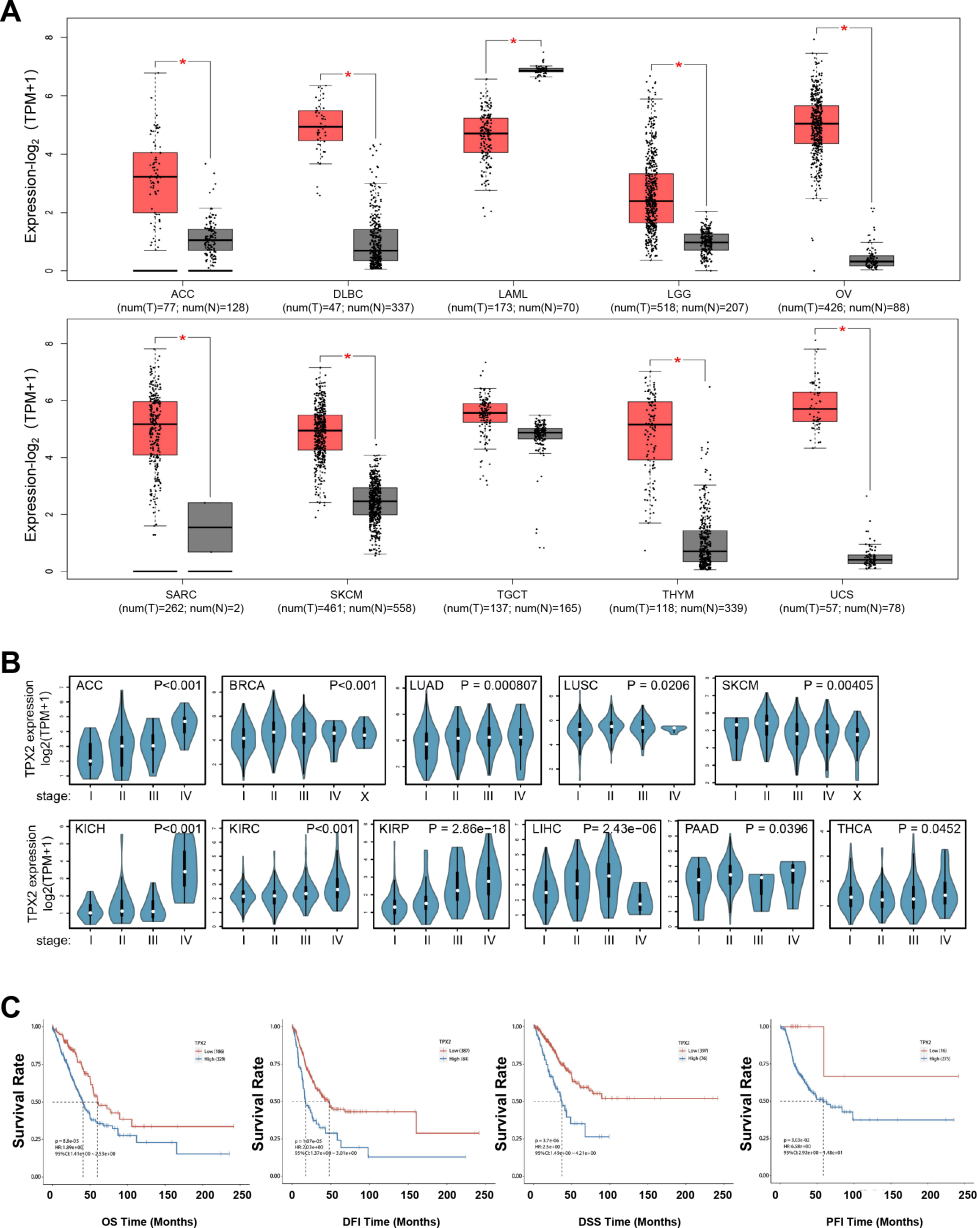
Comprehensive analysis of TPX2 gene expression across diverse tumor types and pathological stages. **(A)** Comparative analysis of TPX2 gene expression levels in the remaining cancer data obtained from the Genotype-Tissue Expression (GTEx) project. **(B)** TPX2 transcription levels across pathological stages (stages I, II, III, and IV) in various cancers. *p < 0.05, **p < 0.01, ***p < 0.001. Data were presented as median and interquartile range (IQR). **(C)** The correlation between TPX2 expression levels and clinical outcomes of LUAD patients, including OS (Overall Survival), DFI (Disease Free Interval), DSS (Disease Specific Survival) and PFI (Progression Free Interval). The area between the upper and lower blue/red dashed lines indicated the 95% confidence interval (CI) for the survival estimates.

Previous studies have shown that TPX2 is highly expressed in 21 out of 33 cancer types from the TCGA dataset when compared to adjacent normal tissues, including LUAD and LUSC (23, 24). For the remaining 12 cancer types which normal tissue data was unavailable, we further assessed TPX2 mRNA expression using GEPIA2, which integrates data from both TCGA and GTEx. Excluding two tumor types (MESO, UVM) where normal tissue data was still lacking, we explored TPX2 expression in the remaining 10 cancers. Our findings showed that TPX2 expression was upregulated in tumor tissues of 8 out of the 10 cancer types (*p < 0.05), downregulated in LAML, and showed no significant difference in TGCT (**Figure 1A**). In addition, we explored the correlation of TPX2 expression and the pathological stages of all 24 available cancers using GEPIA2. A significant positive correlation was found in 11 out of the 24 cancers, including both LUAD and LUSC (**Figure 1B**). while no significant differences were observed in the remaining 13 cancers (**Supplementary Figure 1A**).

Since NSCLC has now accounting for the most significant numbers of cancer-related deaths worldwide, and LUAD is justified by its prevalence as the most common subtype of NSCLC, we further explored the correlations between TPX2 expression levels and clinical outcomes in LUAD cases. Survival analysis by Kaplan-Meier plotter showed high TPX2 expression was associated with poor OS, DFI, DSS, and PFI in LUAD patients (**Figure 1C**, and a summary of the clinical characteristics of the enrolled LUAD patients is provided in **Supplementary Table 5**). Collectively, these results indicated that TPX2 expression at the mRNA levels was upregulated in LUAD, which suggested that TPX2 expression could serve as a potential biomarker for the prevention of LUAD progression and deterioration.

### 3.2 TPX2 gene expression is positively associated with tumors stemness in LUAD

**Figure 2:**
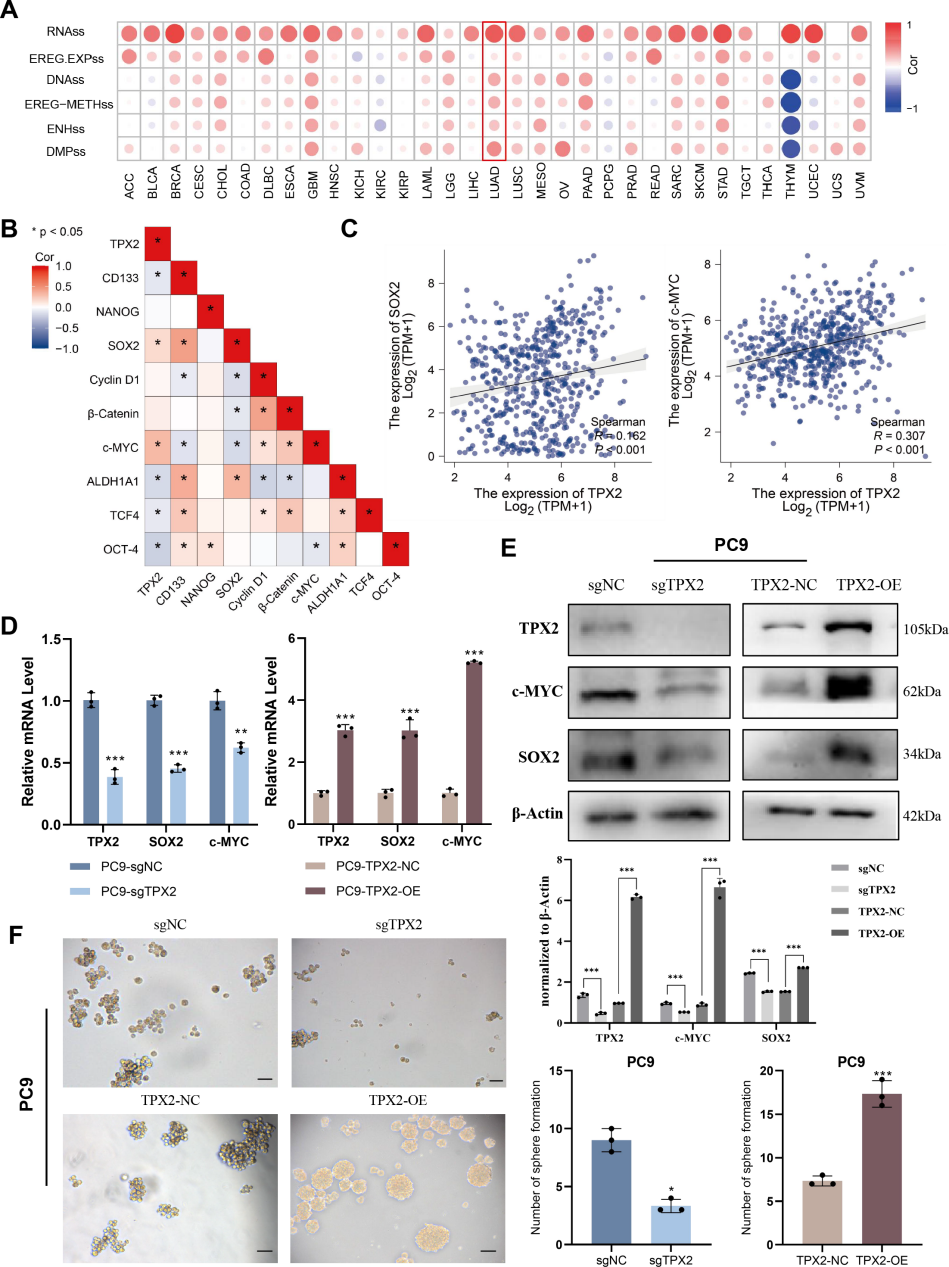
Exploring the correlation between TPX2 expression and tumor stemness. **(A)** Correlation analysis was conducted to assess the relationship between TPX2 and various stemness scores, including RNAss (RNA expression-based score), EREG.EXPss (Epigenetically regulated RNA expression-based), DNAss (DNA methylation based Stemness Scores), EREG-METHss (Epigenetically regulated DNA methylation-based), ENHss (Enhancer Elements/DNA amplification based) and DMPss (differentially methylated probes based). Red dots indicated a positive relationship between TPX2 expression and tumor stemness. **(B)** Heatmap depicting the correlation between TPX2 and genes associated with stemness. **(C)** Correlation analysis of TPX2 mRNA expression levels with stemness markers SOX2 and c-MYC. **(D)** The mRNA and **(E)** protein expression levels of SOX2, and c-MYC in PC9 cells post TPX2 knockout (sgTPX2) or overexpression (TPX2-OE). The figure below shows the β-Actin-normalized gray values of TPX2, c-MYC, and SOX2 in the corresponding Western blot bands. **(F)** Comparative analysis of sphere formation in PC9 cells with sgTPX2, TPX2-OE, and their respective controls (sgNC and TPX2-NC). Scale bars: 50 μm. *p < 0.05, **p < 0.01, ***p < 0.001.

Cancer stem cells (CSCs), known for their role in tumor initiation and progression, contribute significantly to tumor proliferation, migration, metastasis, epithelial-mesenchymal recurrence, and therapeutic drug resistance (20, 25). Given the established link between TPX2 overexpression and poor prognosis in various cancers, we hypothesize its potential role in modulating the stem-like properties of cancer cells, which may underlie its impact on cancer progression and therapeutic resistance. Therefore, we investigated the relationship between TPX2 gene expression and the stemness of tumors. Our analysis revealed a significant positive correlation between TPX2 expression and stemness scores, both at the DNA (DNAss) and RNA (RNAss) levels, across a range of cancer types (**Figure 2A**). As expected, TPX2 was positively associated both with RNAss and DNAss in LUAD patients. This association was further supported by the positive correlation observed between TPX2 and the expression of established CSC markers, including SOX2 and c-MYC (**Figures 2B, C**).

To experimentally validate this hypothesis, the expression levels of TPX2 protein were first assessed in four LUAD cell lines (H1975, PC9, A549, and HCC827), and compared to those in untransformed human bronchial epithelial cells (BEAS-2B). The results revealed that TPX2 expression was consistently higher in all four LUAD cell lines than in BEAS-2B cells. Among these, H1975 and PC9 exhibited relatively high endogenous levels of TPX2, and were thus selected as the primary cell models for subsequent analysis (**Supplementary Figure 2A**). TPX2 expression was subsequently manipulated in the LUAD cell lines (PC9, H1975), and its impact on CSC markers was assessed. TPX2 knockout (sgTPX2) led to a significant downregulation of SOX2 and c-MYC at both the transcriptional and protein levels, compared to control cells. Conversely, TPX2 overexpression resulted in an upregulation of these markers (**Figures 2D, E** and **Supplementary Figure S2B, C**, ***p < 0.001). Moreover, the ability of LUAD cells to form spheres, a hallmark of stemness, was significantly impaired in the absence of TPX2, while TPX2 overexpression promoted this phenotype (**Figure 2F** and **Supplementary Figure S2D**, ***p < 0.001). These results indicated that TPX2 could positively regulate stemness in LUAD cells, potentially contributing to the aggressive behavior of these tumors.

### 3.3 TPX2 induces autophagy in LUAD cells

**Figure 3:**
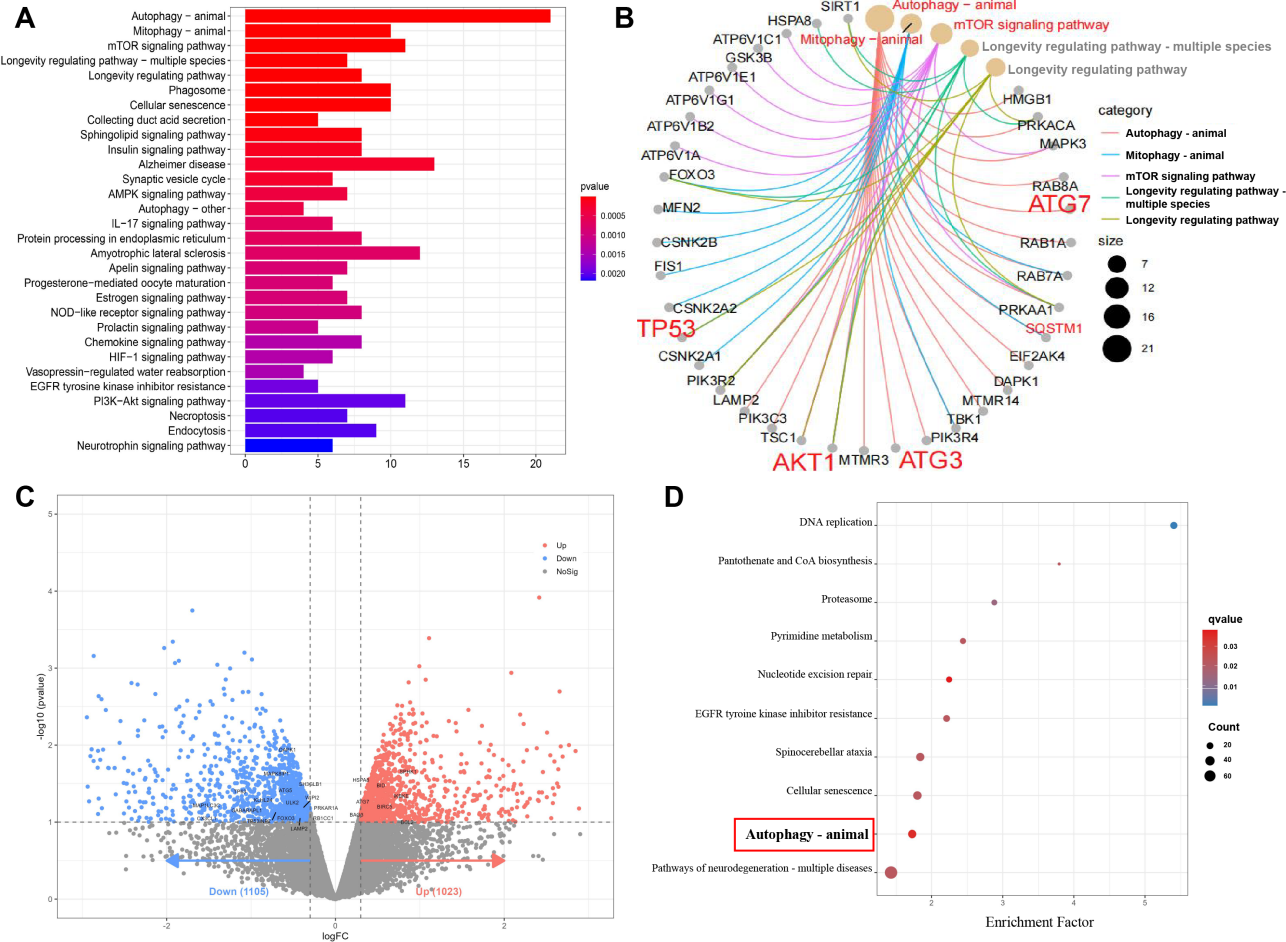
Investigating the correlation between TPX2 and the autophagy pathways. **(A)** KEGG pathway enrichment analysis of 337 autophagy-related proteins identified from 4,137 TPX2-interacting proteins by IP-MS. **(B)** Gene-pathway interaction network depicting the relationships between key differentially expressed genes and autophagy-related pathways, including autophagy, mitophagy, mTOR signaling, and longevity-regulating pathways. Nodes represent genes or pathways, edges indicate gene involvement in specific pathways, node size reflects connectivity, and colors denote pathway categories. **(C)** The volcano plot presented differentially expressed genes (DEGs) resulting from transcriptome analysis comparing shTPX2 and shNC PC9 cells. **(D)** A bubble pot showed the top 10 enriched KEGG pathways among the DEGs.

To investigate the molecular mechanisms underlying TPX2 function in LUAD cells, H1975 cells were transfected with TPX2-Flag and subjected to co-immunoprecipitation (Co-IP) using an anti-Flag antibody, as confirmed by Western blot analysis (**Supplementary Figure 3A**), followed by assessment with magnetic beads and Immunoprecipitation-Mass Spectrometry (IP-MS). This approach identified a total of 4,137 proteins associated with TPX2 by immunoprecipitation-mass spectrometry. Among these, 337 proteins were annotated as autophagy-related and were subjected to KEGG enrichment analysis. The analysis revealed that autophagy, mitophagy, and the mTOR signaling pathway were among the top enriched pathways (**Figure 3A**), suggesting a potential involvement of TPX2 in autophagy regulation. The chord diagrams provided a detailed visualization of the protein interactions within these pathways (**Figure 3B**).

In parallel, transcriptome sequencing was performed on shTPX2 PC9 cells, compared to shNC cells. A total of 1,023 upregulated and 1,105 downregulated differentially expressed genes (DEGs) were identified in the TPX2 knockdown group compared to the control group (**Figure 3C**). Subsequent KEGG enrichment analysis on the DEGs confirmed the significant enrichment pathways. The bubble plot depicted the top 10 enriched KEGG pathways among the DEGs, with autophagy prominently featured, further supporting the role of TPX2 in autophagy (**Figure 3D**). Together, the dual approach of IP-MS and transcriptome sequencing suggested a correlation between TPX2 and the regulation of autophagy in LUAD cells.

### 3.4 TPX2 promotes autophagic flux and induces the formation of autolysosome in LUAD

**Figure 4:**
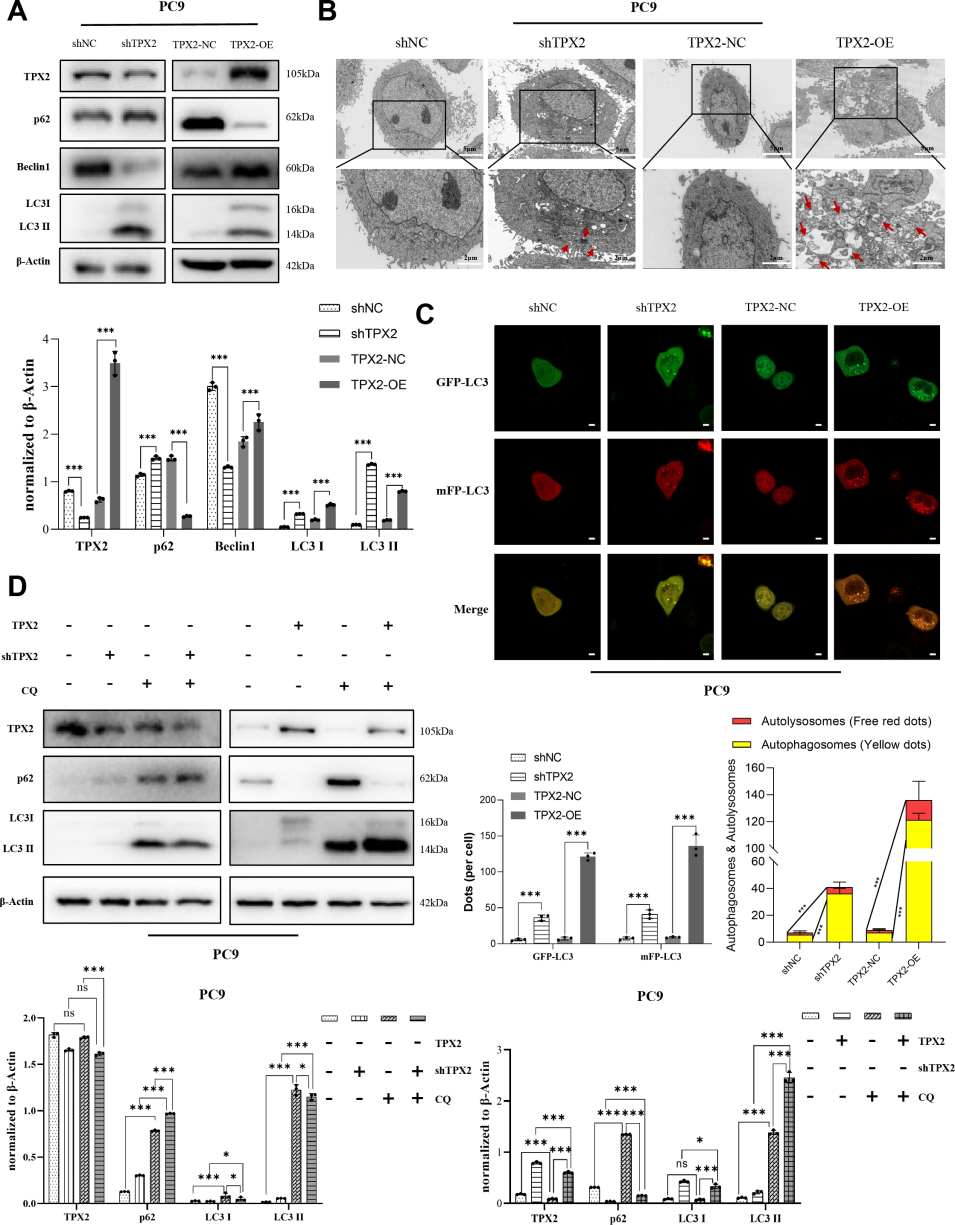
TPX2-mediated autophagy activation in LUAD. **(A)** Western blot analysis of autophagy-related markers (p62, Beclin1, and LC3B) in PC9 cells following TPX2 modulation. Densitometric quantification of the immunoblot bands was shown below. **(B)** Scanning electron microscopy images of shTPX2 and TPX2-OE PC9 cells, with arrows highlighting the presence of autophagosomes or autolysosomes. **(C)** GFP/mRFP-LC3 puncta assay assessed TPX2-mediated autophagy activation in shTPX2 and TPX2-OE PC9 cells. Yellow puncta (GFP+/mRFP+) indicated autophagosomes, whereas red puncta (mRFP+) indicated autolysosomes. LC3 puncta in individual cells from different treatment groups were quantified using ImageJ. Data are presented as mean ± SD from three independent experiments. **(D)** Analysis of autophagy markers p62, LC3B in shTPX2 and TPX2-OE PC9 cells treated with or without 10μM CQ. With densitometric quantification shown below as a bar graph. *p < 0.05, **p < 0.01, ***p < 0.001.

To investigate whether TPX2 regulates autophagic flux in lung cancer cells, we generated stable TPX2 knockdown (shTPX2) and overexpression (TPX2-OE) cell lines in PC9 and H1975 cells, respectively. Our findings showed that knockdown of TPX2 reduced Beclin-1 protein levels, concurrent with an increase in p62 and LC3-II, suggesting an impairment in autophagic flux. Conversely, TPX2 overexpression enhanced the Beclin-1 and LC3B-II levels, while p62 was downregulated (**Figure 4A**, and **Supplementary Figure S4A**), indicating the stimulatory effect of TPX2 on autophagy.

Transmission electron microscopy (TEM) was employed to visualize autophagosome and autolysosome formation (**Figure 4B** and **Supplementary Figure S4B**). In TPX2 knockdown cells, an accumulation of autophagosomes was observed, as indicated by the presence of numerous vesicles. In contrast, TPX2-overexpressing cells displayed a significant increase in autolysosomes, which is indicative of enhanced autophagic degradation.

A GFP-mRFP-LC3 puncta analysis was performed to further investigate the impact of TPX2 on autophagy. This assay revealed an increase in the number of GFP+/mRFP+ LC3 puncta (yellow dots) in TPX2 knockdown cells, suggesting the accumulation of autophagosomes. In TPX2-overexpressing cells, a notable presence of GFP-/mRFP+ puncta (red dots) was observed, which corresponds to the formation of autolysosomes (**Figures 4C** and **Supplementary Figure S4C**). These results suggested that TPX2 overexpression may facilitate autophagosome maturation and degradation.

To monitor autophagic flux, chloroquine (CQ), an autophagy inhibitor that suppresses lysosomal acidification and blocks autophagic degradation, leading to accumulation of LC3-II and p62. Treatment with CQ, in the absence or presence of TPX2 overexpression, revealed distinct patterns of autophagy marker accumulation. Upon CQ treatment, LC3-II accumulated in control cells as expected. In TPX2-knockdown cells, p62 showed a more pronounced accumulation, and the LC3-II accumulation pattern was consistent with impaired flux. Importantly, these interpretations are supported by the tandem GFP-mRFP-LC3 assay, which demonstrated increased GFP+/mRFP+ (yellow) puncta in TPX2-knockdown cells. Compared to CQ alone, the combination of TPX2 overexpression and CQ led to a further increase in LC3-II levels, suggesting that TPX2 overexpression enhances autophagic flux by promoting the formation of autolysosomes. As expected, p62 accumulated upon CQ treatment in both control and TPX2-OE cells. Compared with TPX2-OE alone, TPX2-OE + CQ showed increased p62, whereas compared with the corresponding control + CQ group, p62 remained relatively lower in TPX2-OE + CQ, consistent with enhanced upstream flux. Indicating that CQ-mediated blockade of autophagic degradation leads to p62 accumulation even in the context of TPX2 overexpression. While we cannot exclude the possibility that TPX2 may also influence p62 expression at the transcriptional level, the available data support a predominant contribution of altered autophagic flux. (**Figure 4D** and **Supplementary Figure S4D**). Collectively, TPX2 depletion led to reduced Beclin-1 and concomitant accumulation of p62 and LC3-II, together with increased GFP+/mRFP+ (yellow) puncta in the tandem mRFP-GFP-LC3 assay, indicating impaired autophagosome maturation/flux. In contrast, TPX2 overexpression decreased p62 and increased red-only puncta, consistent with enhanced autolysosome formation and flux. Upon CQ treatment, LC3-II and p62 accumulated as expected due to lysosomal inhibition; notably, TPX2-OE + CQ showed further LC3-II accumulation compared with control + CQ, supporting that TPX2 promotes upstream autophagosome formation/flux that becomes more apparent when degradation is pharmacologically blocked.

### 3.5 Chloroquine inhibited migration, proliferation, and stemness gene expression in LUAD cells

**Figure 5:**
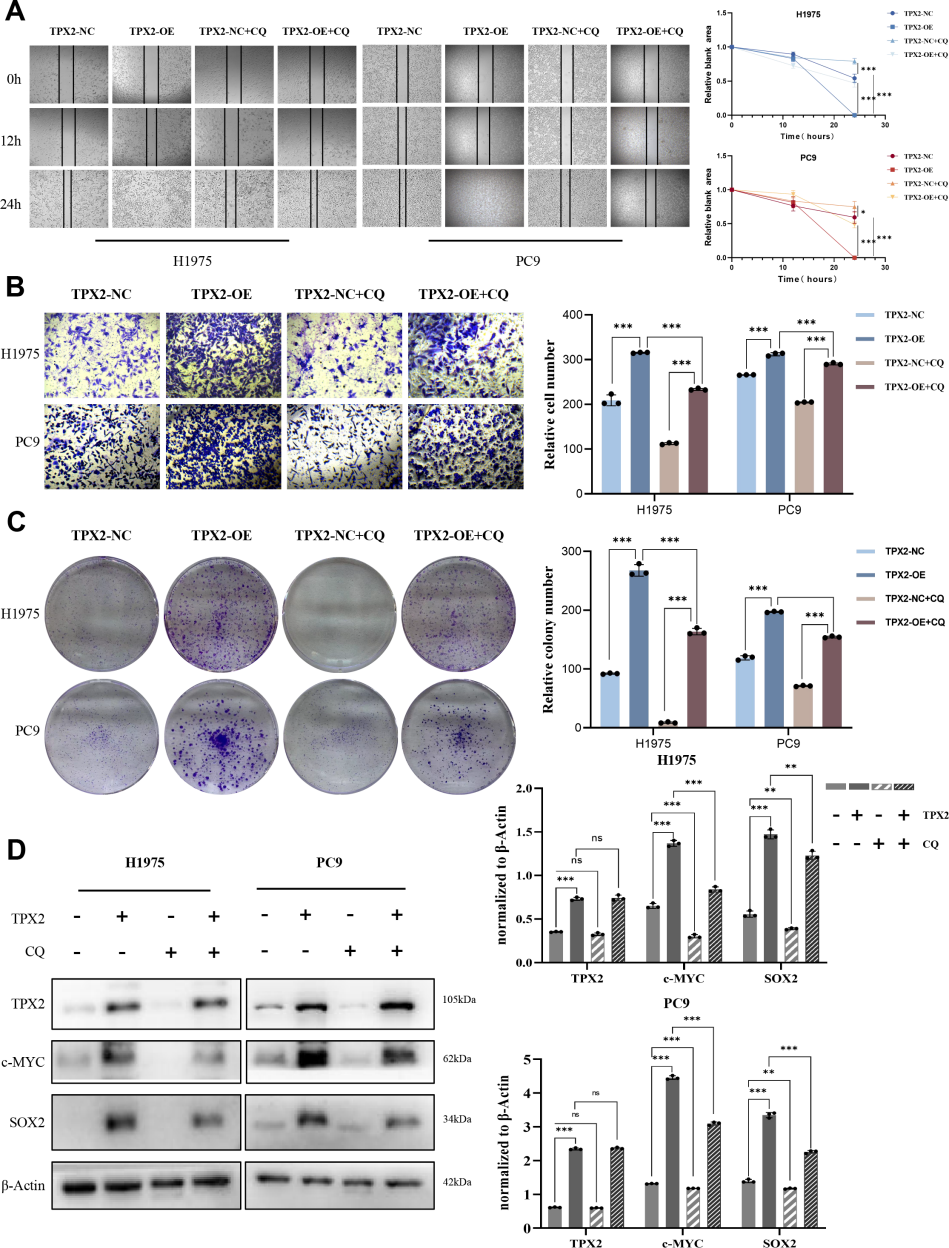
Modulatory effects of autophagy inhibition on LUAD cellular dynamics. **(A)** Wound-healing assays, **(B)** transwell migration assays, and **(C)** colony formation assays were conducted on TPX2-OE LUAD cells, treated with or without CQ (10μM). The right panels showed the quantitative analysis of the relative numbers of wound-healing, migration, and colony formation cells. **(D)** Western blot analysis of c-MYC and SOX2 expression in TPX2-OE LUAD cells, treated with or without CQ (10μM). The β-Actin-normalized gray values in the corresponding bands was shown on the right. *p < 0.05, **p < 0.01, ***p < 0.001.

To investigate the role of TPX2 in enhancing the oncogenic and stem-like characteristics of LUAD cells through autophagy, we employed CQ to modulate cellular behavior and assessed its impact on migration, proliferation, and stemness gene expression. Our data revealed that TPX2 overexpression enhanced the migratory ability of LUAD cells, while treatment with CQ significantly encountered the migration in both control and TPX2-OE groups (**Figure 5A**), indicating that CQ can counteract the pro-migratory effects of TPX2-OE. Moreover, CQ significantly diminished the invasion capability of LUAD cells (**Figure 5B**), suggesting that it may inhibit the enhanced invasion mediated by TPX2 overexpression. Similarly, TPX2-OE cells formed a significantly higher number of colonies than the control group, but this increase was abrogated by CQ treatment (**Figure 5C**). Furthermore, TPX2 overexpression was associated with increased protein levels of c-MYC and SOX2 (**Figure 5D**). CQ treatment reduced these levels, implying that autophagy, when inhibited by CQ, can reverse the stemness-promoting effects of TPX2 overexpression. Together, our study provides evidence that CQ can effectively antagonize the oncogenic effects of TPX2-OE in LUAD cells by inhibiting migration, proliferation, and stemness gene expression.

### 3.6 Silencing TPX2 expression improved the prognosis of LUAD xenograft tumor-bearing mice

**Figure 6:**
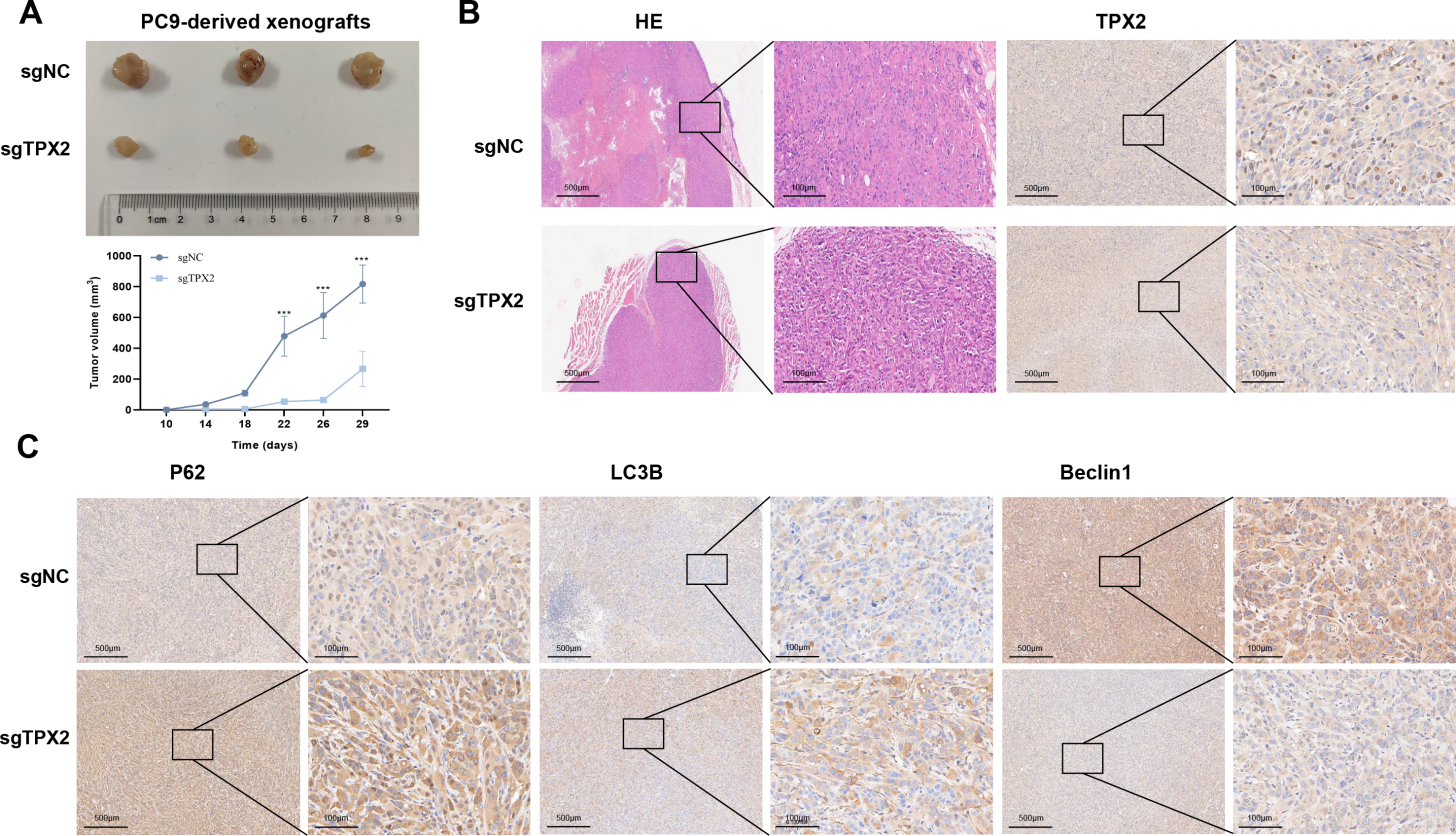
TPX2 depletion suppressed tumor growth *in vivo*. **(A)** Images of ex vivo transplanted tumors and a plot of the tumor volume curve in PC9 tumor-bearing mice. **(B)** Hematoxylin and eosin (H&E) staining and immunohistochemistry (IHC) analysis of TPX2 expression in PC9-derived xenograft tumors. **(C)** IHC assessment of p62, LC3B, Beclin1 in PC9-derived xenografts. ***p < 0.001.

Building upon *in vitro* observations, xenograft tumor models were established using PC9 and H1975 cell lines in nude mice, allowing evaluation of the effects of TPX2 on tumor growth *in vivo*. Four weeks following axillary inoculation, we noted a significant reduction in tumor size in the sgTPX2 group as compared to the control sgNC group (**Figure 6A** and **Supplementary Figure S5A**). HE staining was employed to examine the histological characteristics of the tumors. Our analysis revealed that the cellular morphology in the sgTPX2 group was maintained and appeared consistent with that of the sgNC group (**Figure 6B** and **Supplementary Figure S5B**). IHC staining for TPX2 demonstrated a marked decrease in protein expression in the sgTPX2 group, confirming the efficiency of TPX2 silencing (**Figure 6B**). Further, we conducted IHC to assess the expression levels of key proteins associated with autophagy (**Figure 6C** and **Supplementary Figure S5C**). We found that the protein levels of Beclin-1 was significantly lower in the sgTPX2 group compared to the sgNC group. Conversely, the expression of p62 and LC3-II was higher in the sgTPX2 group, indicating an alteration in autophagic flux (**Figure 6C**). These findings from *in vivo* experiments validated that TPX2 knockout not only inhibits tumor growth but also modulates the expression of proteins involved in autophagy.

## 4 Discussion

This study identifies TPX2 as a critical driver of LUAD progression, linking its overexpression to enhanced autophagy and cancer stemness. Functional and *in vivo* analyses demonstrated that TPX2 promotes autophagic flux and CSC marker expression, while its knockout impairs tumor growth and aggressiveness. Notably, autophagy inhibition via chloroquine counteracts TPX2-induced oncogenic effects, highlighting the therapeutic potential of targeting TPX2-mediated autophagy in LUAD. These findings provide new insights into the interplay between autophagy and cancer stemness. In addition, previous xenograft studies investigating TPX2 have predominantly relied on partial depletion strategies, which result in reduced but not fully abolished TPX2 expression (26). In the present LUAD xenograft model, CRISPR–Cas9-mediated knockout of TPX2 was applied to assess *in vivo* effects under conditions of more extensive TPX2 suppression. This experimental design extends existing xenograft studies by providing complementary *in vivo* evidence beyond that obtained from partial TPX2 depletion models.

In our study, TPX2’s role as an oncogene is supported by its overexpression in various tumors and its correlation with advanced tumor stages and poor prognosis. We further validated the association in clinical LUAD samples. While TPX2 has been implicated as a significant prognostic marker in LUAD (8), the underlying mechanisms of its oncogenic role remain unclear. Previous studies suggest that TPX2 exerts its oncogenic effects through multiple pathways. First, the formation of the Aurora-A/TPX2 complex, which is crucial for mitotic spindle assembly and stabilization, has been linked to tumor initiation and progression (2). Additionally, this complex has been shown to contribute to drug resistance to EGFR-TKIs in LUAD (1). There is also evidence indicating that TPX2 mediates dual resistance in hepatocellular carcinoma (HCC) cells to both tyrosine kinase inhibitors (TKIs) and conventional cytotoxic chemotherapy drugs (27). Given the critical role of autophagy in tumor drug resistance, elevated autophagic activity is frequently observed in drug-resistant tumor cells (28). Furthermore, TPX2 enhances the NF-κB/M-CSF signaling axis, thereby influencing M2 macrophage polarization and plays a critical role in reshaping the immune microenvironment (29). These findings collectively highlight the multifaceted role of TPX2 in LUAD progression. In our study, we assumed that TPX2 promoted the progression of LUAD through the promotion of autophagy and maintenance of tumor stemness.

Cancer stem cells (CSCs) are a subpopulation of tumor cells characterized by self-renewal capacity and tumor-initiating potential, which contribute to drug resistance, invasion, metastasis, and recurrence (30, 31). Emerging evidence supports TPX2 as a key regulator of tumor stemness. For instance, TPX2 enhances breast cancer progression via the PI3K/AKT pathway, a well-established CSCs regulatory axis (32, 33). Moreover, TPX2 modulates p53 signaling in bladder cancer, affecting proliferation, migration, and invasion (34). Since p53 is a crucial regulator of CSC differentiation, TPX2 may exert its effects on LUAD stemness by interfering with p53-mediated stemness suppression (35). In NSCLC, TPX2 has been reported to upregulate c-MYC expression by activating the Wnt/β-catenin pathway, which is strongly associated with metastasis and chemotherapy resistance (36). The Wnt/β-catenin/c-MYC/SOX2 axis has also been shown to sustain stemness in colorectal cancer (37). Consistent with these reports, our study demonstrated that TPX2 overexpression increases SOX2 and c-MYC expression and enhances tumor sphere formation in LUAD cells, reinforcing its role in stemness maintenance. However, further research is needed to determine whether TPX2 directly interacts with these pathways or exerts its effects indirectly via autophagy.

We observed that TPX2 enhances stemness via increased autophagic flux in LUAD. A growing body of evidence suggests that autophagy and CSC maintenance are intricately linked. Autophagy sustains CSCs by preventing senescence, enabling them to persist under metabolic stress and resist therapy (16). Likely, CSCs often exhibit elevated autophagic activity, as evidenced by high expression of autophagy-related markers (ATG5, Beclin-1) (38, 39). Our study further supports this notion, as TPX2 overexpression enhanced autophagic flux and stemness, while autophagy inhibition via CQ reduced c-MYC and SOX2 expression, leading to diminished CSC properties. However, the precise mechanisms underlying TPX2-mediated autophagy activation remain elusive. While TPX2 is known to activate the PI3K/AKT pathway (33, 40) and autophagy can be regulated through mTOR-independent mechanisms (41), further studies are required to clarify whether TPX2 directly regulates these pathways or exerts its effects through an alternative regulatory mechanism. In addition, given the well-established role of TPX2 in mitotic spindle assembly and cell cycle regulation (42), it is important to consider that alterations in TPX2 expression may also influence autophagy and stemness indirectly through changes in cell cycle dynamics. In the present study, we did not perform a systematic analysis of cell cycle distribution, and therefore cannot fully exclude the possibility that cell cycle arrest contributes, at least in part, to the observed phenotypes. While our data support a functional association between TPX2, autophagic flux, and CSC maintenance, further studies incorporating detailed cell cycle profiling will be required to distinguish direct autophagy-regulatory effects from secondary consequences of cell cycle perturbation.

Given TPX2’s dual role in autophagy regulation and CSC maintenance, targeting TPX2-mediated autophagy may represent a promising therapeutic approach for LUAD. Autophagy inhibitors, such as CQ and hydroxychloroquine (HCQ), have been shown to reduce CSC populations and enhance chemotherapy efficacy (43, 44). Our findings suggest that CQ effectively suppresses TPX2-induced stemness, highlighting the potential clinical relevance of combining TPX2-targeted therapy with autophagy inhibitors.

However, several challenges must be addressed before TPX2 can be considered a viable clinical target. Firstly, the investigation primarily relied on two LUAD cell lines, H1975 and PC9, which may not fully represent the genetic and phenotypic diversity of LUAD. A broader range of cell lines or patient-derived xenografts could enhance the generalizability of the findings. It should also be noted that TPX2 overexpression experiments were performed in LUAD cell lines that already display relatively high endogenous TPX2 expression. While this approach allowed us to further probe TPX2-associated gain-of-function effects, it may limit the physiological interpretation of the overexpression data. Future studies using low-TPX2–expressing or non-transformed cells will be valuable to further validate the role of TPX2 in LUAD progression. Secondly, as TPX2 plays a critical role in mitosis, systemic TPX2 inhibition may lead to toxic effects on normal proliferating cells. Strategies such as tumor-specific delivery mechanisms or synthetic lethal targeting could help mitigate these risks. Thirdly, while our study found that TPX2 is correlated with CSC markers, it did not directly prove whether TPX2 directly regulates the expression of SOX2/c-MYC, or whether this is merely a result of autophagy-induced stemness enhancement. Additionally, the specific mechanisms by which TPX2 regulates autophagy remain unclear. Further research is needed to elucidate these mechanisms and to determine the precise role of TPX2 in autophagy and stemness in LUAD.

## Data Availability

The original contributions presented in the study are publicly available. The RNA-seq data can be found in the GEO repository under accession number GSE333531. The IP-MS data are publicly available at https://doi.org/10.6084/m9.figshare.32415207.
