# Supplementary material for: TPX2-mediated autophagy maintains cancer stemness in LUAD: bioinformatic screening and functional validation
Source: Front Oncol. 2026 Jun 2;16:1724797. doi: 10.3389/fonc.2026.1724797 (PMC13269291; doi:10.3389/fonc.2026.1724797)
Supplement: Supplementary file 1 [file Image1.pdf]

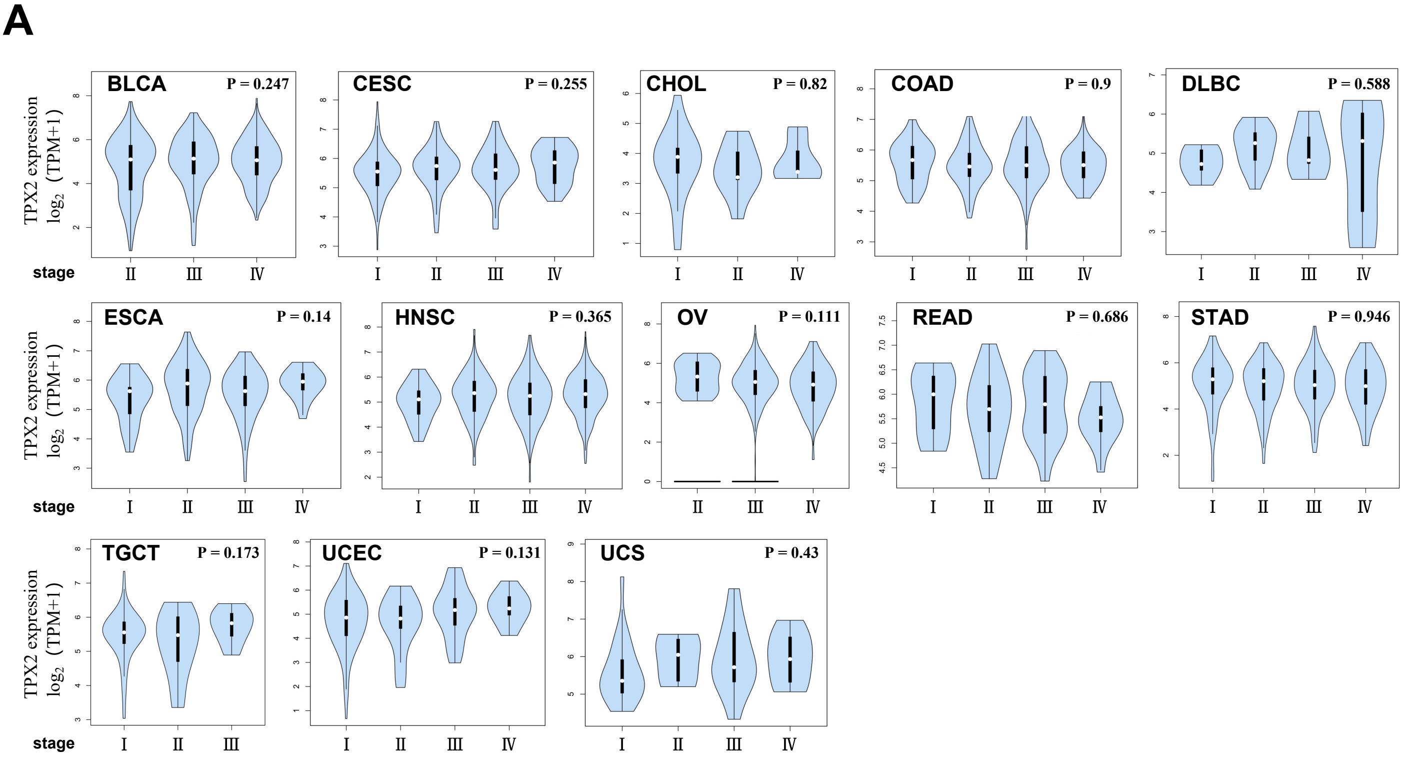

Supplementary Figure 1

Prognostic significance of TPX2 expression in LUAD. **(A)** Comparative analysis of TPX2 transcription levels across different pathological stages (I, II, III, and IV) in various cancers.
