# Supplementary material for: TPX2-mediated autophagy maintains cancer stemness in LUAD: bioinformatic screening and functional validation
Source: Front Oncol. 2026 Jun 2;16:1724797. doi: 10.3389/fonc.2026.1724797 (PMC13269291; doi:10.3389/fonc.2026.1724797)
Supplement: Supplementary file 2 [file Image2.pdf]

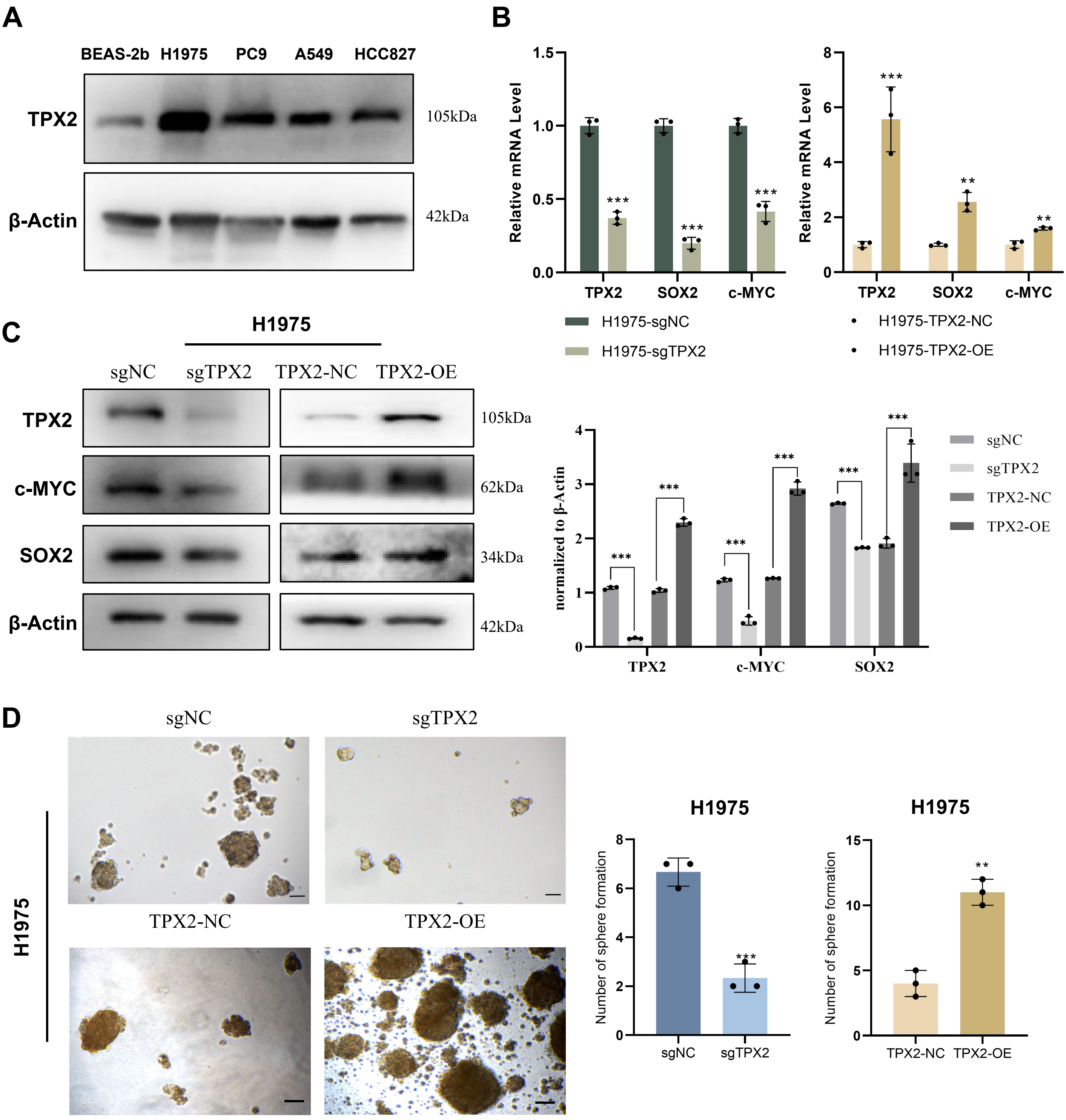

Supplementary Figure 2

TPX2 overexpression promoted the stemness of H1975 cells. **(A)** TPX2 protein expression levels in BEAS-2B and LUAD cells. **(B)** The mRNA and **(C)** protein expression levels of SOX2 and c-MYC in sgTPX2 and TPX2-OE H1975 cells. The figure below shows the β-Actin-normalized gray values of TPX2, c-MYC, and SOX2 in the corresponding Western blot bands. **(D)** Sphere formation ability analysis of sgTPX2 and TPX2-OE H1975 cells. Scale bars: 50μm. * $p < 0.05$, ** $p < 0.01$, *** $p < 0.001$.
