# Supplementary material for: TPX2-mediated autophagy maintains cancer stemness in LUAD: bioinformatic screening and functional validation
Source: Front Oncol. 2026 Jun 2;16:1724797. doi: 10.3389/fonc.2026.1724797 (PMC13269291; doi:10.3389/fonc.2026.1724797)
Supplement: Supplementary file 3 [file Image3.pdf]

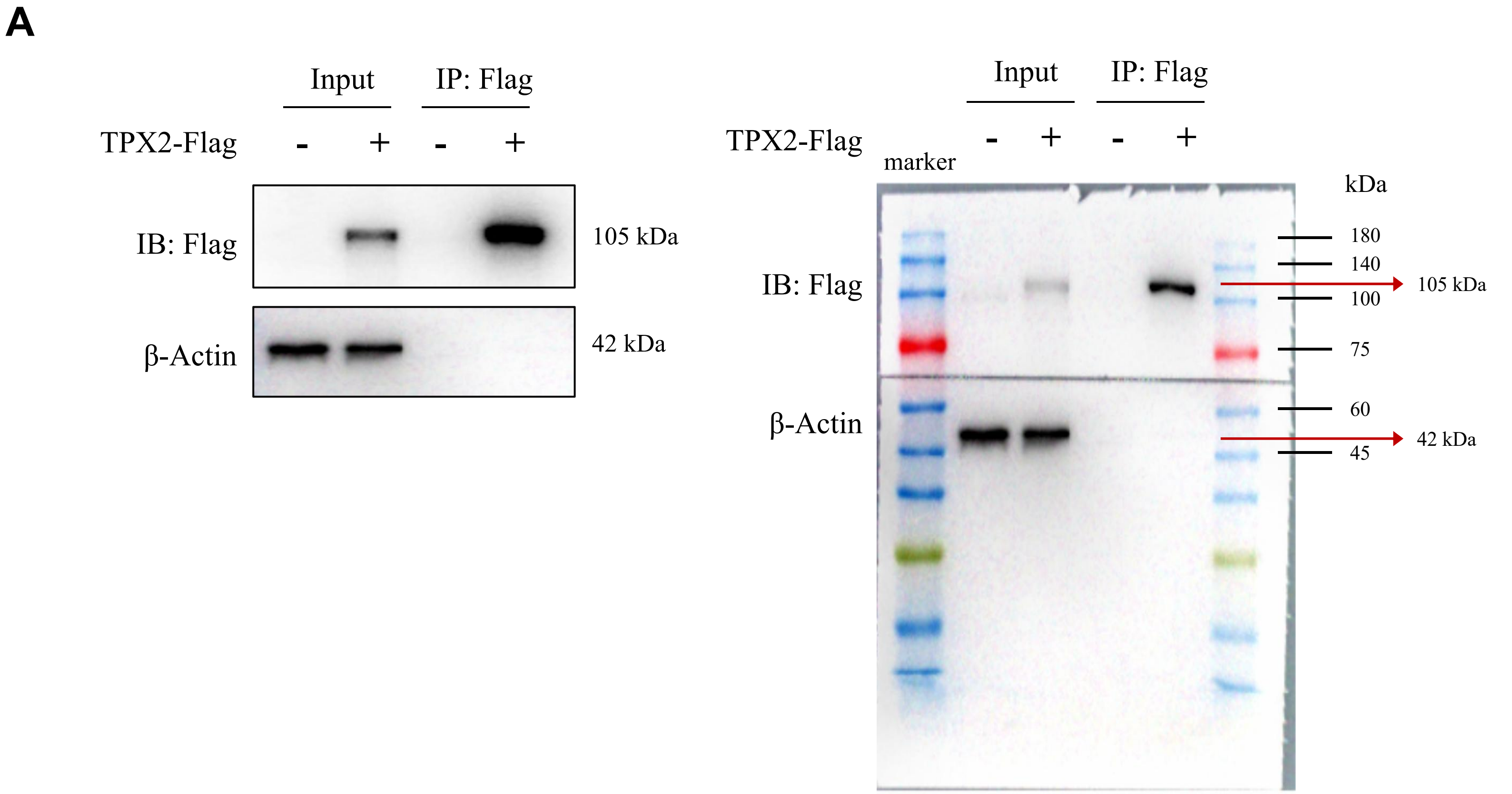

Supplementary Figure 3

Validation of TPX2-Flag immunoprecipitation. **(A)** Western blot analysis of TPX2-Flag expression in input and immunoprecipitated (IP) samples. The left panel shows TPX2-Flag protein at approximately 105 kDa in both input and IP samples, with β-Actin as a loading control at 42 kDa. The right panel displays the original. The input and IP samples were loaded in a 1:2 protein concentration ratio, and data are shown for Flag-tagged TPX2 overexpression (+) and control (-).
