# Supplementary material for: TPX2-mediated autophagy maintains cancer stemness in LUAD: bioinformatic screening and functional validation
Source: Front Oncol. 2026 Jun 2;16:1724797. doi: 10.3389/fonc.2026.1724797 (PMC13269291; doi:10.3389/fonc.2026.1724797)
Supplement: Supplementary file 4 [file Image4.pdf]

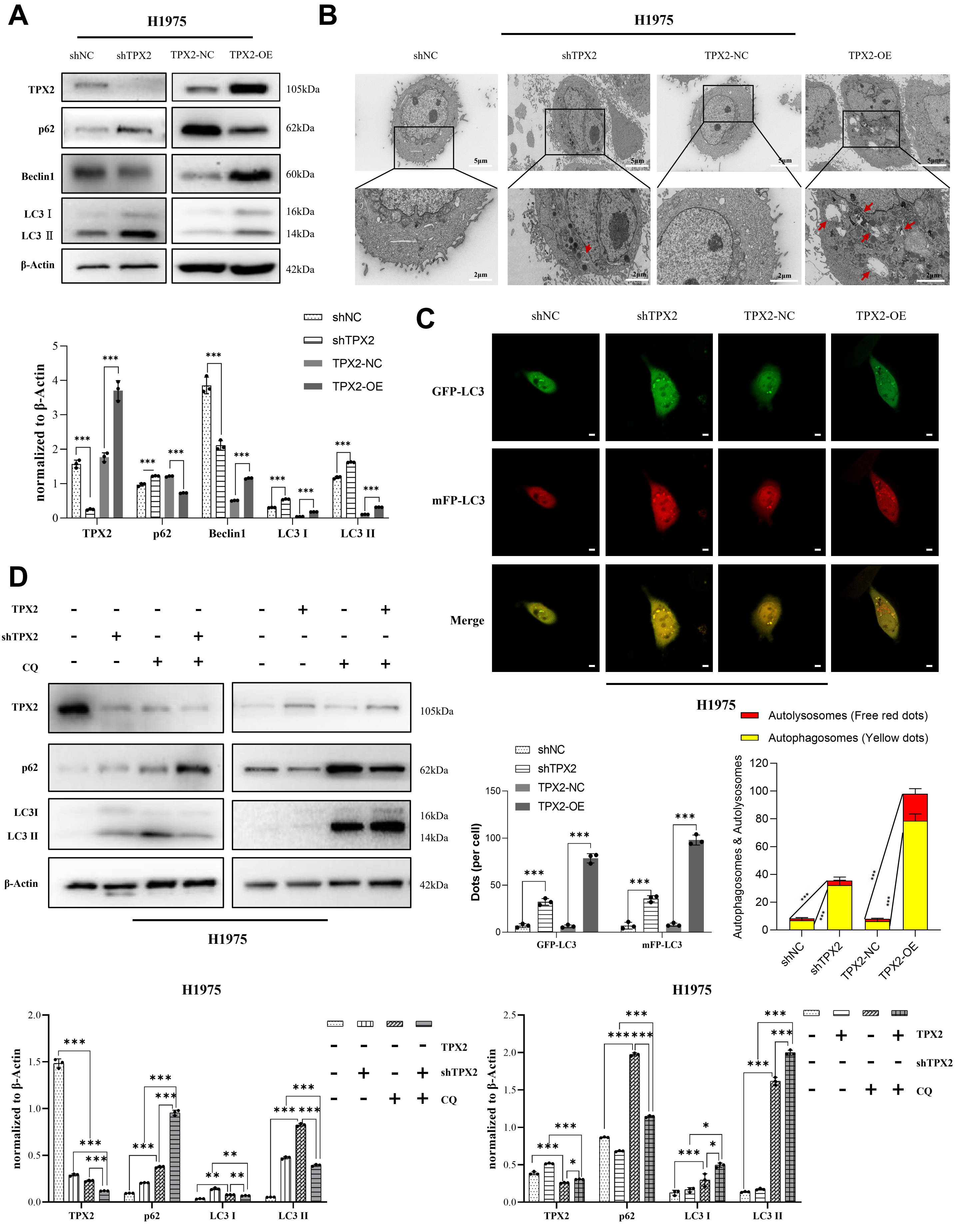

A
H1975
shNC
shTPX2
TPX2-NC
TPX2-OE
TPX2
p62
Beclin1
LC3 I
LC3 II
β-Actin
105kDa
62kDa
60kDa
16kDa
14kDa
42kDa
B
H1975
shNC
shTPX2
TPX2-NC
TPX2-OE
5μm
2μm
normalized to β-Actin
TPX2
p62
Beclin1
LC3 I
LC3 II
***
C
shNC
shTPX2
TPX2-NC
TPX2-OE
GFP-LC3
mFP-LC3
Merge
H1975
Autolysosomes (Free red dots)
Autophagosomes (Yellow dots)
Dots (per cell)
GFP-LC3
mFP-LC3
Autophagosomes & Autolysosomes
D
TPX2
shTPX2
CQ
TPX2
p62
LC3I
LC3 II
β-Actin
105kDa
62kDa
16kDa
14kDa
42kDa
H1975
H1975
normalized to β-Actin
TPX2
p62
LC3 I
LC3 II
H1975
normalized to β-Actin
TPX2
p62
LC3 I
LC3 II

Supplementary Figure 4

TPX2 promoted autophagy flux in H1975 cells. **(A)** Western blot analysis of autophagy-related markers (p62, Beclin1, and LC3B) in H1975 cells following TPX2 modulation. Densitometric quantification of the immunoblot bands was shown below. **(B)** Scanning electron microscopy images of shTPX2 and TPX2-OE H1975 cells, with arrows highlighting the presence of autophagosomes or autolysosomes. **(C)** GFP/mRFP-LC3 puncta assay assessed TPX2-mediated autophagy activation in shTPX2 and TPX2-OE H1975 cells. Yellow puncta (GFP+/mRFP+) indicated autophagosomes, whereas red puncta (mRFP+) indicated autolysosomes. LC3 puncta in individual cells from different treatment groups were quantified using ImageJ. Data are presented as mean ± SD from three independent experiments. **(D)** Analysis of autophagy markers p62, LC3B in shTPX2 and TPX2-OE H1975 cells treated with or without 10μM CQ. With densitometric quantification shown below as a bar graph.
