# Supplementary material for: TPX2-mediated autophagy maintains cancer stemness in LUAD: bioinformatic screening and functional validation
Source: Front Oncol. 2026 Jun 2;16:1724797. doi: 10.3389/fonc.2026.1724797 (PMC13269291; doi:10.3389/fonc.2026.1724797)
Supplement: Supplementary file 5 [file Image5.pdf]

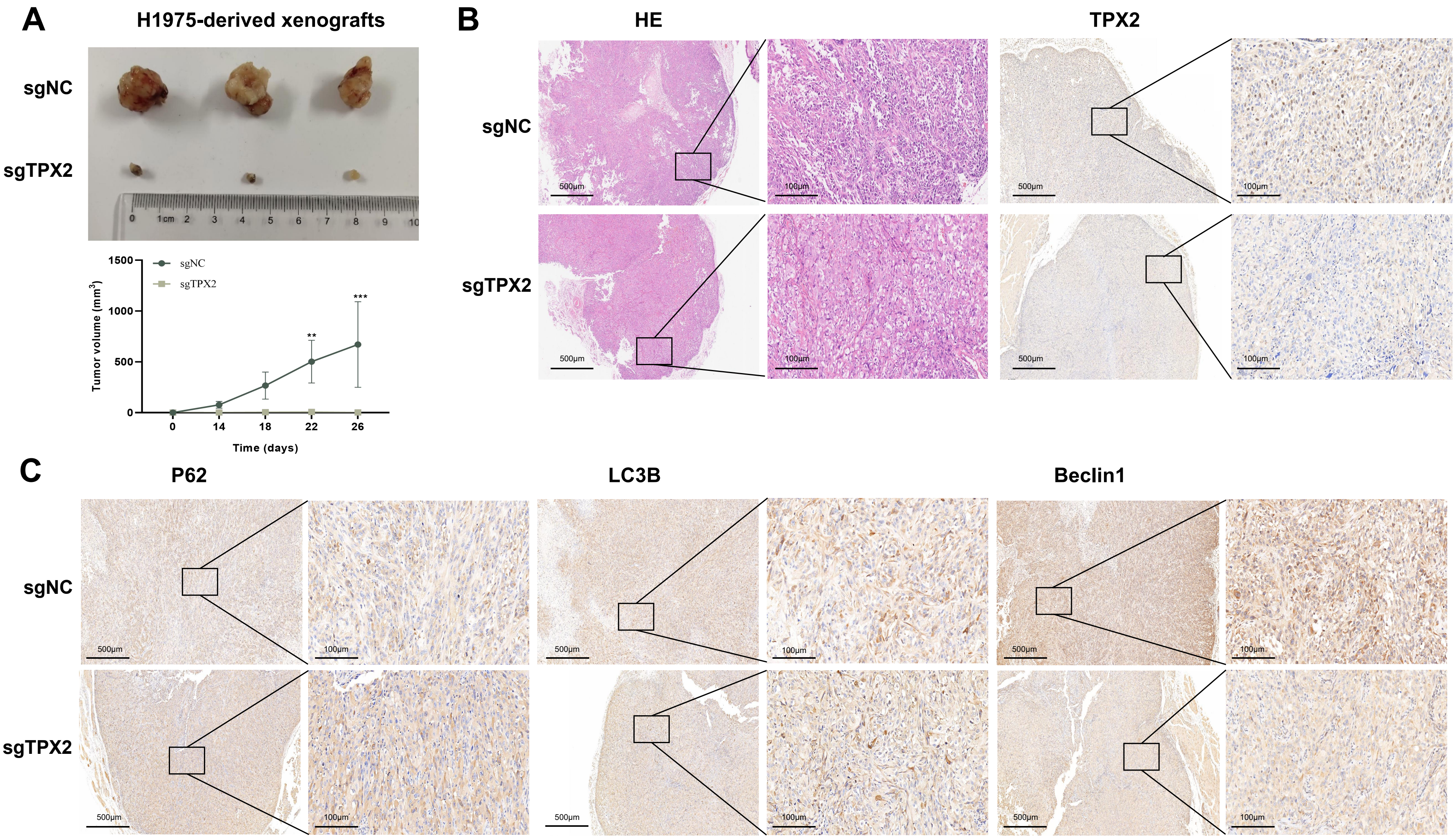

Supplementary Figure 5

TPX2 depletion suppressed tumor growth *in vivo*. **(A)** Images of excised xenograft tumors tumors and a plot of the tumor volume curve in H1975 tumor-bearing mice. **(B)** H&E staining and IHC analysis of TPX2 expression in H1975-derived xenograft tumors. **(C)** IHC assessment of p62, LC3B, Beclin1 in H1975-derived xenografts. $**p < 0.01$, $***p < 0.001$.
