# Supplementary material for: TPX2-mediated autophagy maintains cancer stemness in LUAD: bioinformatic screening and functional validation
Source: Front Oncol. 2026 Jun 2;16:1724797. doi: 10.3389/fonc.2026.1724797 (PMC13269291; doi:10.3389/fonc.2026.1724797)
Supplement: Supplementary file 6 [file Table1.docx]

| **Supplementary Table 1. The inserted sequences for plasmids construction** | |
| --- | --- |
| *Plasmids* | *Sequence (5’-3’)* |
| TPX2-RNAi | Forward oligo:  CCGGCGTGAACTTGATCCCAGAATACTCGAGTATTCTGGGATCAAGTTCACGTTTTTG |
|  | Reverse oligo:  AATTCAAAAACGTGAACTTGATCCCAGAATACTCGAGTATTCTGGGATCAAGTTCACG |
| pLKO.1-puro-shNC | Forward oligo:  CCGGCAGTACTTTTGTGTAGTACAACTCGAGTTGTACTACACAAAAGTACTGTTTTTG |
|  | Reverse oligo:  AATTCAAAAACAGTACTTTTGTGTAGTACAACTCGAGTTGTACTACACAAAAGTACTG |
