# Supplementary material for: TPX2-mediated autophagy maintains cancer stemness in LUAD: bioinformatic screening and functional validation
Source: Front Oncol. 2026 Jun 2;16:1724797. doi: 10.3389/fonc.2026.1724797 (PMC13269291; doi:10.3389/fonc.2026.1724797)
Supplement: Supplementary file 8 [file Table3.docx]

| **Supplementary table 3** | | | | |
| --- | --- | --- | --- | --- |
| **Gene name** | **Gene type** | **Fold change** | ***P*.Value** | ***P* adj value** |
| STARD4 | mRNA | -5.64 | 4.89E-03 | 7.38E-01 |
| VWA5A | mRNA | -5.10 | 5.07E-04 | 7.38E-01 |
| SLC1A6 | mRNA | -4.76 | 8.94E-04 | 7.38E-01 |
| TBC1D3 | mRNA | -4.64 | 1.99E-03 | 7.38E-01 |
| MAP9 | mRNA | -4.45 | 2.84E-03 | 7.38E-01 |
| ZNF559-ZNF177 | mRNA | -4.32 | 5.23E-02 | 7.38E-01 |
| SCN4B | mRNA | -3.89 | 8.48E-03 | 7.38E-01 |
| CGB2 | mRNA | -3.74 | 3.97E-03 | 7.38E-01 |
| EIF4EBP3 | mRNA | -3.69 | 2.25E-02 | 7.38E-01 |
| SCN2B | mRNA | -3.64 | 3.31E-03 | 7.38E-01 |
| SUPT20HL1 | mRNA | -3.50 | 1.73E-02 | 7.38E-01 |
| TBX4 | mRNA | -3.47 | 3.96E-02 | 7.38E-01 |
| GNMT | mRNA | -3.43 | 1.63E-02 | 7.38E-01 |
| HTR2C | mRNA | -3.40 | 7.65E-03 | 7.38E-01 |
| SLC2A3 | mRNA | -3.32 | 1.61E-04 | 7.19E-01 |
| BEST2 | mRNA | -3.27 | 5.94E-03 | 7.38E-01 |
| C8orf44-SGK3 | mRNA | -3.21 | 8.62E-02 | 7.42E-01 |
| PDIA2 | mRNA | -3.20 | 7.17E-03 | 7.38E-01 |
| SLC1A7 | mRNA | -3.17 | 1.37E-02 | 7.38E-01 |
| SCUBE1 | mRNA | -3.13 | 2.63E-02 | 7.38E-01 |
| CACNA1F | mRNA | -3.12 | 1.38E-02 | 7.38E-01 |
| SERPING1 | mRNA | -3.12 | 4.35E-02 | 7.38E-01 |
| NPIPA3 | mRNA | -3.11 | 7.89E-02 | 7.38E-01 |
| ADAMTS3 | mRNA | -3.10 | 2.02E-02 | 7.38E-01 |
| NKAIN4 | mRNA | -3.07 | 4.94E-02 | 7.38E-01 |
| TMEM59L | mRNA | -3.07 | 3.79E-02 | 7.38E-01 |
| MRC2 | mRNA | -3.05 | 1.30E-02 | 7.38E-01 |
| PAGE2B | mRNA | -3.04 | 1.12E-02 | 7.38E-01 |
| C1orf105 | mRNA | -3.04 | 1.13E-02 | 7.38E-01 |
| GRIP2 | mRNA | -3.00 | 2.77E-02 | 7.38E-01 |
| SYNE1 | mRNA | -2.95 | 4.35E-03 | 7.38E-01 |
| EXTL1 | mRNA | -2.94 | 3.46E-02 | 7.38E-01 |
| CACNA1S | mRNA | -2.92 | 5.38E-02 | 7.38E-01 |
| HLA-DRB1 | mRNA | -2.90 | 1.39E-02 | 7.38E-01 |
| UGT1A7 | mRNA | -2.89 | 1.13E-02 | 7.38E-01 |
| LEMD1 | mRNA | -2.89 | 1.14E-02 | 7.38E-01 |
| MAP3K7CL | mRNA | -2.87 | 1.61E-02 | 7.38E-01 |
| IFI16 | mRNA | -2.87 | 6.95E-04 | 7.38E-01 |
| ARMC12 | mRNA | -2.82 | 1.97E-02 | 7.38E-01 |
| NECAB1 | mRNA | -2.81 | 1.19E-02 | 7.38E-01 |
| VXN | mRNA | -2.81 | 7.85E-02 | 7.38E-01 |
| FAIM2 | mRNA | -2.81 | 2.31E-03 | 7.38E-01 |
| FUT5 | mRNA | -2.81 | 4.95E-02 | 7.38E-01 |
| ROBO1 | mRNA | -2.79 | 1.55E-02 | 7.38E-01 |
| OPRL1 | mRNA | -2.77 | 2.53E-03 | 7.38E-01 |
| LBP | mRNA | -2.77 | 6.59E-02 | 7.38E-01 |
| SMIM10L2A | mRNA | -2.75 | 1.14E-02 | 7.38E-01 |
| FLRT3 | mRNA | -2.72 | 3.50E-03 | 7.38E-01 |
| POF1B | mRNA | -2.69 | 1.97E-02 | 7.38E-01 |
| GOLGA8S | mRNA | -2.69 | 1.99E-02 | 7.38E-01 |
| DLX5 | mRNA | -2.62 | 1.98E-02 | 7.38E-01 |
| P2RX1 | mRNA | -2.62 | 1.98E-02 | 7.38E-01 |
| PPEF1 | mRNA | -2.62 | 1.98E-02 | 7.38E-01 |
| SDR9C7 | mRNA | -2.62 | 1.98E-02 | 7.38E-01 |
| ZDHHC19 | mRNA | -2.60 | 4.36E-02 | 7.38E-01 |
| FAM81B | mRNA | -2.56 | 9.35E-03 | 7.38E-01 |
| MMP14 | mRNA | -2.55 | 4.87E-03 | 7.38E-01 |
| BRDT | mRNA | -2.51 | 3.10E-02 | 7.38E-01 |
| INSL4 | mRNA | -2.51 | 7.72E-02 | 7.38E-01 |
| RPL17-C18orf32 | mRNA | -2.51 | 1.47E-02 | 7.38E-01 |
| IGFBP5 | mRNA | -2.48 | 9.11E-06 | 1.47E-01 |
| CPNE6 | mRNA | -2.48 | 5.38E-02 | 7.38E-01 |
| PIWIL4 | mRNA | -2.48 | 3.17E-02 | 7.38E-01 |
| OSBPL6 | mRNA | -2.47 | 3.17E-02 | 7.38E-01 |
| DUX4 | mRNA | -2.46 | 2.16E-02 | 7.38E-01 |
| TBPL2 | mRNA | -2.46 | 2.16E-02 | 7.38E-01 |
| GZMM | mRNA | -2.46 | 2.17E-02 | 7.38E-01 |
| HNF4G | mRNA | -2.46 | 2.17E-02 | 7.38E-01 |
| ICAM2 | mRNA | -2.46 | 2.17E-02 | 7.38E-01 |
| NELL2 | mRNA | -2.46 | 2.17E-02 | 7.38E-01 |
| NT5C1B | mRNA | -2.46 | 2.17E-02 | 7.38E-01 |
| PCDHGB1 | mRNA | -2.46 | 2.17E-02 | 7.38E-01 |
| PAEP | mRNA | -2.45 | 4.10E-02 | 7.38E-01 |
| KRT34 | mRNA | -2.45 | 6.47E-02 | 7.38E-01 |
| LPL | mRNA | -2.42 | 1.56E-03 | 7.38E-01 |
| IP6K3 | mRNA | -2.42 | 9.10E-02 | 7.42E-01 |
| GLI2 | mRNA | -2.40 | 2.35E-02 | 7.38E-01 |
| GOLGA8J | mRNA | -2.39 | 3.44E-02 | 7.38E-01 |
| CTCFL | mRNA | -2.38 | 7.19E-02 | 7.38E-01 |
| C5orf47 | mRNA | -2.37 | 4.04E-02 | 7.38E-01 |
| CLDN2 | mRNA | -2.37 | 4.04E-02 | 7.38E-01 |
| CNGA4 | mRNA | -2.37 | 4.04E-02 | 7.38E-01 |
| CPXM2 | mRNA | -2.37 | 4.04E-02 | 7.38E-01 |
| SLC22A11 | mRNA | -2.37 | 4.04E-02 | 7.38E-01 |
| UNC93A | mRNA | -2.37 | 4.04E-02 | 7.38E-01 |
| CFH | mRNA | -2.34 | 1.63E-03 | 7.38E-01 |
| DKK3 | mRNA | -2.34 | 7.59E-03 | 7.38E-01 |
| GOLGA8O | mRNA | -2.34 | 6.45E-03 | 7.38E-01 |
| ATP12A | mRNA | -2.34 | 9.61E-02 | 7.42E-01 |
| FBLN2 | mRNA | -2.32 | 8.70E-02 | 7.42E-01 |
| CD207 | mRNA | -2.32 | 2.11E-02 | 7.38E-01 |
| HLA-DQB1 | mRNA | -2.32 | 5.41E-02 | 7.38E-01 |
| KLK13 | mRNA | -2.30 | 6.42E-02 | 7.38E-01 |
| GJA3 | mRNA | -2.28 | 5.54E-02 | 7.38E-01 |
| COL4A4 | mRNA | -2.27 | 4.77E-03 | 7.38E-01 |
| HGFAC | mRNA | -2.26 | 2.80E-02 | 7.38E-01 |
| NPPC | mRNA | -2.26 | 2.80E-02 | 7.38E-01 |
| SERPINB2 | mRNA | -2.26 | 2.80E-02 | 7.38E-01 |
| MAGEA2 | mRNA | -2.25 | 2.67E-02 | 7.38E-01 |
| LMO1 | mRNA | -2.25 | 5.51E-02 | 7.38E-01 |
| CD52 | mRNA | -2.24 | 8.81E-02 | 7.42E-01 |
| PIK3AP1 | mRNA | -2.22 | 1.59E-02 | 7.38E-01 |
| ADGRD2 | mRNA | -2.22 | 4.11E-02 | 7.38E-01 |
| CALHM5 | mRNA | -2.22 | 4.11E-02 | 7.38E-01 |
| CST7 | mRNA | -2.22 | 4.11E-02 | 7.38E-01 |
| EIF4E1B | mRNA | -2.22 | 4.11E-02 | 7.38E-01 |
| PPIAL4G | mRNA | -2.22 | 4.11E-02 | 7.38E-01 |
| SLAMF9 | mRNA | -2.22 | 4.11E-02 | 7.38E-01 |
| MSI1 | mRNA | -2.21 | 9.92E-02 | 7.42E-01 |
| CCDC89 | mRNA | -2.21 | 4.15E-02 | 7.38E-01 |
| RADIL | mRNA | -2.21 | 4.15E-02 | 7.38E-01 |
| TAC4 | mRNA | -2.21 | 4.15E-02 | 7.38E-01 |
| TMEM176B | mRNA | -2.21 | 4.15E-02 | 7.38E-01 |
| GBP6 | mRNA | -2.21 | 3.68E-02 | 7.38E-01 |
| ADAMTSL4 | mRNA | -2.19 | 1.83E-02 | 7.38E-01 |
| IGFALS | mRNA | -2.19 | 8.98E-02 | 7.42E-01 |
| SLC25A52 | mRNA | -2.19 | 8.98E-02 | 7.42E-01 |
| SYPL2 | mRNA | -2.16 | 4.81E-02 | 7.38E-01 |
| CNGA3 | mRNA | -2.16 | 5.76E-02 | 7.38E-01 |
| TRIM15 | mRNA | -2.16 | 5.76E-02 | 7.38E-01 |
| LGI2 | mRNA | -2.16 | 5.80E-02 | 7.38E-01 |
| UNC13B | mRNA | -2.14 | 2.07E-02 | 7.38E-01 |
| ARMCX3 | mRNA | -2.13 | 2.17E-03 | 7.38E-01 |
| SLC6A12 | mRNA | -2.13 | 2.25E-02 | 7.38E-01 |
| IL2RG | mRNA | -2.13 | 2.91E-02 | 7.38E-01 |
| COL5A1 | mRNA | -2.11 | 2.51E-02 | 7.38E-01 |
| FGD5 | mRNA | -2.10 | 8.14E-02 | 7.38E-01 |
| ITGAX | mRNA | -2.10 | 7.24E-02 | 7.38E-01 |
| SELENOP | mRNA | -2.08 | 5.80E-03 | 7.38E-01 |
| KCNK4 | mRNA | -2.07 | 9.19E-02 | 7.42E-01 |
| VWCE | mRNA | -2.07 | 9.19E-02 | 7.42E-01 |
| LGI4 | mRNA | -2.06 | 7.32E-02 | 7.38E-01 |
| ADAMTS12 | mRNA | -2.05 | 5.73E-03 | 7.38E-01 |
| CLCNKB | mRNA | -2.05 | 2.85E-02 | 7.38E-01 |
| COL4A3 | mRNA | -2.05 | 7.90E-03 | 7.38E-01 |
| C12orf75 | mRNA | -2.04 | 4.41E-02 | 7.38E-01 |
| AOC1 | mRNA | -2.03 | 5.48E-04 | 7.38E-01 |
| ESRRG | mRNA | -2.02 | 4.70E-02 | 7.38E-01 |
| FAM83F | mRNA | -2.02 | 4.70E-02 | 7.38E-01 |
| LRP2 | mRNA | -2.02 | 4.70E-02 | 7.38E-01 |
| P3H3 | mRNA | -2.02 | 4.70E-02 | 7.38E-01 |
| SAMD3 | mRNA | -2.02 | 4.70E-02 | 7.38E-01 |
| SDK2 | mRNA | -2.02 | 4.70E-02 | 7.38E-01 |
| SLC38A4 | mRNA | -2.02 | 4.70E-02 | 7.38E-01 |
| CAMK1G | mRNA | -2.01 | 4.72E-02 | 7.38E-01 |
| HOXA13 | mRNA | -2.01 | 4.72E-02 | 7.38E-01 |
| MCEMP1 | mRNA | -2.01 | 4.72E-02 | 7.38E-01 |
| NXF3 | mRNA | -2.01 | 4.72E-02 | 7.38E-01 |
| OMP | mRNA | -2.01 | 4.72E-02 | 7.38E-01 |
| SPATA45 | mRNA | -2.01 | 4.72E-02 | 7.38E-01 |
| VSIG8 | mRNA | -2.01 | 4.72E-02 | 7.38E-01 |
| PDZD2 | mRNA | -2.01 | 6.61E-03 | 7.38E-01 |
| SLC34A2 | mRNA | -2.01 | 9.02E-02 | 7.42E-01 |
| OR2F1 | mRNA | -2.01 | 6.56E-02 | 7.38E-01 |
| KLK11 | mRNA | -2.00 | 6.60E-02 | 7.38E-01 |
| RASD1 | mRNA | -1.98 | 2.08E-02 | 7.38E-01 |
| RENBP | mRNA | -1.96 | 9.25E-02 | 7.42E-01 |
| DUOXA1 | mRNA | -1.96 | 9.31E-02 | 7.42E-01 |
| GRIA2 | mRNA | -1.96 | 9.31E-02 | 7.42E-01 |
| GPR17 | mRNA | -1.95 | 4.83E-02 | 7.38E-01 |
| CACNG6 | mRNA | -1.94 | 3.76E-02 | 7.38E-01 |
| UNC13C | mRNA | -1.93 | 7.16E-02 | 7.38E-01 |
| GCNT1 | mRNA | -1.93 | 4.54E-04 | 7.38E-01 |
| NEU4 | mRNA | -1.93 | 8.06E-02 | 7.38E-01 |
| RCAN2 | mRNA | -1.92 | 3.30E-02 | 7.38E-01 |
| CFI | mRNA | -1.92 | 9.83E-02 | 7.42E-01 |
| NEURL1 | mRNA | -1.90 | 8.59E-04 | 7.38E-01 |
| CILP2 | mRNA | -1.89 | 3.70E-02 | 7.38E-01 |
| FLNC | mRNA | -1.89 | 3.42E-03 | 7.38E-01 |
| SVEP1 | mRNA | -1.86 | 8.03E-04 | 7.38E-01 |
| DNAI1 | mRNA | -1.85 | 1.45E-02 | 7.38E-01 |
| MSN | mRNA | -1.85 | 3.17E-03 | 7.38E-01 |
| FOLR1 | mRNA | -1.85 | 5.72E-03 | 7.38E-01 |
| CCDC153 | mRNA | -1.83 | 1.17E-02 | 7.38E-01 |
| GPX3 | mRNA | -1.82 | 5.15E-03 | 7.38E-01 |
| EMID1 | mRNA | -1.80 | 6.78E-03 | 7.38E-01 |
| CLEC2B | mRNA | -1.80 | 5.47E-02 | 7.38E-01 |
| GPM6A | mRNA | -1.80 | 4.82E-02 | 7.38E-01 |
| EMB | mRNA | -1.80 | 4.14E-03 | 7.38E-01 |
| NMU | mRNA | -1.79 | 5.56E-02 | 7.38E-01 |
| ETV1 | mRNA | -1.79 | 6.44E-02 | 7.38E-01 |
| ARHGAP40 | mRNA | -1.77 | 6.67E-02 | 7.38E-01 |
| CSDC2 | mRNA | -1.77 | 6.67E-02 | 7.38E-01 |
| FAM133A | mRNA | -1.77 | 6.67E-02 | 7.38E-01 |
| FAT4 | mRNA | -1.77 | 6.67E-02 | 7.38E-01 |
| KCNS1 | mRNA | -1.77 | 6.67E-02 | 7.38E-01 |
| MAOB | mRNA | -1.77 | 6.67E-02 | 7.38E-01 |
| MORN5 | mRNA | -1.77 | 6.67E-02 | 7.38E-01 |
| MT1E | mRNA | -1.77 | 6.67E-02 | 7.38E-01 |
| NNMT | mRNA | -1.77 | 6.67E-02 | 7.38E-01 |
| PKD1L3 | mRNA | -1.77 | 6.67E-02 | 7.38E-01 |
| PYY | mRNA | -1.77 | 6.67E-02 | 7.38E-01 |
| RHOH | mRNA | -1.77 | 6.67E-02 | 7.38E-01 |
| SEC16B | mRNA | -1.77 | 6.67E-02 | 7.38E-01 |
| TM4SF4 | mRNA | -1.77 | 6.67E-02 | 7.38E-01 |
| TAC3 | mRNA | -1.77 | 9.11E-02 | 7.42E-01 |
| TIGIT | mRNA | -1.76 | 2.93E-02 | 7.38E-01 |
| KRT17 | mRNA | -1.76 | 2.64E-03 | 7.38E-01 |
| RAB7B | mRNA | -1.75 | 2.43E-02 | 7.38E-01 |
| CALHM3 | mRNA | -1.75 | 1.42E-02 | 7.38E-01 |
| ARMCX2 | mRNA | -1.73 | 7.47E-02 | 7.38E-01 |
| FNDC4 | mRNA | -1.73 | 4.56E-02 | 7.38E-01 |
| A1BG | mRNA | -1.72 | 2.87E-02 | 7.38E-01 |
| GPR173 | mRNA | -1.72 | 6.28E-02 | 7.38E-01 |
| LMOD1 | mRNA | -1.72 | 4.62E-02 | 7.38E-01 |
| LHX9 | mRNA | -1.71 | 8.31E-03 | 7.38E-01 |
| TNNT2 | mRNA | -1.71 | 5.50E-02 | 7.38E-01 |
| MMP1 | mRNA | -1.71 | 7.75E-02 | 7.38E-01 |
| TLE4 | mRNA | -1.71 | 3.84E-02 | 7.38E-01 |
| C4A | mRNA | -1.70 | 1.19E-02 | 7.38E-01 |
| TGFB2 | mRNA | -1.70 | 1.20E-02 | 7.38E-01 |
| COL12A1 | mRNA | -1.70 | 1.79E-04 | 7.19E-01 |
| TCAP | mRNA | -1.70 | 9.21E-02 | 7.42E-01 |
| IQUB | mRNA | -1.69 | 5.21E-02 | 7.38E-01 |
| PROS1 | mRNA | -1.69 | 7.67E-03 | 7.38E-01 |
| SCARA5 | mRNA | -1.68 | 5.61E-02 | 7.38E-01 |
| HPN | mRNA | -1.65 | 4.46E-03 | 7.38E-01 |
| C4B | mRNA | -1.63 | 3.29E-02 | 7.38E-01 |
| MRAS | mRNA | -1.61 | 3.70E-03 | 7.38E-01 |
| ZNF843 | mRNA | -1.61 | 4.41E-02 | 7.38E-01 |
| CAMK2A | mRNA | -1.61 | 7.44E-02 | 7.38E-01 |
| EFS | mRNA | -1.61 | 3.54E-02 | 7.38E-01 |
| RASSF2 | mRNA | -1.61 | 3.04E-03 | 7.38E-01 |
| HMCN1 | mRNA | -1.60 | 2.29E-02 | 7.38E-01 |
| ITIH4 | mRNA | -1.59 | 6.24E-02 | 7.38E-01 |
| ZBP1 | mRNA | -1.59 | 5.97E-02 | 7.38E-01 |
| FAM89A | mRNA | -1.59 | 7.57E-02 | 7.38E-01 |
| SMIM11A | mRNA | -1.59 | 9.07E-02 | 7.42E-01 |
| SEMA5B | mRNA | -1.58 | 3.87E-02 | 7.38E-01 |
| COL6A2 | mRNA | -1.58 | 7.03E-02 | 7.38E-01 |
| PNCK | mRNA | -1.57 | 3.69E-02 | 7.38E-01 |
| RBM11 | mRNA | -1.56 | 5.83E-02 | 7.38E-01 |
| SYNPO2L | mRNA | -1.56 | 9.75E-03 | 7.38E-01 |
| KLF9 | mRNA | -1.56 | 2.80E-02 | 7.38E-01 |
| RAD21L1 | mRNA | -1.55 | 6.94E-02 | 7.38E-01 |
| SPARC | mRNA | -1.55 | 1.90E-02 | 7.38E-01 |
| SPATA6 | mRNA | -1.55 | 6.56E-02 | 7.38E-01 |
| SMIM10L2B | mRNA | -1.55 | 1.34E-02 | 7.38E-01 |
| KCNH1 | mRNA | -1.54 | 5.70E-02 | 7.38E-01 |
| CPS1 | mRNA | -1.54 | 4.55E-02 | 7.38E-01 |
| IRX2 | mRNA | -1.52 | 5.83E-02 | 7.38E-01 |
| HSPB7 | mRNA | -1.52 | 4.70E-02 | 7.38E-01 |
| RUNDC3B | mRNA | -1.51 | 5.21E-02 | 7.38E-01 |
| ALPG | mRNA | -1.51 | 1.12E-02 | 7.38E-01 |
| SYNDIG1L | mRNA | -1.51 | 8.22E-02 | 7.38E-01 |
| SV2A | mRNA | -1.51 | 5.94E-02 | 7.38E-01 |
| GPC4 | mRNA | -1.50 | 5.74E-02 | 7.38E-01 |
| COL15A1 | mRNA | -1.50 | 3.24E-02 | 7.38E-01 |
| FGD1 | mRNA | -1.49 | 2.08E-02 | 7.38E-01 |
| GPR162 | mRNA | -1.49 | 4.55E-02 | 7.38E-01 |
| SSC4D | mRNA | -1.49 | 2.23E-02 | 7.38E-01 |
| MUC15 | mRNA | -1.49 | 3.93E-02 | 7.38E-01 |
| MYL9 | mRNA | -1.48 | 2.69E-03 | 7.38E-01 |
| C1QTNF12 | mRNA | -1.48 | 2.84E-02 | 7.38E-01 |
| CTXND1 | mRNA | -1.48 | 6.70E-02 | 7.38E-01 |
| DMD | mRNA | -1.47 | 1.86E-02 | 7.38E-01 |
| CREB5 | mRNA | -1.46 | 2.32E-02 | 7.38E-01 |
| ARHGAP31 | mRNA | -1.46 | 2.98E-03 | 7.38E-01 |
| PRSS35 | mRNA | -1.44 | 9.61E-02 | 7.42E-01 |
| PDLIM4 | mRNA | -1.44 | 4.44E-02 | 7.38E-01 |
| MAP1LC3C | mRNA | -1.43 | 5.05E-02 | 7.38E-01 |
| IFITM2 | mRNA | -1.43 | 7.08E-03 | 7.38E-01 |
| FN1 | mRNA | -1.43 | 7.32E-03 | 7.38E-01 |
| CEACAM21 | mRNA | -1.43 | 7.42E-02 | 7.38E-01 |
| CX3CL1 | mRNA | -1.43 | 7.42E-02 | 7.38E-01 |
| FYN | mRNA | -1.43 | 1.44E-02 | 7.38E-01 |
| TMEM63C | mRNA | -1.42 | 8.04E-02 | 7.38E-01 |
| AVPR2 | mRNA | -1.42 | 3.36E-02 | 7.38E-01 |
| SLC6A11 | mRNA | -1.41 | 9.47E-02 | 7.42E-01 |
| PDCD1 | mRNA | -1.41 | 9.38E-02 | 7.42E-01 |
| PLA2G4A | mRNA | -1.41 | 1.86E-02 | 7.38E-01 |
| VGLL3 | mRNA | -1.41 | 9.57E-03 | 7.38E-01 |
| SUSD2 | mRNA | -1.40 | 9.04E-04 | 7.38E-01 |
| GDA | mRNA | -1.40 | 7.25E-02 | 7.38E-01 |
| KCTD12 | mRNA | -1.40 | 3.14E-02 | 7.38E-01 |
| TMEM98 | mRNA | -1.40 | 2.81E-02 | 7.38E-01 |
| DAB2 | mRNA | -1.39 | 7.52E-03 | 7.38E-01 |
| TMPRSS3 | mRNA | -1.38 | 9.60E-03 | 7.38E-01 |
| CPE | mRNA | -1.38 | 3.55E-02 | 7.38E-01 |
| GBP1 | mRNA | -1.37 | 4.31E-02 | 7.38E-01 |
| SOHLH2 | mRNA | -1.37 | 9.84E-02 | 7.42E-01 |
| TRPC1 | mRNA | -1.37 | 6.23E-02 | 7.38E-01 |
| PLXNC1 | mRNA | -1.37 | 3.32E-02 | 7.38E-01 |
| HMGCS1 | mRNA | -1.37 | 6.51E-02 | 7.38E-01 |
| LGALS2 | mRNA | -1.37 | 8.03E-02 | 7.38E-01 |
| BASP1 | mRNA | -1.36 | 1.53E-02 | 7.38E-01 |
| PRRT4 | mRNA | -1.36 | 2.27E-02 | 7.38E-01 |
| TMEM71 | mRNA | -1.35 | 2.35E-02 | 7.38E-01 |
| HSD3B1 | mRNA | -1.34 | 2.36E-02 | 7.38E-01 |
| GGT2 | mRNA | -1.34 | 4.70E-02 | 7.38E-01 |
| ARMCX6 | mRNA | -1.32 | 1.85E-03 | 7.38E-01 |
| MAGEA3 | mRNA | -1.32 | 5.65E-03 | 7.38E-01 |
| PRR16 | mRNA | -1.32 | 6.00E-02 | 7.38E-01 |
| BEST4 | mRNA | -1.31 | 6.55E-02 | 7.38E-01 |
| ATP6V1B1 | mRNA | -1.31 | 2.20E-03 | 7.38E-01 |
| RASGRP1 | mRNA | -1.31 | 8.27E-02 | 7.38E-01 |
| SNX20 | mRNA | -1.31 | 6.46E-02 | 7.38E-01 |
| PLSCR4 | mRNA | -1.30 | 9.54E-02 | 7.42E-01 |
| CALD1 | mRNA | -1.30 | 1.41E-03 | 7.38E-01 |
| TCEAL8 | mRNA | -1.30 | 4.28E-03 | 7.38E-01 |
| CCDC80 | mRNA | -1.30 | 1.54E-02 | 7.38E-01 |
| STON1 | mRNA | -1.28 | 1.08E-02 | 7.38E-01 |
| FBLN5 | mRNA | -1.28 | 7.40E-02 | 7.38E-01 |
| DCST1 | mRNA | -1.28 | 4.08E-02 | 7.38E-01 |
| AOC2 | mRNA | -1.27 | 7.34E-02 | 7.38E-01 |
| RASA4B | mRNA | -1.27 | 6.95E-03 | 7.38E-01 |
| SLC7A7 | mRNA | -1.26 | 1.62E-02 | 7.38E-01 |
| MAP2 | mRNA | -1.26 | 1.40E-02 | 7.38E-01 |
| BVES | mRNA | -1.26 | 3.48E-02 | 7.38E-01 |
| NR5A2 | mRNA | -1.26 | 1.25E-02 | 7.38E-01 |
| SLC35G2 | mRNA | -1.26 | 1.15E-02 | 7.38E-01 |
| MN1 | mRNA | -1.25 | 4.69E-02 | 7.38E-01 |
| MT1X | mRNA | -1.25 | 1.01E-03 | 7.38E-01 |
| GOLGA8T | mRNA | -1.24 | 7.24E-02 | 7.38E-01 |
| HOXA2 | mRNA | -1.23 | 9.37E-02 | 7.42E-01 |
| MAPK4 | mRNA | -1.23 | 8.32E-02 | 7.38E-01 |
| CABCOCO1 | mRNA | -1.23 | 7.87E-02 | 7.38E-01 |
| CPLANE1 | mRNA | -1.22 | 4.47E-03 | 7.38E-01 |
| SATB1 | mRNA | -1.22 | 2.59E-03 | 7.38E-01 |
| RAB40A | mRNA | -1.22 | 9.43E-02 | 7.42E-01 |
| BIRC7 | mRNA | -1.22 | 3.84E-02 | 7.38E-01 |
| GSDME | mRNA | -1.22 | 8.64E-03 | 7.38E-01 |
| ADAM28 | mRNA | -1.22 | 6.60E-02 | 7.38E-01 |
| BCO2 | mRNA | -1.21 | 2.12E-02 | 7.38E-01 |
| JAZF1 | mRNA | -1.20 | 1.26E-02 | 7.38E-01 |
| NFE2 | mRNA | -1.20 | 5.08E-02 | 7.38E-01 |
| CYFIP2 | mRNA | -1.20 | 6.15E-02 | 7.38E-01 |
| HIGD1C | mRNA | -1.20 | 7.70E-02 | 7.38E-01 |
| ADGRA2 | mRNA | -1.19 | 7.86E-02 | 7.38E-01 |
| YPEL4 | mRNA | -1.19 | 4.10E-02 | 7.38E-01 |
| KLHDC1 | mRNA | -1.19 | 3.13E-02 | 7.38E-01 |
| CYP1B1 | mRNA | -1.19 | 8.84E-02 | 7.42E-01 |
| NPTXR | mRNA | -1.18 | 5.07E-02 | 7.38E-01 |
| ARHGDIG | mRNA | -1.18 | 8.82E-02 | 7.42E-01 |
| YPEL1 | mRNA | -1.18 | 2.22E-02 | 7.38E-01 |
| C2 | mRNA | -1.17 | 5.93E-02 | 7.38E-01 |
| TXNIP | mRNA | -1.17 | 3.12E-02 | 7.38E-01 |
| SLC5A10 | mRNA | -1.17 | 8.29E-02 | 7.38E-01 |
| ZDHHC11 | mRNA | -1.17 | 2.46E-02 | 7.38E-01 |
| ADAMTS7 | mRNA | -1.16 | 5.79E-02 | 7.38E-01 |
| TP63 | mRNA | -1.16 | 4.78E-02 | 7.38E-01 |
| ZBTB10 | mRNA | -1.16 | 2.65E-02 | 7.38E-01 |
| TNC | mRNA | -1.15 | 1.47E-02 | 7.38E-01 |
| SQLE | mRNA | -1.15 | 8.54E-02 | 7.40E-01 |
| PID1 | mRNA | -1.15 | 5.31E-02 | 7.38E-01 |
| CRISP3 | mRNA | -1.15 | 8.54E-02 | 7.40E-01 |
| HEG1 | mRNA | -1.14 | 1.18E-02 | 7.38E-01 |
| KLK7 | mRNA | -1.13 | 2.41E-02 | 7.38E-01 |
| ALDOC | mRNA | -1.13 | 8.88E-03 | 7.38E-01 |
| AKAP12 | mRNA | -1.12 | 2.10E-02 | 7.38E-01 |
| NEO1 | mRNA | -1.12 | 1.76E-02 | 7.38E-01 |
| AXL | mRNA | -1.12 | 1.00E-01 | 7.42E-01 |
| ALDH2 | mRNA | -1.12 | 6.21E-02 | 7.38E-01 |
| PLAG1 | mRNA | -1.12 | 1.65E-02 | 7.38E-01 |
| VWDE | mRNA | -1.11 | 3.02E-02 | 7.38E-01 |
| NR2F1 | mRNA | -1.11 | 5.05E-02 | 7.38E-01 |
| LOX | mRNA | -1.11 | 4.61E-02 | 7.38E-01 |
| SLC22A31 | mRNA | -1.11 | 2.46E-02 | 7.38E-01 |
| HAPLN3 | mRNA | -1.11 | 2.10E-02 | 7.38E-01 |
| SMARCD3 | mRNA | -1.11 | 3.79E-02 | 7.38E-01 |
| AHRR | mRNA | -1.11 | 5.08E-03 | 7.38E-01 |
| AK7 | mRNA | -1.10 | 2.58E-02 | 7.38E-01 |
| VWA5B2 | mRNA | -1.10 | 5.19E-02 | 7.38E-01 |
| SH3BGRL | mRNA | -1.10 | 2.05E-03 | 7.38E-01 |
| LDHC | mRNA | -1.10 | 5.59E-02 | 7.38E-01 |
| SACS | mRNA | -1.09 | 6.92E-02 | 7.38E-01 |
| ANK2 | mRNA | -1.09 | 6.44E-02 | 7.38E-01 |
| PRR5L | mRNA | -1.09 | 2.10E-02 | 7.38E-01 |
| TMEM231 | mRNA | -1.09 | 1.97E-02 | 7.38E-01 |
| JAK2 | mRNA | -1.09 | 2.38E-02 | 7.38E-01 |
| ADAM19 | mRNA | -1.09 | 3.76E-02 | 7.38E-01 |
| TMPRSS9 | mRNA | -1.09 | 9.30E-02 | 7.42E-01 |
| CCDC169 | mRNA | -1.08 | 9.38E-02 | 7.42E-01 |
| PDGFD | mRNA | -1.08 | 1.65E-02 | 7.38E-01 |
| SCUBE3 | mRNA | -1.08 | 3.03E-02 | 7.38E-01 |
| LCP1 | mRNA | -1.08 | 6.30E-04 | 7.38E-01 |
| MSMO1 | mRNA | -1.08 | 5.56E-02 | 7.38E-01 |
| GRIK2 | mRNA | -1.08 | 7.27E-02 | 7.38E-01 |
| RASSF9 | mRNA | -1.07 | 5.99E-02 | 7.38E-01 |
| PGM2L1 | mRNA | -1.07 | 7.78E-03 | 7.38E-01 |
| FGFR4 | mRNA | -1.07 | 2.64E-02 | 7.38E-01 |
| ST8SIA4 | mRNA | -1.06 | 1.47E-02 | 7.38E-01 |
| WLS | mRNA | -1.06 | 3.07E-02 | 7.38E-01 |
| MPIG6B | mRNA | -1.06 | 4.33E-02 | 7.38E-01 |
| ENPP1 | mRNA | -1.06 | 2.25E-02 | 7.38E-01 |
| ARHGEF37 | mRNA | -1.06 | 2.63E-02 | 7.38E-01 |
| CRYAB | mRNA | -1.06 | 5.93E-02 | 7.38E-01 |
| FOXP2 | mRNA | -1.05 | 5.77E-02 | 7.38E-01 |
| ARID3C | mRNA | -1.04 | 5.01E-02 | 7.38E-01 |
| CDKN1C | mRNA | -1.04 | 3.49E-02 | 7.38E-01 |
| MCF2L2 | mRNA | -1.04 | 1.91E-02 | 7.38E-01 |
| POU6F1 | mRNA | -1.04 | 3.17E-02 | 7.38E-01 |
| IFFO1 | mRNA | -1.04 | 5.98E-02 | 7.38E-01 |
| CAPN5 | mRNA | -1.04 | 8.39E-02 | 7.38E-01 |
| SLC45A1 | mRNA | -1.03 | 3.23E-02 | 7.38E-01 |
| MAP1B | mRNA | -1.03 | 5.65E-02 | 7.38E-01 |
| EPB41L2 | mRNA | -1.03 | 2.03E-03 | 7.38E-01 |
| S100A1 | mRNA | -1.03 | 6.85E-02 | 7.38E-01 |
| MMP13 | mRNA | -1.03 | 2.65E-02 | 7.38E-01 |
| HES2 | mRNA | -1.02 | 3.72E-02 | 7.38E-01 |
| EIF4E3 | mRNA | -1.02 | 9.94E-02 | 7.42E-01 |
| FILIP1L | mRNA | -1.02 | 6.06E-03 | 7.38E-01 |
| TSPYL5 | mRNA | -1.02 | 4.64E-02 | 7.38E-01 |
| CLCNKA | mRNA | -1.02 | 8.39E-02 | 7.38E-01 |
| RETREG1 | mRNA | -1.02 | 8.95E-03 | 7.38E-01 |
| GAS6 | mRNA | -1.01 | 2.43E-02 | 7.38E-01 |
| AQP1 | mRNA | -1.01 | 3.84E-02 | 7.38E-01 |
| CGB7 | mRNA | -1.01 | 2.35E-02 | 7.38E-01 |
| GPR37 | mRNA | -1.00 | 2.36E-02 | 7.38E-01 |
| OXCT1 | mRNA | -1.00 | 4.02E-02 | 7.38E-01 |
| ACSL4 | mRNA | -0.99 | 1.17E-02 | 7.38E-01 |
| CD24 | mRNA | -0.99 | 7.73E-04 | 7.38E-01 |
| SLC7A8 | mRNA | -0.99 | 2.03E-02 | 7.38E-01 |
| GLIS1 | mRNA | -0.99 | 5.99E-02 | 7.38E-01 |
| MROH7 | mRNA | -0.99 | 8.47E-02 | 7.38E-01 |
| AGPAT4 | mRNA | -0.98 | 2.82E-02 | 7.38E-01 |
| FOXG1 | mRNA | -0.98 | 5.70E-02 | 7.38E-01 |
| MME | mRNA | -0.98 | 4.65E-02 | 7.38E-01 |
| SCARF2 | mRNA | -0.98 | 4.05E-02 | 7.38E-01 |
| LIMS2 | mRNA | -0.98 | 7.38E-02 | 7.38E-01 |
| OGDHL | mRNA | -0.97 | 6.62E-02 | 7.38E-01 |
| SYT1 | mRNA | -0.97 | 6.22E-02 | 7.38E-01 |
| LY6G6C | mRNA | -0.97 | 2.03E-02 | 7.38E-01 |
| FRAS1 | mRNA | -0.97 | 1.59E-02 | 7.38E-01 |
| TVP23C-CDRT4 | mRNA | -0.96 | 3.96E-02 | 7.38E-01 |
| NFATC4 | mRNA | -0.96 | 5.74E-03 | 7.38E-01 |
| BOC | mRNA | -0.96 | 3.72E-02 | 7.38E-01 |
| KRT81 | mRNA | -0.96 | 9.36E-02 | 7.42E-01 |
| ATP8B3 | mRNA | -0.96 | 9.00E-02 | 7.42E-01 |
| SCIN | mRNA | -0.95 | 4.93E-02 | 7.38E-01 |
| DNHD1 | mRNA | -0.95 | 5.42E-03 | 7.38E-01 |
| MYO15B | mRNA | -0.95 | 1.43E-02 | 7.38E-01 |
| DENND2C | mRNA | -0.95 | 5.58E-02 | 7.38E-01 |
| PTPRR | mRNA | -0.94 | 3.27E-02 | 7.38E-01 |
| FHL3 | mRNA | -0.94 | 1.74E-02 | 7.38E-01 |
| ST3GAL6 | mRNA | -0.94 | 7.80E-02 | 7.38E-01 |
| NRCAM | mRNA | -0.93 | 3.39E-03 | 7.38E-01 |
| SNPH | mRNA | -0.93 | 4.73E-02 | 7.38E-01 |
| TMOD2 | mRNA | -0.93 | 7.04E-02 | 7.38E-01 |
| PMP22 | mRNA | -0.93 | 5.49E-02 | 7.38E-01 |
| NBPF10 | mRNA | -0.93 | 1.46E-02 | 7.38E-01 |
| NEDD9 | mRNA | -0.93 | 5.47E-02 | 7.38E-01 |
| LSP1 | mRNA | -0.92 | 7.53E-02 | 7.38E-01 |
| TAGLN | mRNA | -0.92 | 3.31E-02 | 7.38E-01 |
| PRSS12 | mRNA | -0.92 | 2.54E-02 | 7.38E-01 |
| SLC2A12 | mRNA | -0.92 | 6.51E-02 | 7.38E-01 |
| ADARB1 | mRNA | -0.92 | 1.02E-02 | 7.38E-01 |
| PLD1 | mRNA | -0.91 | 3.23E-02 | 7.38E-01 |
| TUBA1A | mRNA | -0.91 | 2.99E-02 | 7.38E-01 |
| EBF4 | mRNA | -0.91 | 8.98E-02 | 7.42E-01 |
| KCNG1 | mRNA | -0.91 | 4.30E-02 | 7.38E-01 |
| CCDC33 | mRNA | -0.91 | 9.65E-02 | 7.42E-01 |
| PRSS16 | mRNA | -0.91 | 5.35E-02 | 7.38E-01 |
| PTGS1 | mRNA | -0.90 | 3.12E-02 | 7.38E-01 |
| RRAGD | mRNA | -0.90 | 7.87E-02 | 7.38E-01 |
| PRR29 | mRNA | -0.90 | 7.89E-02 | 7.38E-01 |
| TNS1 | mRNA | -0.89 | 3.91E-03 | 7.38E-01 |
| CATSPERG | mRNA | -0.89 | 6.94E-02 | 7.38E-01 |
| CD109 | mRNA | -0.89 | 2.60E-02 | 7.38E-01 |
| CTAGE6 | mRNA | -0.89 | 7.46E-02 | 7.38E-01 |
| DUSP13 | mRNA | -0.88 | 5.98E-03 | 7.38E-01 |
| RICTOR | mRNA | -0.88 | 2.18E-03 | 7.38E-01 |
| KLHL29 | mRNA | -0.87 | 7.63E-02 | 7.38E-01 |
| LRRCC1 | mRNA | -0.87 | 7.75E-02 | 7.38E-01 |
| CITED2 | mRNA | -0.86 | 6.72E-03 | 7.38E-01 |
| SH3PXD2A | mRNA | -0.86 | 3.36E-03 | 7.38E-01 |
| LY75 | mRNA | -0.86 | 6.07E-02 | 7.38E-01 |
| CD274 | mRNA | -0.86 | 8.03E-02 | 7.38E-01 |
| TMPRSS6 | mRNA | -0.86 | 2.78E-02 | 7.38E-01 |
| FAM126A | mRNA | -0.86 | 2.03E-02 | 7.38E-01 |
| ACSS2 | mRNA | -0.86 | 2.00E-02 | 7.38E-01 |
| PCYOX1L | mRNA | -0.86 | 6.06E-02 | 7.38E-01 |
| DSE | mRNA | -0.85 | 5.55E-02 | 7.38E-01 |
| KLK5 | mRNA | -0.85 | 7.74E-03 | 7.38E-01 |
| FAHD2B | mRNA | -0.85 | 5.94E-02 | 7.38E-01 |
| SIMC1 | mRNA | -0.85 | 4.18E-02 | 7.38E-01 |
| KLF8 | mRNA | -0.85 | 2.60E-02 | 7.38E-01 |
| ESPN | mRNA | -0.85 | 6.63E-02 | 7.38E-01 |
| NXNL2 | mRNA | -0.85 | 9.50E-02 | 7.42E-01 |
| SNED1 | mRNA | -0.85 | 4.73E-02 | 7.38E-01 |
| MICU3 | mRNA | -0.84 | 4.18E-02 | 7.38E-01 |
| KRBA1 | mRNA | -0.84 | 6.92E-02 | 7.38E-01 |
| LRP1 | mRNA | -0.84 | 4.50E-02 | 7.38E-01 |
| SLC2A10 | mRNA | -0.84 | 9.06E-02 | 7.42E-01 |
| LOXL2 | mRNA | -0.84 | 5.11E-02 | 7.38E-01 |
| P3H2 | mRNA | -0.84 | 4.34E-02 | 7.38E-01 |
| GGT5 | mRNA | -0.84 | 4.05E-02 | 7.38E-01 |
| NOD2 | mRNA | -0.83 | 7.58E-02 | 7.38E-01 |
| LRRC6 | mRNA | -0.83 | 3.40E-02 | 7.38E-01 |
| TMEM106B | mRNA | -0.83 | 3.58E-03 | 7.38E-01 |
| SLC23A2 | mRNA | -0.83 | 5.48E-02 | 7.38E-01 |
| NINL | mRNA | -0.83 | 1.10E-02 | 7.38E-01 |
| COL6A1 | mRNA | -0.82 | 2.70E-02 | 7.38E-01 |
| BSN | mRNA | -0.82 | 7.72E-02 | 7.38E-01 |
| DLX4 | mRNA | -0.82 | 5.95E-02 | 7.38E-01 |
| BMF | mRNA | -0.82 | 2.72E-02 | 7.38E-01 |
| HECA | mRNA | -0.82 | 2.04E-02 | 7.38E-01 |
| ARHGEF25 | mRNA | -0.81 | 1.73E-02 | 7.38E-01 |
| GABARAPL1 | mRNA | -0.81 | 7.20E-02 | 7.38E-01 |
| F2RL2 | mRNA | -0.81 | 8.55E-02 | 7.40E-01 |
| IL18 | mRNA | -0.80 | 8.17E-02 | 7.38E-01 |
| PARP8 | mRNA | -0.80 | 4.15E-02 | 7.38E-01 |
| MYCL | mRNA | -0.80 | 4.83E-02 | 7.38E-01 |
| PCDHAC2 | mRNA | -0.80 | 3.02E-02 | 7.38E-01 |
| ARHGAP4 | mRNA | -0.79 | 3.19E-02 | 7.38E-01 |
| GLCCI1 | mRNA | -0.79 | 9.35E-03 | 7.38E-01 |
| FREM2 | mRNA | -0.79 | 5.01E-02 | 7.38E-01 |
| TMEM52B | mRNA | -0.79 | 7.91E-02 | 7.38E-01 |
| ITPR2 | mRNA | -0.79 | 1.33E-02 | 7.38E-01 |
| SPEG | mRNA | -0.79 | 1.41E-02 | 7.38E-01 |
| ILDR1 | mRNA | -0.78 | 3.54E-02 | 7.38E-01 |
| DPYD | mRNA | -0.78 | 5.83E-02 | 7.38E-01 |
| PTPRM | mRNA | -0.78 | 2.55E-02 | 7.38E-01 |
| VANGL2 | mRNA | -0.78 | 1.16E-02 | 7.38E-01 |
| LRRN4 | mRNA | -0.78 | 5.90E-02 | 7.38E-01 |
| TPX2 | mRNA | -0.78 | 8.19E-02 | 7.38E-01 |
| CASTOR1 | mRNA | -0.78 | 8.03E-02 | 7.38E-01 |
| FZD7 | mRNA | -0.78 | 2.22E-02 | 7.38E-01 |
| PREP | mRNA | -0.78 | 1.97E-02 | 7.38E-01 |
| NPHP3 | mRNA | -0.77 | 1.00E-02 | 7.38E-01 |
| ANTXR2 | mRNA | -0.77 | 6.37E-02 | 7.38E-01 |
| THBS4 | mRNA | -0.77 | 4.20E-02 | 7.38E-01 |
| BACE1 | mRNA | -0.77 | 1.90E-02 | 7.38E-01 |
| FAR2 | mRNA | -0.77 | 2.18E-02 | 7.38E-01 |
| GABBR1 | mRNA | -0.77 | 5.90E-02 | 7.38E-01 |
| TTC28 | mRNA | -0.77 | 3.90E-02 | 7.38E-01 |
| PREX1 | mRNA | -0.77 | 5.64E-02 | 7.38E-01 |
| SAMD4A | mRNA | -0.77 | 3.89E-02 | 7.38E-01 |
| TNXB | mRNA | -0.77 | 2.10E-02 | 7.38E-01 |
| RRAS | mRNA | -0.76 | 7.94E-02 | 7.38E-01 |
| STK33 | mRNA | -0.76 | 9.65E-02 | 7.42E-01 |
| ASGR1 | mRNA | -0.76 | 8.41E-02 | 7.38E-01 |
| BHLHE40 | mRNA | -0.76 | 4.82E-02 | 7.38E-01 |
| PRICKLE1 | mRNA | -0.76 | 9.57E-02 | 7.42E-01 |
| PTPRG | mRNA | -0.76 | 9.91E-02 | 7.42E-01 |
| FAM78A | mRNA | -0.76 | 3.87E-02 | 7.38E-01 |
| FHL2 | mRNA | -0.75 | 7.37E-02 | 7.38E-01 |
| TSSK3 | mRNA | -0.75 | 5.96E-02 | 7.38E-01 |
| CACNA1H | mRNA | -0.75 | 7.79E-02 | 7.38E-01 |
| CKB | mRNA | -0.75 | 7.02E-02 | 7.38E-01 |
| L1CAM | mRNA | -0.74 | 4.57E-02 | 7.38E-01 |
| SLC16A2 | mRNA | -0.74 | 4.04E-02 | 7.38E-01 |
| RAPGEF5 | mRNA | -0.74 | 9.21E-02 | 7.42E-01 |
| RHBDL1 | mRNA | -0.74 | 9.30E-02 | 7.42E-01 |
| ATP2B4 | mRNA | -0.74 | 2.16E-02 | 7.38E-01 |
| REPS2 | mRNA | -0.74 | 4.69E-02 | 7.38E-01 |
| LYNX1 | mRNA | -0.74 | 6.18E-02 | 7.38E-01 |
| GPRIN2 | mRNA | -0.74 | 2.95E-02 | 7.38E-01 |
| NAV2 | mRNA | -0.74 | 2.62E-02 | 7.38E-01 |
| PNRC1 | mRNA | -0.73 | 2.01E-02 | 7.38E-01 |
| CDON | mRNA | -0.73 | 1.04E-02 | 7.38E-01 |
| ZMAT3 | mRNA | -0.73 | 6.61E-03 | 7.38E-01 |
| JPH2 | mRNA | -0.73 | 4.39E-02 | 7.38E-01 |
| NECTIN3 | mRNA | -0.73 | 4.47E-02 | 7.38E-01 |
| SARDH | mRNA | -0.73 | 9.19E-02 | 7.42E-01 |
| GPSM1 | mRNA | -0.73 | 4.06E-02 | 7.38E-01 |
| TENT5A | mRNA | -0.72 | 3.35E-02 | 7.38E-01 |
| C20orf194 | mRNA | -0.72 | 3.48E-02 | 7.38E-01 |
| SLC25A30 | mRNA | -0.72 | 3.62E-02 | 7.38E-01 |
| IGSF9 | mRNA | -0.72 | 9.58E-03 | 7.38E-01 |
| CREBRF | mRNA | -0.72 | 8.29E-03 | 7.38E-01 |
| MLLT11 | mRNA | -0.72 | 6.87E-02 | 7.38E-01 |
| RAB32 | mRNA | -0.72 | 4.41E-02 | 7.38E-01 |
| PAQR5 | mRNA | -0.72 | 8.23E-02 | 7.38E-01 |
| PPIC | mRNA | -0.71 | 7.26E-03 | 7.38E-01 |
| HERC5 | mRNA | -0.71 | 2.16E-02 | 7.38E-01 |
| RASGEF1A | mRNA | -0.71 | 2.02E-02 | 7.38E-01 |
| ZFYVE9 | mRNA | -0.71 | 1.52E-02 | 7.38E-01 |
| KLHL24 | mRNA | -0.71 | 6.23E-02 | 7.38E-01 |
| IRF6 | mRNA | -0.71 | 2.16E-02 | 7.38E-01 |
| HIVEP2 | mRNA | -0.71 | 2.05E-02 | 7.38E-01 |
| MICAL1 | mRNA | -0.71 | 1.11E-02 | 7.38E-01 |
| AHI1 | mRNA | -0.71 | 6.33E-02 | 7.38E-01 |
| STK38L | mRNA | -0.70 | 9.19E-03 | 7.38E-01 |
| TP53INP2 | mRNA | -0.70 | 7.33E-02 | 7.38E-01 |
| RASA4 | mRNA | -0.70 | 8.23E-02 | 7.38E-01 |
| MAP3K12 | mRNA | -0.70 | 8.46E-02 | 7.38E-01 |
| GNG4 | mRNA | -0.70 | 8.61E-02 | 7.41E-01 |
| CLTCL1 | mRNA | -0.70 | 6.27E-02 | 7.38E-01 |
| PER1 | mRNA | -0.70 | 7.97E-03 | 7.38E-01 |
| ATP6V0A4 | mRNA | -0.69 | 8.33E-02 | 7.38E-01 |
| SYT15 | mRNA | -0.69 | 3.10E-02 | 7.38E-01 |
| GRAMD1C | mRNA | -0.69 | 5.18E-02 | 7.38E-01 |
| NMNAT2 | mRNA | -0.69 | 2.39E-02 | 7.38E-01 |
| MID2 | mRNA | -0.69 | 7.17E-02 | 7.38E-01 |
| ECHDC2 | mRNA | -0.69 | 2.13E-02 | 7.38E-01 |
| THSD4 | mRNA | -0.69 | 7.56E-02 | 7.38E-01 |
| FUT4 | mRNA | -0.68 | 8.97E-02 | 7.42E-01 |
| MC1R | mRNA | -0.68 | 7.69E-02 | 7.38E-01 |
| BDNF | mRNA | -0.68 | 6.22E-02 | 7.38E-01 |
| KLHL5 | mRNA | -0.68 | 9.28E-02 | 7.42E-01 |
| PRDM1 | mRNA | -0.68 | 7.83E-02 | 7.38E-01 |
| SEMA3A | mRNA | -0.68 | 9.24E-02 | 7.42E-01 |
| WNT6 | mRNA | -0.68 | 3.03E-02 | 7.38E-01 |
| NATD1 | mRNA | -0.67 | 6.71E-02 | 7.38E-01 |
| CACNG8 | mRNA | -0.67 | 3.21E-02 | 7.38E-01 |
| DOK4 | mRNA | -0.67 | 2.18E-02 | 7.38E-01 |
| MCAM | mRNA | -0.67 | 7.30E-02 | 7.38E-01 |
| BEND7 | mRNA | -0.67 | 2.40E-02 | 7.38E-01 |
| PDGFC | mRNA | -0.66 | 1.81E-02 | 7.38E-01 |
| CTNNAL1 | mRNA | -0.66 | 7.83E-02 | 7.38E-01 |
| CEMIP2 | mRNA | -0.66 | 1.97E-02 | 7.38E-01 |
| MMP11 | mRNA | -0.66 | 9.08E-02 | 7.42E-01 |
| DAPK1 | mRNA | -0.66 | 1.37E-02 | 7.38E-01 |
| TTC33 | mRNA | -0.66 | 1.97E-02 | 7.38E-01 |
| CITED4 | mRNA | -0.66 | 9.65E-02 | 7.42E-01 |
| DENND5B | mRNA | -0.66 | 5.84E-02 | 7.38E-01 |
| OBSCN | mRNA | -0.66 | 1.17E-02 | 7.38E-01 |
| MFGE8 | mRNA | -0.65 | 4.78E-03 | 7.38E-01 |
| FGD4 | mRNA | -0.65 | 2.79E-02 | 7.38E-01 |
| CCBE1 | mRNA | -0.65 | 3.65E-02 | 7.38E-01 |
| BNIPL | mRNA | -0.65 | 8.20E-02 | 7.38E-01 |
| GGT7 | mRNA | -0.65 | 3.73E-02 | 7.38E-01 |
| NAB2 | mRNA | -0.65 | 1.36E-02 | 7.38E-01 |
| AK3 | mRNA | -0.65 | 3.64E-02 | 7.38E-01 |
| JMY | mRNA | -0.65 | 2.64E-02 | 7.38E-01 |
| CYTH3 | mRNA | -0.65 | 3.26E-02 | 7.38E-01 |
| GLS2 | mRNA | -0.65 | 7.63E-02 | 7.38E-01 |
| KIAA0895 | mRNA | -0.65 | 3.56E-02 | 7.38E-01 |
| SLC4A3 | mRNA | -0.65 | 3.95E-02 | 7.38E-01 |
| SSBP2 | mRNA | -0.64 | 3.62E-02 | 7.38E-01 |
| ARRDC3 | mRNA | -0.64 | 1.82E-02 | 7.38E-01 |
| RGL1 | mRNA | -0.64 | 2.59E-02 | 7.38E-01 |
| RAVER2 | mRNA | -0.64 | 6.23E-02 | 7.38E-01 |
| MT2A | mRNA | -0.64 | 9.17E-02 | 7.42E-01 |
| ZNF581 | mRNA | -0.64 | 1.37E-02 | 7.38E-01 |
| ACVR2B | mRNA | -0.64 | 4.31E-02 | 7.38E-01 |
| SPATA7 | mRNA | -0.64 | 8.29E-02 | 7.38E-01 |
| UBE2Q2 | mRNA | -0.64 | 2.97E-02 | 7.38E-01 |
| MYO6 | mRNA | -0.64 | 3.23E-02 | 7.38E-01 |
| ZNF277 | mRNA | -0.63 | 1.10E-02 | 7.38E-01 |
| RIC1 | mRNA | -0.63 | 2.35E-02 | 7.38E-01 |
| ZNF385A | mRNA | -0.63 | 4.93E-02 | 7.38E-01 |
| TTC7B | mRNA | -0.63 | 1.98E-02 | 7.38E-01 |
| GRAMD1A | mRNA | -0.63 | 6.50E-03 | 7.38E-01 |
| ACAD11 | mRNA | -0.63 | 3.16E-02 | 7.38E-01 |
| ULK2 | mRNA | -0.63 | 6.67E-02 | 7.38E-01 |
| FAM8A1 | mRNA | -0.63 | 1.56E-02 | 7.38E-01 |
| LAMB1 | mRNA | -0.63 | 8.23E-02 | 7.38E-01 |
| NR1D1 | mRNA | -0.63 | 3.89E-02 | 7.38E-01 |
| MIB1 | mRNA | -0.63 | 5.01E-02 | 7.38E-01 |
| CLUAP1 | mRNA | -0.63 | 4.68E-02 | 7.38E-01 |
| BMT2 | mRNA | -0.63 | 6.78E-02 | 7.38E-01 |
| HIBADH | mRNA | -0.63 | 1.53E-02 | 7.38E-01 |
| CHST3 | mRNA | -0.63 | 9.66E-02 | 7.42E-01 |
| CDK14 | mRNA | -0.63 | 6.76E-02 | 7.38E-01 |
| FBXL4 | mRNA | -0.62 | 7.24E-02 | 7.38E-01 |
| PAPSS2 | mRNA | -0.62 | 5.24E-02 | 7.38E-01 |
| AGO4 | mRNA | -0.62 | 2.77E-02 | 7.38E-01 |
| EFEMP1 | mRNA | -0.62 | 3.47E-02 | 7.38E-01 |
| CELSR2 | mRNA | -0.62 | 3.27E-02 | 7.38E-01 |
| STK17B | mRNA | -0.62 | 6.35E-02 | 7.38E-01 |
| VWA7 | mRNA | -0.62 | 8.20E-02 | 7.38E-01 |
| CTBS | mRNA | -0.62 | 8.48E-02 | 7.38E-01 |
| UACA | mRNA | -0.61 | 7.37E-02 | 7.38E-01 |
| SATB2 | mRNA | -0.61 | 6.08E-02 | 7.38E-01 |
| CRIM1 | mRNA | -0.61 | 4.00E-02 | 7.38E-01 |
| PPP2R3A | mRNA | -0.61 | 3.01E-02 | 7.38E-01 |
| CALCOCO1 | mRNA | -0.61 | 1.86E-02 | 7.38E-01 |
| USP40 | mRNA | -0.61 | 1.79E-02 | 7.38E-01 |
| CCNG1 | mRNA | -0.61 | 1.02E-02 | 7.38E-01 |
| GRAMD2B | mRNA | -0.61 | 2.75E-02 | 7.38E-01 |
| CRYBG1 | mRNA | -0.61 | 3.43E-02 | 7.38E-01 |
| MAPK8IP1 | mRNA | -0.61 | 2.82E-02 | 7.38E-01 |
| DISC1 | mRNA | -0.61 | 3.90E-02 | 7.38E-01 |
| ADGRL2 | mRNA | -0.61 | 5.37E-02 | 7.38E-01 |
| AMOTL2 | mRNA | -0.60 | 8.29E-02 | 7.38E-01 |
| TBC1D1 | mRNA | -0.60 | 6.10E-02 | 7.38E-01 |
| TUT4 | mRNA | -0.60 | 8.16E-03 | 7.38E-01 |
| PHF20 | mRNA | -0.60 | 5.51E-02 | 7.38E-01 |
| PTPN13 | mRNA | -0.60 | 1.47E-02 | 7.38E-01 |
| PCNX2 | mRNA | -0.60 | 4.10E-02 | 7.38E-01 |
| OGFRL1 | mRNA | -0.60 | 6.47E-02 | 7.38E-01 |
| NDRG1 | mRNA | -0.60 | 2.87E-02 | 7.38E-01 |
| CPAMD8 | mRNA | -0.60 | 5.38E-02 | 7.38E-01 |
| ARHGEF40 | mRNA | -0.60 | 6.23E-02 | 7.38E-01 |
| ASIC1 | mRNA | -0.60 | 2.00E-02 | 7.38E-01 |
| RNF19A | mRNA | -0.60 | 1.26E-02 | 7.38E-01 |
| EIF4A2 | mRNA | -0.59 | 7.72E-02 | 7.38E-01 |
| ITGB8 | mRNA | -0.59 | 5.68E-02 | 7.38E-01 |
| MFAP3L | mRNA | -0.59 | 5.39E-02 | 7.38E-01 |
| LZTS3 | mRNA | -0.59 | 4.74E-02 | 7.38E-01 |
| ANKH | mRNA | -0.59 | 7.07E-02 | 7.38E-01 |
| NOTCH2NLC | mRNA | -0.59 | 8.53E-02 | 7.40E-01 |
| CCDC68 | mRNA | -0.59 | 9.32E-02 | 7.42E-01 |
| CERCAM | mRNA | -0.59 | 1.47E-02 | 7.38E-01 |
| SPTB | mRNA | -0.59 | 3.85E-02 | 7.38E-01 |
| NOTCH2 | mRNA | -0.59 | 2.46E-02 | 7.38E-01 |
| GRAMD1B | mRNA | -0.59 | 8.45E-02 | 7.38E-01 |
| MAPK15 | mRNA | -0.59 | 9.89E-02 | 7.42E-01 |
| ANTXR1 | mRNA | -0.59 | 5.70E-02 | 7.38E-01 |
| THBS3 | mRNA | -0.58 | 5.33E-02 | 7.38E-01 |
| NOTCH2NLA | mRNA | -0.58 | 8.36E-02 | 7.38E-01 |
| SLC17A5 | mRNA | -0.58 | 2.57E-02 | 7.38E-01 |
| MARCKS | mRNA | -0.58 | 3.93E-02 | 7.38E-01 |
| FOSL2 | mRNA | -0.58 | 2.42E-02 | 7.38E-01 |
| TMEM41B | mRNA | -0.58 | 8.84E-03 | 7.38E-01 |
| FAM229B | mRNA | -0.58 | 7.43E-02 | 7.38E-01 |
| RIMKLB | mRNA | -0.58 | 3.96E-02 | 7.38E-01 |
| HINT3 | mRNA | -0.57 | 3.32E-02 | 7.38E-01 |
| TIPARP | mRNA | -0.57 | 6.70E-02 | 7.38E-01 |
| TGFBR1 | mRNA | -0.57 | 3.66E-02 | 7.38E-01 |
| MYH10 | mRNA | -0.57 | 6.46E-02 | 7.38E-01 |
| PDE4DIP | mRNA | -0.57 | 2.67E-02 | 7.38E-01 |
| TSPAN9 | mRNA | -0.57 | 4.09E-02 | 7.38E-01 |
| USP49 | mRNA | -0.57 | 3.70E-02 | 7.38E-01 |
| CCDC113 | mRNA | -0.57 | 4.84E-02 | 7.38E-01 |
| DHX40 | mRNA | -0.57 | 1.24E-02 | 7.38E-01 |
| RAB3B | mRNA | -0.57 | 8.68E-02 | 7.42E-01 |
| RASA1 | mRNA | -0.57 | 4.31E-02 | 7.38E-01 |
| E2F5 | mRNA | -0.57 | 3.68E-02 | 7.38E-01 |
| ARHGAP42 | mRNA | -0.57 | 7.61E-02 | 7.38E-01 |
| CCNB1IP1 | mRNA | -0.57 | 3.13E-02 | 7.38E-01 |
| EYA4 | mRNA | -0.57 | 8.31E-02 | 7.38E-01 |
| STX7 | mRNA | -0.57 | 1.99E-02 | 7.38E-01 |
| CES3 | mRNA | -0.57 | 5.38E-02 | 7.38E-01 |
| CCDC28A | mRNA | -0.57 | 8.65E-02 | 7.42E-01 |
| MAGEE1 | mRNA | -0.56 | 8.08E-02 | 7.38E-01 |
| PPP1R2 | mRNA | -0.56 | 1.07E-02 | 7.38E-01 |
| TFPI | mRNA | -0.56 | 5.68E-02 | 7.38E-01 |
| SIDT2 | mRNA | -0.56 | 2.05E-02 | 7.38E-01 |
| PAM | mRNA | -0.56 | 6.11E-02 | 7.38E-01 |
| LANCL1 | mRNA | -0.56 | 4.20E-02 | 7.38E-01 |
| NYNRIN | mRNA | -0.56 | 4.04E-02 | 7.38E-01 |
| WNT10A | mRNA | -0.56 | 7.60E-02 | 7.38E-01 |
| CLSTN3 | mRNA | -0.56 | 2.16E-02 | 7.38E-01 |
| RGPD5 | mRNA | -0.56 | 9.67E-02 | 7.42E-01 |
| LAMB3 | mRNA | -0.56 | 5.69E-02 | 7.38E-01 |
| TACC1 | mRNA | -0.56 | 1.56E-02 | 7.38E-01 |
| ABI2 | mRNA | -0.56 | 3.44E-02 | 7.38E-01 |
| RNF2 | mRNA | -0.56 | 4.17E-02 | 7.38E-01 |
| OSBPL5 | mRNA | -0.56 | 4.62E-02 | 7.38E-01 |
| NCOA7 | mRNA | -0.55 | 2.50E-02 | 7.38E-01 |
| ITM2B | mRNA | -0.55 | 1.56E-02 | 7.38E-01 |
| ALCAM | mRNA | -0.55 | 6.70E-02 | 7.38E-01 |
| KREMEN1 | mRNA | -0.55 | 3.80E-02 | 7.38E-01 |
| SSC5D | mRNA | -0.55 | 3.27E-02 | 7.38E-01 |
| ABTB1 | mRNA | -0.55 | 3.70E-02 | 7.38E-01 |
| SEPSECS | mRNA | -0.55 | 8.35E-02 | 7.38E-01 |
| ERBIN | mRNA | -0.55 | 1.74E-02 | 7.38E-01 |
| ZDHHC2 | mRNA | -0.55 | 6.82E-02 | 7.38E-01 |
| ELOVL5 | mRNA | -0.55 | 1.00E-02 | 7.38E-01 |
| VASH1 | mRNA | -0.55 | 4.44E-02 | 7.38E-01 |
| APOBEC3F | mRNA | -0.55 | 1.43E-02 | 7.38E-01 |
| UBL3 | mRNA | -0.55 | 3.08E-02 | 7.38E-01 |
| GINM1 | mRNA | -0.55 | 5.50E-02 | 7.38E-01 |
| HACE1 | mRNA | -0.55 | 6.16E-02 | 7.38E-01 |
| ERMP1 | mRNA | -0.55 | 2.53E-02 | 7.38E-01 |
| SYNPO | mRNA | -0.55 | 1.34E-02 | 7.38E-01 |
| NNT | mRNA | -0.55 | 7.98E-02 | 7.38E-01 |
| TTBK2 | mRNA | -0.54 | 5.87E-02 | 7.38E-01 |
| LETMD1 | mRNA | -0.54 | 3.54E-02 | 7.38E-01 |
| RNF213 | mRNA | -0.54 | 1.11E-02 | 7.38E-01 |
| ZBTB41 | mRNA | -0.54 | 2.98E-02 | 7.38E-01 |
| MPDZ | mRNA | -0.54 | 7.01E-02 | 7.38E-01 |
| LIMA1 | mRNA | -0.54 | 1.87E-02 | 7.38E-01 |
| RAB11FIP5 | mRNA | -0.54 | 3.85E-02 | 7.38E-01 |
| NDFIP1 | mRNA | -0.54 | 2.80E-02 | 7.38E-01 |
| PBX1 | mRNA | -0.54 | 3.30E-02 | 7.38E-01 |
| SBF2 | mRNA | -0.54 | 5.15E-02 | 7.38E-01 |
| PIK3R1 | mRNA | -0.54 | 9.19E-02 | 7.42E-01 |
| NFATC1 | mRNA | -0.54 | 7.47E-02 | 7.38E-01 |
| MARF1 | mRNA | -0.54 | 1.53E-02 | 7.38E-01 |
| PAK1 | mRNA | -0.54 | 6.26E-02 | 7.38E-01 |
| PDP2 | mRNA | -0.54 | 1.59E-02 | 7.38E-01 |
| MAST4 | mRNA | -0.54 | 2.10E-02 | 7.38E-01 |
| SMARCA1 | mRNA | -0.54 | 1.79E-02 | 7.38E-01 |
| BMPR2 | mRNA | -0.53 | 8.59E-02 | 7.41E-01 |
| SASH1 | mRNA | -0.53 | 2.51E-02 | 7.38E-01 |
| OSR2 | mRNA | -0.53 | 5.52E-02 | 7.38E-01 |
| IL13RA1 | mRNA | -0.53 | 5.52E-02 | 7.38E-01 |
| MSLN | mRNA | -0.53 | 2.20E-02 | 7.38E-01 |
| SNX14 | mRNA | -0.53 | 1.47E-02 | 7.38E-01 |
| UBE2J1 | mRNA | -0.52 | 2.58E-02 | 7.38E-01 |
| ATG5 | mRNA | -0.52 | 4.68E-02 | 7.38E-01 |
| ADAM22 | mRNA | -0.52 | 3.07E-02 | 7.38E-01 |
| TRIQK | mRNA | -0.52 | 2.81E-02 | 7.38E-01 |
| NIPBL | mRNA | -0.52 | 3.86E-02 | 7.38E-01 |
| NCF2 | mRNA | -0.52 | 8.69E-02 | 7.42E-01 |
| ARID5B | mRNA | -0.52 | 2.52E-02 | 7.38E-01 |
| PPP1R14C | mRNA | -0.52 | 6.02E-02 | 7.38E-01 |
| FAM20C | mRNA | -0.52 | 5.32E-02 | 7.38E-01 |
| MAP3K7 | mRNA | -0.51 | 4.11E-02 | 7.38E-01 |
| GNG10 | mRNA | -0.51 | 2.50E-02 | 7.38E-01 |
| LMBRD2 | mRNA | -0.51 | 4.16E-02 | 7.38E-01 |
| KIAA1109 | mRNA | -0.51 | 1.48E-02 | 7.38E-01 |
| PHF3 | mRNA | -0.51 | 2.66E-02 | 7.38E-01 |
| ADD3 | mRNA | -0.51 | 1.33E-02 | 7.38E-01 |
| AMOT | mRNA | -0.51 | 2.88E-02 | 7.38E-01 |
| SRGAP2 | mRNA | -0.51 | 7.16E-02 | 7.38E-01 |
| VPS13D | mRNA | -0.51 | 2.43E-02 | 7.38E-01 |
| ACACB | mRNA | -0.51 | 3.99E-02 | 7.38E-01 |
| KLHL36 | mRNA | -0.51 | 2.75E-02 | 7.38E-01 |
| TBC1D4 | mRNA | -0.51 | 2.97E-02 | 7.38E-01 |
| SKP2 | mRNA | -0.51 | 8.27E-02 | 7.38E-01 |
| ST3GAL2 | mRNA | -0.51 | 6.53E-02 | 7.38E-01 |
| HERC3 | mRNA | -0.51 | 7.96E-02 | 7.38E-01 |
| TLE2 | mRNA | -0.51 | 3.88E-02 | 7.38E-01 |
| CBLB | mRNA | -0.51 | 1.79E-02 | 7.38E-01 |
| SARAF | mRNA | -0.50 | 3.82E-02 | 7.38E-01 |
| TBC1D16 | mRNA | -0.50 | 3.49E-02 | 7.38E-01 |
| WDR27 | mRNA | -0.50 | 7.05E-02 | 7.38E-01 |
| PIK3C2A | mRNA | -0.50 | 3.24E-02 | 7.38E-01 |
| MDN1 | mRNA | -0.50 | 2.65E-02 | 7.38E-01 |
| AFDN | mRNA | -0.50 | 2.26E-02 | 7.38E-01 |
| HERC1 | mRNA | -0.50 | 3.50E-02 | 7.38E-01 |
| PLEKHM3 | mRNA | -0.50 | 6.00E-02 | 7.38E-01 |
| WWP1 | mRNA | -0.50 | 1.64E-02 | 7.38E-01 |
| CD99L2 | mRNA | -0.50 | 1.39E-02 | 7.38E-01 |
| PCED1B | mRNA | -0.50 | 2.72E-02 | 7.38E-01 |
| TAB3 | mRNA | -0.50 | 7.22E-02 | 7.38E-01 |
| BICD1 | mRNA | -0.50 | 4.25E-02 | 7.38E-01 |
| TCEA2 | mRNA | -0.50 | 7.32E-02 | 7.38E-01 |
| ATRN | mRNA | -0.50 | 1.92E-02 | 7.38E-01 |
| IGF1R | mRNA | -0.50 | 2.87E-02 | 7.38E-01 |
| CLDN15 | mRNA | -0.50 | 7.15E-02 | 7.38E-01 |
| STXBP1 | mRNA | -0.50 | 9.52E-02 | 7.42E-01 |
| PLEKHG1 | mRNA | -0.50 | 7.30E-02 | 7.38E-01 |
| TLE1 | mRNA | -0.49 | 2.97E-02 | 7.38E-01 |
| TMEM30A | mRNA | -0.49 | 4.85E-02 | 7.38E-01 |
| SMIM14 | mRNA | -0.49 | 2.99E-02 | 7.38E-01 |
| ZNF462 | mRNA | -0.49 | 4.88E-02 | 7.38E-01 |
| ACAT1 | mRNA | -0.49 | 6.60E-02 | 7.38E-01 |
| UBXN7 | mRNA | -0.49 | 2.87E-02 | 7.38E-01 |
| POMGNT1 | mRNA | -0.49 | 2.98E-02 | 7.38E-01 |
| CEP170 | mRNA | -0.49 | 9.47E-02 | 7.42E-01 |
| PAPSS1 | mRNA | -0.49 | 3.64E-02 | 7.38E-01 |
| NPNT | mRNA | -0.49 | 6.92E-02 | 7.38E-01 |
| PRKD3 | mRNA | -0.49 | 9.12E-02 | 7.42E-01 |
| CPNE3 | mRNA | -0.49 | 3.57E-02 | 7.38E-01 |
| MMGT1 | mRNA | -0.48 | 6.91E-02 | 7.38E-01 |
| LATS2 | mRNA | -0.48 | 9.13E-02 | 7.42E-01 |
| ANKRD46 | mRNA | -0.48 | 9.33E-02 | 7.42E-01 |
| RRM2B | mRNA | -0.48 | 4.13E-02 | 7.38E-01 |
| RRNAD1 | mRNA | -0.48 | 3.26E-02 | 7.38E-01 |
| MOB1B | mRNA | -0.48 | 6.09E-02 | 7.38E-01 |
| PRTG | mRNA | -0.48 | 9.38E-02 | 7.42E-01 |
| NXPE3 | mRNA | -0.48 | 6.41E-02 | 7.38E-01 |
| MAGI3 | mRNA | -0.48 | 7.12E-02 | 7.38E-01 |
| C14orf93 | mRNA | -0.48 | 5.43E-02 | 7.38E-01 |
| UFL1 | mRNA | -0.48 | 4.00E-02 | 7.38E-01 |
| BCLAF3 | mRNA | -0.48 | 5.54E-02 | 7.38E-01 |
| HIPK3 | mRNA | -0.48 | 8.83E-02 | 7.42E-01 |
| LTB4R | mRNA | -0.48 | 9.80E-02 | 7.42E-01 |
| RALGAPA1 | mRNA | -0.48 | 6.39E-02 | 7.38E-01 |
| C5orf15 | mRNA | -0.47 | 7.19E-02 | 7.38E-01 |
| PLEKHA4 | mRNA | -0.47 | 6.45E-02 | 7.38E-01 |
| ABLIM1 | mRNA | -0.47 | 5.71E-02 | 7.38E-01 |
| MICA | mRNA | -0.47 | 7.46E-02 | 7.38E-01 |
| RSPH3 | mRNA | -0.47 | 5.57E-02 | 7.38E-01 |
| NLN | mRNA | -0.47 | 5.57E-02 | 7.38E-01 |
| PLEKHB1 | mRNA | -0.47 | 3.19E-02 | 7.38E-01 |
| TNKS | mRNA | -0.47 | 2.29E-02 | 7.38E-01 |
| CARD11 | mRNA | -0.47 | 3.38E-02 | 7.38E-01 |
| DSP | mRNA | -0.47 | 1.68E-02 | 7.38E-01 |
| AHNAK | mRNA | -0.47 | 8.79E-02 | 7.42E-01 |
| MEGF8 | mRNA | -0.47 | 5.40E-02 | 7.38E-01 |
| PRKCI | mRNA | -0.47 | 4.68E-02 | 7.38E-01 |
| GNAI1 | mRNA | -0.47 | 6.02E-02 | 7.38E-01 |
| KIAA2026 | mRNA | -0.47 | 8.89E-02 | 7.42E-01 |
| PTPN3 | mRNA | -0.47 | 4.51E-02 | 7.38E-01 |
| RALGPS2 | mRNA | -0.46 | 2.49E-02 | 7.38E-01 |
| PJA2 | mRNA | -0.46 | 5.02E-02 | 7.38E-01 |
| PTAR1 | mRNA | -0.46 | 6.18E-02 | 7.38E-01 |
| LAMB2 | mRNA | -0.46 | 7.44E-02 | 7.38E-01 |
| IBTK | mRNA | -0.46 | 5.33E-02 | 7.38E-01 |
| ACLY | mRNA | -0.46 | 9.06E-02 | 7.42E-01 |
| DDAH2 | mRNA | -0.46 | 3.42E-02 | 7.38E-01 |
| NEK9 | mRNA | -0.46 | 5.88E-02 | 7.38E-01 |
| MATN2 | mRNA | -0.46 | 7.16E-02 | 7.38E-01 |
| SHC1 | mRNA | -0.46 | 8.51E-02 | 7.39E-01 |
| EPPK1 | mRNA | -0.46 | 2.85E-02 | 7.38E-01 |
| SUB1 | mRNA | -0.46 | 1.73E-02 | 7.38E-01 |
| RNF215 | mRNA | -0.46 | 8.63E-02 | 7.42E-01 |
| MTMR6 | mRNA | -0.46 | 7.65E-02 | 7.38E-01 |
| LEPR | mRNA | -0.46 | 4.16E-02 | 7.38E-01 |
| NBPF19 | mRNA | -0.46 | 4.10E-02 | 7.38E-01 |
| CD81 | mRNA | -0.46 | 5.28E-02 | 7.38E-01 |
| KRAS | mRNA | -0.45 | 7.62E-02 | 7.38E-01 |
| HOOK1 | mRNA | -0.45 | 3.87E-02 | 7.38E-01 |
| ARHGEF17 | mRNA | -0.45 | 4.72E-02 | 7.38E-01 |
| SNAPC2 | mRNA | -0.45 | 4.44E-02 | 7.38E-01 |
| COQ5 | mRNA | -0.45 | 8.46E-02 | 7.38E-01 |
| PRKAB2 | mRNA | -0.45 | 9.17E-02 | 7.42E-01 |
| PHF21A | mRNA | -0.45 | 1.84E-02 | 7.38E-01 |
| KLHL7 | mRNA | -0.45 | 5.16E-02 | 7.38E-01 |
| SSH2 | mRNA | -0.45 | 5.32E-02 | 7.38E-01 |
| SUN1 | mRNA | -0.45 | 5.31E-02 | 7.38E-01 |
| MOSPD2 | mRNA | -0.45 | 4.29E-02 | 7.38E-01 |
| CDC42EP3 | mRNA | -0.45 | 5.26E-02 | 7.38E-01 |
| TLN1 | mRNA | -0.45 | 2.44E-02 | 7.38E-01 |
| PRCP | mRNA | -0.45 | 9.90E-02 | 7.42E-01 |
| IGF2BP3 | mRNA | -0.45 | 7.29E-02 | 7.38E-01 |
| CCDC50 | mRNA | -0.45 | 1.96E-02 | 7.38E-01 |
| GNG12 | mRNA | -0.45 | 3.17E-02 | 7.38E-01 |
| RASSF5 | mRNA | -0.45 | 7.52E-02 | 7.38E-01 |
| SCCPDH | mRNA | -0.44 | 9.90E-02 | 7.42E-01 |
| SPOCK2 | mRNA | -0.44 | 8.63E-02 | 7.42E-01 |
| OSTM1 | mRNA | -0.44 | 4.19E-02 | 7.38E-01 |
| PAWR | mRNA | -0.44 | 7.61E-02 | 7.38E-01 |
| SH3BGRL2 | mRNA | -0.44 | 6.15E-02 | 7.38E-01 |
| FIG4 | mRNA | -0.44 | 5.39E-02 | 7.38E-01 |
| ZNF496 | mRNA | -0.44 | 3.71E-02 | 7.38E-01 |
| ASL | mRNA | -0.44 | 8.94E-02 | 7.42E-01 |
| ARHGAP12 | mRNA | -0.44 | 3.15E-02 | 7.38E-01 |
| BCAM | mRNA | -0.44 | 8.08E-02 | 7.38E-01 |
| ELOVL6 | mRNA | -0.44 | 6.47E-02 | 7.38E-01 |
| GDF11 | mRNA | -0.44 | 9.33E-02 | 7.42E-01 |
| FKBP9 | mRNA | -0.44 | 5.45E-02 | 7.38E-01 |
| HSPG2 | mRNA | -0.44 | 8.97E-02 | 7.42E-01 |
| VPS13A | mRNA | -0.44 | 2.78E-02 | 7.38E-01 |
| TRIM4 | mRNA | -0.44 | 9.28E-02 | 7.42E-01 |
| CYLD | mRNA | -0.44 | 8.17E-02 | 7.38E-01 |
| FAM169A | mRNA | -0.43 | 9.13E-02 | 7.42E-01 |
| INPP5F | mRNA | -0.43 | 9.75E-02 | 7.42E-01 |
| CRTAP | mRNA | -0.43 | 4.31E-02 | 7.38E-01 |
| GCC2 | mRNA | -0.43 | 3.07E-02 | 7.38E-01 |
| GOLPH3 | mRNA | -0.43 | 2.90E-02 | 7.38E-01 |
| MACF1 | mRNA | -0.43 | 2.34E-02 | 7.38E-01 |
| ZYG11B | mRNA | -0.43 | 4.55E-02 | 7.38E-01 |
| TMEM164 | mRNA | -0.43 | 2.51E-02 | 7.38E-01 |
| FOXO3 | mRNA | -0.43 | 7.94E-02 | 7.38E-01 |
| SALL4 | mRNA | -0.43 | 8.93E-02 | 7.42E-01 |
| SNX7 | mRNA | -0.43 | 7.23E-02 | 7.38E-01 |
| WDR19 | mRNA | -0.43 | 4.36E-02 | 7.38E-01 |
| FZD2 | mRNA | -0.43 | 5.46E-02 | 7.38E-01 |
| CERS5 | mRNA | -0.43 | 8.43E-02 | 7.38E-01 |
| BBX | mRNA | -0.42 | 7.85E-02 | 7.38E-01 |
| SMOX | mRNA | -0.42 | 6.22E-02 | 7.38E-01 |
| RNF38 | mRNA | -0.42 | 7.18E-02 | 7.38E-01 |
| AHR | mRNA | -0.42 | 9.04E-02 | 7.42E-01 |
| PRKAR2B | mRNA | -0.42 | 3.21E-02 | 7.38E-01 |
| USP24 | mRNA | -0.42 | 2.73E-02 | 7.38E-01 |
| DPH5 | mRNA | -0.42 | 8.90E-02 | 7.42E-01 |
| CNOT6L | mRNA | -0.42 | 9.92E-02 | 7.42E-01 |
| RBL2 | mRNA | -0.42 | 9.52E-02 | 7.42E-01 |
| LRP11 | mRNA | -0.42 | 7.50E-02 | 7.38E-01 |
| LAMP2 | mRNA | -0.42 | 8.73E-02 | 7.42E-01 |
| ZKSCAN8 | mRNA | -0.42 | 9.34E-02 | 7.42E-01 |
| SLC25A36 | mRNA | -0.42 | 5.99E-02 | 7.38E-01 |
| ST6GAL1 | mRNA | -0.42 | 3.95E-02 | 7.38E-01 |
| MYH14 | mRNA | -0.42 | 2.41E-02 | 7.38E-01 |
| SENP6 | mRNA | -0.42 | 8.63E-02 | 7.42E-01 |
| FNDC3A | mRNA | -0.42 | 5.95E-02 | 7.38E-01 |
| HEATR5B | mRNA | -0.42 | 4.82E-02 | 7.38E-01 |
| TCEAL9 | mRNA | -0.41 | 5.93E-02 | 7.38E-01 |
| ZNF704 | mRNA | -0.41 | 5.37E-02 | 7.38E-01 |
| LAMA5 | mRNA | -0.41 | 2.68E-02 | 7.38E-01 |
| CCNC | mRNA | -0.41 | 4.29E-02 | 7.38E-01 |
| PRKAA1 | mRNA | -0.41 | 4.04E-02 | 7.38E-01 |
| MICALL2 | mRNA | -0.41 | 4.41E-02 | 7.38E-01 |
| CNN3 | mRNA | -0.41 | 3.90E-02 | 7.38E-01 |
| LMBRD1 | mRNA | -0.41 | 3.71E-02 | 7.38E-01 |
| CYBRD1 | mRNA | -0.41 | 5.52E-02 | 7.38E-01 |
| UBOX5 | mRNA | -0.41 | 6.57E-02 | 7.38E-01 |
| CFAP36 | mRNA | -0.41 | 4.83E-02 | 7.38E-01 |
| SEC14L2 | mRNA | -0.41 | 6.03E-02 | 7.38E-01 |
| UBE2W | mRNA | -0.41 | 7.18E-02 | 7.38E-01 |
| DLG1 | mRNA | -0.41 | 3.52E-02 | 7.38E-01 |
| BAZ2B | mRNA | -0.41 | 5.90E-02 | 7.38E-01 |
| UBR1 | mRNA | -0.41 | 8.41E-02 | 7.38E-01 |
| PRR14L | mRNA | -0.40 | 5.41E-02 | 7.38E-01 |
| DIP2B | mRNA | -0.40 | 7.11E-02 | 7.38E-01 |
| DSTYK | mRNA | -0.40 | 6.20E-02 | 7.38E-01 |
| FRY | mRNA | -0.40 | 9.81E-02 | 7.42E-01 |
| KIAA1549 | mRNA | -0.40 | 4.72E-02 | 7.38E-01 |
| HERPUD2 | mRNA | -0.40 | 8.97E-02 | 7.42E-01 |
| STX12 | mRNA | -0.40 | 9.65E-02 | 7.42E-01 |
| TAGLN2 | mRNA | -0.40 | 5.18E-02 | 7.38E-01 |
| KLF11 | mRNA | -0.40 | 9.99E-02 | 7.42E-01 |
| KDM5B | mRNA | -0.40 | 4.97E-02 | 7.38E-01 |
| PPP2R5A | mRNA | -0.40 | 8.17E-02 | 7.38E-01 |
| C1GALT1 | mRNA | -0.40 | 5.71E-02 | 7.38E-01 |
| SINHCAF | mRNA | -0.40 | 9.50E-02 | 7.42E-01 |
| XRN1 | mRNA | -0.40 | 3.56E-02 | 7.38E-01 |
| ALDH7A1 | mRNA | -0.40 | 5.14E-02 | 7.38E-01 |
| PIK3CA | mRNA | -0.40 | 3.98E-02 | 7.38E-01 |
| TRAF3IP2 | mRNA | -0.40 | 6.31E-02 | 7.38E-01 |
| TAF1D | mRNA | -0.40 | 9.29E-02 | 7.42E-01 |
| RSBN1 | mRNA | -0.40 | 4.67E-02 | 7.38E-01 |
| NUP58 | mRNA | -0.39 | 8.14E-02 | 7.38E-01 |
| ATXN1 | mRNA | -0.39 | 5.92E-02 | 7.38E-01 |
| BIRC2 | mRNA | -0.39 | 8.78E-02 | 7.42E-01 |
| COL18A1 | mRNA | -0.39 | 3.44E-02 | 7.38E-01 |
| SH3GLB1 | mRNA | -0.39 | 3.89E-02 | 7.38E-01 |
| TNKS2 | mRNA | -0.39 | 3.56E-02 | 7.38E-01 |
| KHNYN | mRNA | -0.39 | 4.30E-02 | 7.38E-01 |
| MAST2 | mRNA | -0.39 | 9.64E-02 | 7.42E-01 |
| CD163L1 | mRNA | -0.39 | 8.80E-02 | 7.42E-01 |
| PKIG | mRNA | -0.39 | 5.86E-02 | 7.38E-01 |
| HECTD4 | mRNA | -0.39 | 4.67E-02 | 7.38E-01 |
| JUND | mRNA | -0.39 | 7.52E-02 | 7.38E-01 |
| GNG7 | mRNA | -0.39 | 9.11E-02 | 7.42E-01 |
| BNIP2 | mRNA | -0.39 | 9.71E-02 | 7.42E-01 |
| FEM1B | mRNA | -0.39 | 6.43E-02 | 7.38E-01 |
| TMEM245 | mRNA | -0.38 | 5.98E-02 | 7.38E-01 |
| MYO5B | mRNA | -0.38 | 5.45E-02 | 7.38E-01 |
| WIPI2 | mRNA | -0.38 | 6.56E-02 | 7.38E-01 |
| YES1 | mRNA | -0.38 | 5.76E-02 | 7.38E-01 |
| EXOC1 | mRNA | -0.38 | 5.72E-02 | 7.38E-01 |
| PDPR | mRNA | -0.38 | 5.79E-02 | 7.38E-01 |
| ATP11B | mRNA | -0.38 | 7.64E-02 | 7.38E-01 |
| NBEAL1 | mRNA | -0.38 | 7.87E-02 | 7.38E-01 |
| CPEB4 | mRNA | -0.38 | 3.87E-02 | 7.38E-01 |
| NAV1 | mRNA | -0.38 | 9.56E-02 | 7.42E-01 |
| EEF1A1 | mRNA | -0.38 | 7.07E-02 | 7.38E-01 |
| RAB3GAP2 | mRNA | -0.38 | 3.88E-02 | 7.38E-01 |
| BMP1 | mRNA | -0.38 | 5.91E-02 | 7.38E-01 |
| RAP1GAP2 | mRNA | -0.38 | 8.74E-02 | 7.42E-01 |
| IREB2 | mRNA | -0.38 | 3.97E-02 | 7.38E-01 |
| SOS1 | mRNA | -0.38 | 9.54E-02 | 7.42E-01 |
| SH3YL1 | mRNA | -0.38 | 6.41E-02 | 7.38E-01 |
| VPS54 | mRNA | -0.38 | 9.71E-02 | 7.42E-01 |
| TCAF1 | mRNA | -0.37 | 8.26E-02 | 7.38E-01 |
| UBR4 | mRNA | -0.37 | 4.04E-02 | 7.38E-01 |
| SETD5 | mRNA | -0.37 | 8.46E-02 | 7.38E-01 |
| SH3BP4 | mRNA | -0.37 | 8.31E-02 | 7.38E-01 |
| USP32 | mRNA | -0.37 | 5.94E-02 | 7.38E-01 |
| RAB3IP | mRNA | -0.37 | 9.01E-02 | 7.42E-01 |
| NCOA1 | mRNA | -0.37 | 8.91E-02 | 7.42E-01 |
| WDTC1 | mRNA | -0.37 | 5.04E-02 | 7.38E-01 |
| EEA1 | mRNA | -0.37 | 8.98E-02 | 7.42E-01 |
| CSGALNACT1 | mRNA | -0.37 | 6.33E-02 | 7.38E-01 |
| KDM3A | mRNA | -0.37 | 6.12E-02 | 7.38E-01 |
| HOOK3 | mRNA | -0.37 | 8.88E-02 | 7.42E-01 |
| GNAQ | mRNA | -0.37 | 4.86E-02 | 7.38E-01 |
| ITFG1 | mRNA | -0.37 | 8.39E-02 | 7.38E-01 |
| FBXO2 | mRNA | -0.37 | 6.61E-02 | 7.38E-01 |
| GPALPP1 | mRNA | -0.36 | 8.21E-02 | 7.38E-01 |
| FCHSD2 | mRNA | -0.36 | 9.27E-02 | 7.42E-01 |
| B4GALT5 | mRNA | -0.36 | 9.16E-02 | 7.42E-01 |
| SECISBP2L | mRNA | -0.36 | 6.29E-02 | 7.38E-01 |
| SPG11 | mRNA | -0.36 | 7.26E-02 | 7.38E-01 |
| SEC24B | mRNA | -0.36 | 7.12E-02 | 7.38E-01 |
| NCOA3 | mRNA | -0.36 | 5.09E-02 | 7.38E-01 |
| TENT2 | mRNA | -0.36 | 9.52E-02 | 7.42E-01 |
| PRKAR1A | mRNA | -0.36 | 6.71E-02 | 7.38E-01 |
| CHD9 | mRNA | -0.36 | 6.48E-02 | 7.38E-01 |
| CHD2 | mRNA | -0.36 | 4.17E-02 | 7.38E-01 |
| MEIS3 | mRNA | -0.36 | 7.38E-02 | 7.38E-01 |
| DENND5A | mRNA | -0.36 | 7.79E-02 | 7.38E-01 |
| NF1 | mRNA | -0.35 | 7.38E-02 | 7.38E-01 |
| HMGN3 | mRNA | -0.35 | 8.35E-02 | 7.38E-01 |
| EGLN1 | mRNA | -0.35 | 5.84E-02 | 7.38E-01 |
| C5orf22 | mRNA | -0.35 | 9.07E-02 | 7.42E-01 |
| NBEA | mRNA | -0.35 | 9.79E-02 | 7.42E-01 |
| DST | mRNA | -0.35 | 7.19E-02 | 7.38E-01 |
| EIF4B | mRNA | -0.35 | 7.60E-02 | 7.38E-01 |
| LMO7 | mRNA | -0.35 | 7.87E-02 | 7.38E-01 |
| CSNK1G3 | mRNA | -0.35 | 9.06E-02 | 7.42E-01 |
| CREBL2 | mRNA | -0.35 | 4.83E-02 | 7.38E-01 |
| ICE1 | mRNA | -0.35 | 6.67E-02 | 7.38E-01 |
| AFF1 | mRNA | -0.35 | 6.99E-02 | 7.38E-01 |
| PCM1 | mRNA | -0.35 | 6.81E-02 | 7.38E-01 |
| ZFC3H1 | mRNA | -0.35 | 8.13E-02 | 7.38E-01 |
| IPO7 | mRNA | -0.35 | 4.61E-02 | 7.38E-01 |
| SCAMP1 | mRNA | -0.35 | 5.88E-02 | 7.38E-01 |
| TMEM92 | mRNA | -0.34 | 7.85E-02 | 7.38E-01 |
| DNAJC21 | mRNA | -0.34 | 6.09E-02 | 7.38E-01 |
| PTBP3 | mRNA | -0.34 | 6.27E-02 | 7.38E-01 |
| LGR4 | mRNA | -0.34 | 6.18E-02 | 7.38E-01 |
| RAB5B | mRNA | -0.34 | 4.51E-02 | 7.38E-01 |
| EPG5 | mRNA | -0.34 | 7.39E-02 | 7.38E-01 |
| PAK2 | mRNA | -0.34 | 8.21E-02 | 7.38E-01 |
| TMCC1 | mRNA | -0.34 | 6.10E-02 | 7.38E-01 |
| TANC1 | mRNA | -0.34 | 8.68E-02 | 7.42E-01 |
| KCTD3 | mRNA | -0.34 | 8.97E-02 | 7.42E-01 |
| PGRMC2 | mRNA | -0.34 | 8.97E-02 | 7.42E-01 |
| BAZ2A | mRNA | -0.34 | 5.72E-02 | 7.38E-01 |
| SMAD5 | mRNA | -0.34 | 7.47E-02 | 7.38E-01 |
| SCP2 | mRNA | -0.34 | 5.57E-02 | 7.38E-01 |
| OSGIN2 | mRNA | -0.33 | 7.55E-02 | 7.38E-01 |
| HEATR5A | mRNA | -0.33 | 8.28E-02 | 7.38E-01 |
| YPEL5 | mRNA | -0.33 | 5.68E-02 | 7.38E-01 |
| FAM168A | mRNA | -0.33 | 8.30E-02 | 7.38E-01 |
| XPC | mRNA | -0.33 | 9.70E-02 | 7.42E-01 |
| GMCL1 | mRNA | -0.33 | 8.89E-02 | 7.42E-01 |
| SLC9A8 | mRNA | -0.33 | 7.97E-02 | 7.38E-01 |
| RNF44 | mRNA | -0.33 | 6.30E-02 | 7.38E-01 |
| BEX3 | mRNA | -0.33 | 5.61E-02 | 7.38E-01 |
| SCRN1 | mRNA | -0.33 | 9.40E-02 | 7.42E-01 |
| ABCC3 | mRNA | -0.33 | 5.92E-02 | 7.38E-01 |
| NRAS | mRNA | -0.32 | 9.92E-02 | 7.42E-01 |
| ALS2 | mRNA | -0.32 | 8.07E-02 | 7.38E-01 |
| MKLN1 | mRNA | -0.32 | 8.22E-02 | 7.38E-01 |
| AKAP13 | mRNA | -0.32 | 6.63E-02 | 7.38E-01 |
| ATP6V1H | mRNA | -0.32 | 8.34E-02 | 7.38E-01 |
| STIM1 | mRNA | -0.32 | 9.56E-02 | 7.42E-01 |
| FBXL18 | mRNA | -0.32 | 9.24E-02 | 7.42E-01 |
| MTDH | mRNA | -0.32 | 8.71E-02 | 7.42E-01 |
| PPP1CB | mRNA | -0.31 | 5.88E-02 | 7.38E-01 |
| COG3 | mRNA | -0.31 | 9.07E-02 | 7.42E-01 |
| USP9X | mRNA | -0.31 | 6.86E-02 | 7.38E-01 |
| ZBTB18 | mRNA | -0.31 | 9.85E-02 | 7.42E-01 |
| NOA1 | mRNA | -0.31 | 8.00E-02 | 7.38E-01 |
| AZIN1 | mRNA | -0.31 | 9.17E-02 | 7.42E-01 |
| RB1CC1 | mRNA | -0.31 | 7.46E-02 | 7.38E-01 |
| EPS15 | mRNA | -0.31 | 6.71E-02 | 7.38E-01 |
| UBR5 | mRNA | -0.31 | 6.47E-02 | 7.38E-01 |
| GLS | mRNA | -0.31 | 8.15E-02 | 7.38E-01 |
| SOGA1 | mRNA | -0.30 | 9.17E-02 | 7.42E-01 |
| BRWD1 | mRNA | -0.30 | 9.80E-02 | 7.42E-01 |
| ARIH1 | mRNA | -0.30 | 8.86E-02 | 7.42E-01 |
| TAX1BP1 | mRNA | -0.30 | 6.66E-02 | 7.38E-01 |
| SLU7 | mRNA | -0.30 | 7.32E-02 | 7.38E-01 |
| CNOT6 | mRNA | 0.30 | 7.55E-02 | 7.38E-01 |
| UMPS | mRNA | 0.30 | 9.23E-02 | 7.42E-01 |
| TIMM23 | mRNA | 0.30 | 8.28E-02 | 7.38E-01 |
| SEC61B | mRNA | 0.31 | 9.00E-02 | 7.42E-01 |
| TRIM16 | mRNA | 0.31 | 7.09E-02 | 7.38E-01 |
| RPS26 | mRNA | 0.31 | 8.45E-02 | 7.38E-01 |
| POMP | mRNA | 0.31 | 9.57E-02 | 7.42E-01 |
| TAPBPL | mRNA | 0.31 | 9.88E-02 | 7.42E-01 |
| VPS29 | mRNA | 0.31 | 8.06E-02 | 7.38E-01 |
| RUVBL1 | mRNA | 0.31 | 8.03E-02 | 7.38E-01 |
| PSMB5 | mRNA | 0.31 | 6.79E-02 | 7.38E-01 |
| NUBP2 | mRNA | 0.31 | 8.82E-02 | 7.42E-01 |
| SPATS2L | mRNA | 0.31 | 7.52E-02 | 7.38E-01 |
| CYC1 | mRNA | 0.31 | 6.83E-02 | 7.38E-01 |
| DGKZ | mRNA | 0.32 | 7.94E-02 | 7.38E-01 |
| MRPL51 | mRNA | 0.32 | 6.09E-02 | 7.38E-01 |
| ACIN1 | mRNA | 0.32 | 9.43E-02 | 7.42E-01 |
| SSRP1 | mRNA | 0.32 | 5.68E-02 | 7.38E-01 |
| EIF2B2 | mRNA | 0.32 | 6.71E-02 | 7.38E-01 |
| TPM3 | mRNA | 0.32 | 5.50E-02 | 7.38E-01 |
| NDUFS3 | mRNA | 0.32 | 7.63E-02 | 7.38E-01 |
| C11orf98 | mRNA | 0.32 | 7.20E-02 | 7.38E-01 |
| STAP2 | mRNA | 0.32 | 9.94E-02 | 7.42E-01 |
| AP1M1 | mRNA | 0.32 | 7.65E-02 | 7.38E-01 |
| DNAJA3 | mRNA | 0.32 | 7.97E-02 | 7.38E-01 |
| SLC2A6 | mRNA | 0.32 | 9.64E-02 | 7.42E-01 |
| MAFG | mRNA | 0.33 | 6.57E-02 | 7.38E-01 |
| ANXA4 | mRNA | 0.33 | 7.21E-02 | 7.38E-01 |
| CHERP | mRNA | 0.33 | 5.25E-02 | 7.38E-01 |
| UQCR11 | mRNA | 0.33 | 6.04E-02 | 7.38E-01 |
| NDUFB11 | mRNA | 0.33 | 6.61E-02 | 7.38E-01 |
| CFAP20 | mRNA | 0.33 | 6.85E-02 | 7.38E-01 |
| KPNA4 | mRNA | 0.33 | 6.29E-02 | 7.38E-01 |
| GALE | mRNA | 0.33 | 8.50E-02 | 7.39E-01 |
| LMF2 | mRNA | 0.33 | 9.78E-02 | 7.42E-01 |
| GOSR2 | mRNA | 0.33 | 9.40E-02 | 7.42E-01 |
| PSMB2 | mRNA | 0.33 | 6.77E-02 | 7.38E-01 |
| TMEM165 | mRNA | 0.33 | 6.04E-02 | 7.38E-01 |
| CAPN15 | mRNA | 0.33 | 5.88E-02 | 7.38E-01 |
| POLR2J | mRNA | 0.33 | 5.53E-02 | 7.38E-01 |
| CPSF6 | mRNA | 0.33 | 9.46E-02 | 7.42E-01 |
| PFDN6 | mRNA | 0.33 | 9.25E-02 | 7.42E-01 |
| RALY | mRNA | 0.34 | 6.54E-02 | 7.38E-01 |
| HIPK2 | mRNA | 0.34 | 7.35E-02 | 7.38E-01 |
| DDX56 | mRNA | 0.34 | 8.95E-02 | 7.42E-01 |
| CCT7 | mRNA | 0.34 | 6.73E-02 | 7.38E-01 |
| TSR1 | mRNA | 0.34 | 9.38E-02 | 7.42E-01 |
| PSMB3 | mRNA | 0.34 | 7.47E-02 | 7.38E-01 |
| MGAT1 | mRNA | 0.34 | 4.93E-02 | 7.38E-01 |
| RUVBL2 | mRNA | 0.34 | 6.09E-02 | 7.38E-01 |
| DRG1 | mRNA | 0.34 | 7.72E-02 | 7.38E-01 |
| CLPTM1 | mRNA | 0.34 | 9.79E-02 | 7.42E-01 |
| MRPL43 | mRNA | 0.34 | 6.98E-02 | 7.38E-01 |
| CCT6A | mRNA | 0.34 | 5.20E-02 | 7.38E-01 |
| EHMT1 | mRNA | 0.34 | 7.41E-02 | 7.38E-01 |
| SNRPB | mRNA | 0.34 | 7.84E-02 | 7.38E-01 |
| PSMA6 | mRNA | 0.34 | 6.89E-02 | 7.38E-01 |
| KHSRP | mRNA | 0.34 | 7.30E-02 | 7.38E-01 |
| FKBP4 | mRNA | 0.34 | 6.67E-02 | 7.38E-01 |
| TMEM126B | mRNA | 0.34 | 8.59E-02 | 7.41E-01 |
| PDZD11 | mRNA | 0.35 | 7.74E-02 | 7.38E-01 |
| TXNDC5 | mRNA | 0.35 | 9.81E-02 | 7.42E-01 |
| CTNNBL1 | mRNA | 0.35 | 7.09E-02 | 7.38E-01 |
| ALDH3A2 | mRNA | 0.35 | 8.64E-02 | 7.42E-01 |
| DHRS1 | mRNA | 0.35 | 8.06E-02 | 7.38E-01 |
| KAT2A | mRNA | 0.35 | 8.30E-02 | 7.38E-01 |
| BAG3 | mRNA | 0.35 | 6.64E-02 | 7.38E-01 |
| PGAM1 | mRNA | 0.35 | 5.34E-02 | 7.38E-01 |
| IMP4 | mRNA | 0.35 | 6.09E-02 | 7.38E-01 |
| TMX1 | mRNA | 0.35 | 5.97E-02 | 7.38E-01 |
| CLPP | mRNA | 0.35 | 5.78E-02 | 7.38E-01 |
| MRPL21 | mRNA | 0.35 | 7.61E-02 | 7.38E-01 |
| NUDT3 | mRNA | 0.35 | 7.97E-02 | 7.38E-01 |
| COMMD3 | mRNA | 0.35 | 9.37E-02 | 7.42E-01 |
| HDGF | mRNA | 0.35 | 4.66E-02 | 7.38E-01 |
| NANS | mRNA | 0.35 | 6.11E-02 | 7.38E-01 |
| LAD1 | mRNA | 0.35 | 5.17E-02 | 7.38E-01 |
| CDC123 | mRNA | 0.35 | 5.00E-02 | 7.38E-01 |
| PHF19 | mRNA | 0.35 | 9.15E-02 | 7.42E-01 |
| INO80E | mRNA | 0.36 | 6.14E-02 | 7.38E-01 |
| VOPP1 | mRNA | 0.36 | 6.27E-02 | 7.38E-01 |
| ERH | mRNA | 0.36 | 4.12E-02 | 7.38E-01 |
| TCF3 | mRNA | 0.36 | 5.09E-02 | 7.38E-01 |
| RXRA | mRNA | 0.36 | 5.68E-02 | 7.38E-01 |
| FUT3 | mRNA | 0.36 | 8.11E-02 | 7.38E-01 |
| ROMO1 | mRNA | 0.36 | 9.17E-02 | 7.42E-01 |
| BAD | mRNA | 0.36 | 5.72E-02 | 7.38E-01 |
| ELP4 | mRNA | 0.36 | 8.32E-02 | 7.38E-01 |
| RAB8A | mRNA | 0.36 | 4.45E-02 | 7.38E-01 |
| PMPCA | mRNA | 0.36 | 5.39E-02 | 7.38E-01 |
| S100A6 | mRNA | 0.36 | 6.23E-02 | 7.38E-01 |
| PPIL1 | mRNA | 0.36 | 5.42E-02 | 7.38E-01 |
| PDCD5 | mRNA | 0.36 | 8.82E-02 | 7.42E-01 |
| WDR5 | mRNA | 0.36 | 5.47E-02 | 7.38E-01 |
| COX7B | mRNA | 0.36 | 7.96E-02 | 7.38E-01 |
| GADD45GIP1 | mRNA | 0.36 | 7.50E-02 | 7.38E-01 |
| MRPS12 | mRNA | 0.36 | 5.20E-02 | 7.38E-01 |
| PGAM5 | mRNA | 0.36 | 4.39E-02 | 7.38E-01 |
| MRPL14 | mRNA | 0.36 | 8.21E-02 | 7.38E-01 |
| CENPN | mRNA | 0.36 | 7.05E-02 | 7.38E-01 |
| UQCRQ | mRNA | 0.36 | 4.73E-02 | 7.38E-01 |
| DNMT1 | mRNA | 0.36 | 4.65E-02 | 7.38E-01 |
| AIFM2 | mRNA | 0.36 | 9.43E-02 | 7.42E-01 |
| DOLPP1 | mRNA | 0.36 | 8.26E-02 | 7.38E-01 |
| ATP6V0E2 | mRNA | 0.36 | 5.80E-02 | 7.38E-01 |
| PPID | mRNA | 0.36 | 9.22E-02 | 7.42E-01 |
| EMG1 | mRNA | 0.36 | 9.14E-02 | 7.42E-01 |
| POLR2F | mRNA | 0.37 | 9.16E-02 | 7.42E-01 |
| NAA10 | mRNA | 0.37 | 6.19E-02 | 7.38E-01 |
| RBM28 | mRNA | 0.37 | 5.48E-02 | 7.38E-01 |
| MUL1 | mRNA | 0.37 | 9.30E-02 | 7.42E-01 |
| NASP | mRNA | 0.37 | 7.96E-02 | 7.38E-01 |
| HSD17B10 | mRNA | 0.37 | 8.84E-02 | 7.42E-01 |
| PCBD1 | mRNA | 0.37 | 5.96E-02 | 7.38E-01 |
| NDUFB10 | mRNA | 0.37 | 5.54E-02 | 7.38E-01 |
| RAD23A | mRNA | 0.37 | 4.28E-02 | 7.38E-01 |
| AATF | mRNA | 0.37 | 7.67E-02 | 7.38E-01 |
| HKDC1 | mRNA | 0.37 | 7.15E-02 | 7.38E-01 |
| VCP | mRNA | 0.37 | 6.78E-02 | 7.38E-01 |
| CYB561 | mRNA | 0.37 | 7.95E-02 | 7.38E-01 |
| MRPL3 | mRNA | 0.37 | 3.55E-02 | 7.38E-01 |
| S100A16 | mRNA | 0.37 | 9.91E-02 | 7.42E-01 |
| FKBP3 | mRNA | 0.37 | 4.75E-02 | 7.38E-01 |
| FIBP | mRNA | 0.37 | 4.77E-02 | 7.38E-01 |
| PSMD11 | mRNA | 0.37 | 4.65E-02 | 7.38E-01 |
| BCAT2 | mRNA | 0.37 | 9.65E-02 | 7.42E-01 |
| SAFB2 | mRNA | 0.37 | 8.76E-02 | 7.42E-01 |
| RAB5IF | mRNA | 0.38 | 6.78E-02 | 7.38E-01 |
| TSR3 | mRNA | 0.38 | 6.54E-02 | 7.38E-01 |
| AKT1 | mRNA | 0.38 | 5.67E-02 | 7.38E-01 |
| MFSD14B | mRNA | 0.38 | 3.58E-02 | 7.38E-01 |
| SHB | mRNA | 0.38 | 8.49E-02 | 7.39E-01 |
| CSAG1 | mRNA | 0.38 | 9.07E-02 | 7.42E-01 |
| PRPF38A | mRNA | 0.38 | 6.38E-02 | 7.38E-01 |
| COTL1 | mRNA | 0.38 | 9.03E-02 | 7.42E-01 |
| NDUFAF3 | mRNA | 0.38 | 5.65E-02 | 7.38E-01 |
| SF1 | mRNA | 0.38 | 7.98E-02 | 7.38E-01 |
| PAICS | mRNA | 0.38 | 8.75E-02 | 7.42E-01 |
| TMEM87A | mRNA | 0.38 | 8.74E-02 | 7.42E-01 |
| EIF4E2 | mRNA | 0.38 | 5.12E-02 | 7.38E-01 |
| KYNU | mRNA | 0.38 | 4.66E-02 | 7.38E-01 |
| GRIN1 | mRNA | 0.38 | 8.76E-02 | 7.42E-01 |
| POLR2I | mRNA | 0.38 | 4.77E-02 | 7.38E-01 |
| PPRC1 | mRNA | 0.38 | 6.20E-02 | 7.38E-01 |
| TXN | mRNA | 0.38 | 3.28E-02 | 7.38E-01 |
| GLRX3 | mRNA | 0.38 | 8.91E-02 | 7.42E-01 |
| TEAD4 | mRNA | 0.38 | 7.91E-02 | 7.38E-01 |
| PTPN18 | mRNA | 0.39 | 7.27E-02 | 7.38E-01 |
| COX15 | mRNA | 0.39 | 6.66E-02 | 7.38E-01 |
| PGP | mRNA | 0.39 | 8.18E-02 | 7.38E-01 |
| MEA1 | mRNA | 0.39 | 4.28E-02 | 7.38E-01 |
| SMIM12 | mRNA | 0.39 | 7.76E-02 | 7.38E-01 |
| UNG | mRNA | 0.39 | 9.65E-02 | 7.42E-01 |
| ISG20L2 | mRNA | 0.39 | 5.82E-02 | 7.38E-01 |
| NMRAL1 | mRNA | 0.39 | 3.23E-02 | 7.38E-01 |
| WDR46 | mRNA | 0.39 | 4.67E-02 | 7.38E-01 |
| SLC45A4 | mRNA | 0.39 | 3.66E-02 | 7.38E-01 |
| PSMC2 | mRNA | 0.39 | 7.25E-02 | 7.38E-01 |
| FIGNL1 | mRNA | 0.39 | 4.71E-02 | 7.38E-01 |
| POLR3A | mRNA | 0.39 | 4.96E-02 | 7.38E-01 |
| PSMC1 | mRNA | 0.39 | 4.87E-02 | 7.38E-01 |
| SEC61A1 | mRNA | 0.39 | 5.47E-02 | 7.38E-01 |
| ELOF1 | mRNA | 0.39 | 8.65E-02 | 7.42E-01 |
| TOMM5 | mRNA | 0.39 | 7.47E-02 | 7.38E-01 |
| ETFDH | mRNA | 0.39 | 9.64E-02 | 7.42E-01 |
| GOLT1A | mRNA | 0.39 | 9.64E-02 | 7.42E-01 |
| NCDN | mRNA | 0.39 | 9.98E-02 | 7.42E-01 |
| TSEN2 | mRNA | 0.39 | 9.93E-02 | 7.42E-01 |
| ARHGDIB | mRNA | 0.39 | 9.20E-02 | 7.42E-01 |
| MESD | mRNA | 0.39 | 5.00E-02 | 7.38E-01 |
| EDF1 | mRNA | 0.39 | 6.85E-02 | 7.38E-01 |
| SLC52A2 | mRNA | 0.39 | 7.36E-02 | 7.38E-01 |
| RPS6KA4 | mRNA | 0.40 | 8.33E-02 | 7.38E-01 |
| RAB15 | mRNA | 0.40 | 7.24E-02 | 7.38E-01 |
| FAM111A | mRNA | 0.40 | 4.93E-02 | 7.38E-01 |
| CASP6 | mRNA | 0.40 | 9.87E-02 | 7.42E-01 |
| NRARP | mRNA | 0.40 | 8.36E-02 | 7.38E-01 |
| MRPS34 | mRNA | 0.40 | 3.86E-02 | 7.38E-01 |
| HSPA8 | mRNA | 0.40 | 3.42E-02 | 7.38E-01 |
| LSM3 | mRNA | 0.40 | 5.95E-02 | 7.38E-01 |
| DYSF | mRNA | 0.40 | 8.56E-02 | 7.40E-01 |
| CEBPD | mRNA | 0.40 | 9.11E-02 | 7.42E-01 |
| ZBTB9 | mRNA | 0.40 | 7.66E-02 | 7.38E-01 |
| SWI5 | mRNA | 0.40 | 5.77E-02 | 7.38E-01 |
| TPI1 | mRNA | 0.40 | 2.76E-02 | 7.38E-01 |
| FAM174B | mRNA | 0.40 | 9.39E-02 | 7.42E-01 |
| PRDX1 | mRNA | 0.40 | 3.28E-02 | 7.38E-01 |
| SEC61G | mRNA | 0.40 | 4.35E-02 | 7.38E-01 |
| HSPBP1 | mRNA | 0.40 | 7.42E-02 | 7.38E-01 |
| SF3B4 | mRNA | 0.40 | 8.46E-02 | 7.38E-01 |
| PA2G4 | mRNA | 0.41 | 2.93E-02 | 7.38E-01 |
| RRP36 | mRNA | 0.41 | 9.66E-02 | 7.42E-01 |
| GGCT | mRNA | 0.41 | 8.42E-02 | 7.38E-01 |
| RNF208 | mRNA | 0.41 | 5.40E-02 | 7.38E-01 |
| SEC13 | mRNA | 0.41 | 3.97E-02 | 7.38E-01 |
| ATP6V1G1 | mRNA | 0.41 | 4.17E-02 | 7.38E-01 |
| FAM50A | mRNA | 0.41 | 4.49E-02 | 7.38E-01 |
| MRPL50 | mRNA | 0.41 | 8.36E-02 | 7.38E-01 |
| PHLDA1 | mRNA | 0.41 | 5.07E-02 | 7.38E-01 |
| NDUFA6 | mRNA | 0.41 | 7.77E-02 | 7.38E-01 |
| POLE4 | mRNA | 0.41 | 6.96E-02 | 7.38E-01 |
| MRPS17 | mRNA | 0.41 | 4.28E-02 | 7.38E-01 |
| CCNQ | mRNA | 0.41 | 7.06E-02 | 7.38E-01 |
| DKC1 | mRNA | 0.41 | 6.91E-02 | 7.38E-01 |
| MST1R | mRNA | 0.41 | 9.42E-02 | 7.42E-01 |
| NOP2 | mRNA | 0.42 | 3.73E-02 | 7.38E-01 |
| ATG7 | mRNA | 0.42 | 4.53E-02 | 7.38E-01 |
| MRPS2 | mRNA | 0.42 | 4.10E-02 | 7.38E-01 |
| UROD | mRNA | 0.42 | 8.56E-02 | 7.40E-01 |
| ZNF668 | mRNA | 0.42 | 9.92E-02 | 7.42E-01 |
| NOC4L | mRNA | 0.42 | 5.07E-02 | 7.38E-01 |
| MAP2K3 | mRNA | 0.42 | 5.26E-02 | 7.38E-01 |
| FBL | mRNA | 0.42 | 5.98E-02 | 7.38E-01 |
| MRPL24 | mRNA | 0.42 | 3.32E-02 | 7.38E-01 |
| COQ6 | mRNA | 0.42 | 9.06E-02 | 7.42E-01 |
| PSMB10 | mRNA | 0.42 | 7.73E-02 | 7.38E-01 |
| JTB | mRNA | 0.42 | 8.14E-02 | 7.38E-01 |
| ICMT | mRNA | 0.42 | 2.93E-02 | 7.38E-01 |
| EMC9 | mRNA | 0.42 | 4.71E-02 | 7.38E-01 |
| UBE2J2 | mRNA | 0.42 | 6.38E-02 | 7.38E-01 |
| CTU2 | mRNA | 0.42 | 9.49E-02 | 7.42E-01 |
| ARL6IP4 | mRNA | 0.42 | 3.19E-02 | 7.38E-01 |
| GSK3A | mRNA | 0.42 | 3.08E-02 | 7.38E-01 |
| UTP15 | mRNA | 0.42 | 7.70E-02 | 7.38E-01 |
| UPP1 | mRNA | 0.43 | 8.33E-02 | 7.38E-01 |
| CCDC51 | mRNA | 0.43 | 8.59E-02 | 7.41E-01 |
| TCOF1 | mRNA | 0.43 | 6.24E-02 | 7.38E-01 |
| SSBP1 | mRNA | 0.43 | 3.95E-02 | 7.38E-01 |
| SH3TC2 | mRNA | 0.43 | 7.44E-02 | 7.38E-01 |
| PPP1CA | mRNA | 0.43 | 6.43E-02 | 7.38E-01 |
| ABCF2 | mRNA | 0.43 | 7.26E-02 | 7.38E-01 |
| OAS3 | mRNA | 0.43 | 2.64E-02 | 7.38E-01 |
| VAV1 | mRNA | 0.43 | 7.90E-02 | 7.38E-01 |
| ATP5MF | mRNA | 0.43 | 2.57E-02 | 7.38E-01 |
| S100P | mRNA | 0.43 | 8.74E-02 | 7.42E-01 |
| BAZ1A | mRNA | 0.43 | 2.37E-02 | 7.38E-01 |
| TJP2 | mRNA | 0.43 | 8.40E-02 | 7.38E-01 |
| HAUS1 | mRNA | 0.43 | 6.74E-02 | 7.38E-01 |
| ZDHHC6 | mRNA | 0.44 | 4.67E-02 | 7.38E-01 |
| TXNL4A | mRNA | 0.44 | 4.90E-02 | 7.38E-01 |
| CHCHD2 | mRNA | 0.44 | 3.43E-02 | 7.38E-01 |
| TRMT10C | mRNA | 0.44 | 9.60E-02 | 7.42E-01 |
| TMEM138 | mRNA | 0.44 | 5.73E-02 | 7.38E-01 |
| TMEM40 | mRNA | 0.44 | 6.71E-02 | 7.38E-01 |
| AURKAIP1 | mRNA | 0.44 | 3.32E-02 | 7.38E-01 |
| TUBB4B | mRNA | 0.44 | 8.96E-02 | 7.42E-01 |
| RRS1 | mRNA | 0.44 | 4.59E-02 | 7.38E-01 |
| BID | mRNA | 0.44 | 4.00E-02 | 7.38E-01 |
| FOXM1 | mRNA | 0.44 | 6.88E-02 | 7.38E-01 |
| ENTR1 | mRNA | 0.44 | 4.56E-02 | 7.38E-01 |
| WRAP53 | mRNA | 0.44 | 5.86E-02 | 7.38E-01 |
| EI24 | mRNA | 0.44 | 2.91E-02 | 7.38E-01 |
| C1QTNF6 | mRNA | 0.44 | 9.26E-02 | 7.42E-01 |
| ZNF282 | mRNA | 0.44 | 7.03E-02 | 7.38E-01 |
| PRELID1 | mRNA | 0.45 | 2.17E-02 | 7.38E-01 |
| RAN | mRNA | 0.45 | 3.18E-02 | 7.38E-01 |
| RBFA | mRNA | 0.45 | 7.64E-02 | 7.38E-01 |
| APRT | mRNA | 0.45 | 7.74E-02 | 7.38E-01 |
| TIMM22 | mRNA | 0.45 | 7.74E-02 | 7.38E-01 |
| EMP2 | mRNA | 0.45 | 6.42E-02 | 7.38E-01 |
| UBE2N | mRNA | 0.45 | 2.41E-02 | 7.38E-01 |
| ATP2C2 | mRNA | 0.45 | 9.47E-02 | 7.42E-01 |
| BOLA3 | mRNA | 0.45 | 8.20E-02 | 7.38E-01 |
| STX3 | mRNA | 0.45 | 7.75E-02 | 7.38E-01 |
| RBM14 | mRNA | 0.45 | 1.97E-02 | 7.38E-01 |
| ITGAE | mRNA | 0.45 | 8.24E-02 | 7.38E-01 |
| TPRKB | mRNA | 0.45 | 7.66E-02 | 7.38E-01 |
| ATP5ME | mRNA | 0.45 | 4.51E-02 | 7.38E-01 |
| MAGED1 | mRNA | 0.45 | 8.90E-02 | 7.42E-01 |
| TBC1D22A | mRNA | 0.45 | 6.48E-02 | 7.38E-01 |
| NOLC1 | mRNA | 0.45 | 4.18E-02 | 7.38E-01 |
| MRPL41 | mRNA | 0.45 | 8.76E-02 | 7.42E-01 |
| NOC2L | mRNA | 0.45 | 2.30E-02 | 7.38E-01 |
| KLHDC3 | mRNA | 0.45 | 4.11E-02 | 7.38E-01 |
| AUP1 | mRNA | 0.45 | 9.37E-02 | 7.42E-01 |
| MYEOV | mRNA | 0.45 | 2.32E-02 | 7.38E-01 |
| PMF1 | mRNA | 0.45 | 5.05E-02 | 7.38E-01 |
| CCDC167 | mRNA | 0.45 | 9.95E-02 | 7.42E-01 |
| FXYD3 | mRNA | 0.45 | 5.09E-02 | 7.38E-01 |
| SNRPC | mRNA | 0.45 | 2.50E-02 | 7.38E-01 |
| ARHGDIA | mRNA | 0.46 | 8.72E-02 | 7.42E-01 |
| MAGOHB | mRNA | 0.46 | 7.01E-02 | 7.38E-01 |
| BCL7B | mRNA | 0.46 | 6.82E-02 | 7.38E-01 |
| PRTFDC1 | mRNA | 0.46 | 7.13E-02 | 7.38E-01 |
| SNRPF | mRNA | 0.46 | 6.33E-02 | 7.38E-01 |
| TNPO3 | mRNA | 0.46 | 2.82E-02 | 7.38E-01 |
| MPHOSPH10 | mRNA | 0.46 | 6.40E-02 | 7.38E-01 |
| FARSA | mRNA | 0.46 | 2.16E-02 | 7.38E-01 |
| NDUFB9 | mRNA | 0.46 | 3.77E-02 | 7.38E-01 |
| PPCDC | mRNA | 0.46 | 9.78E-02 | 7.42E-01 |
| EIPR1 | mRNA | 0.46 | 3.49E-02 | 7.38E-01 |
| TUBG1 | mRNA | 0.46 | 8.80E-02 | 7.42E-01 |
| EIF5A | mRNA | 0.46 | 2.54E-02 | 7.38E-01 |
| TTC9 | mRNA | 0.46 | 6.53E-02 | 7.38E-01 |
| CHPF2 | mRNA | 0.46 | 1.87E-02 | 7.38E-01 |
| SMIM22 | mRNA | 0.46 | 4.37E-02 | 7.38E-01 |
| AHSA1 | mRNA | 0.46 | 2.47E-02 | 7.38E-01 |
| CHAF1B | mRNA | 0.47 | 7.19E-02 | 7.38E-01 |
| MRPS11 | mRNA | 0.47 | 3.15E-02 | 7.38E-01 |
| HS3ST1 | mRNA | 0.47 | 9.68E-02 | 7.42E-01 |
| PSMA3 | mRNA | 0.47 | 2.13E-02 | 7.38E-01 |
| C1orf159 | mRNA | 0.47 | 4.00E-02 | 7.38E-01 |
| AAGAB | mRNA | 0.47 | 2.40E-02 | 7.38E-01 |
| MGAT4A | mRNA | 0.47 | 7.36E-02 | 7.38E-01 |
| PSMA7 | mRNA | 0.47 | 5.08E-02 | 7.38E-01 |
| PYCR3 | mRNA | 0.47 | 6.38E-02 | 7.38E-01 |
| PLPP2 | mRNA | 0.47 | 3.10E-02 | 7.38E-01 |
| LAGE3 | mRNA | 0.47 | 8.36E-02 | 7.38E-01 |
| TBX6 | mRNA | 0.47 | 3.65E-02 | 7.38E-01 |
| NFKBIA | mRNA | 0.47 | 5.19E-02 | 7.38E-01 |
| MANEAL | mRNA | 0.47 | 3.97E-02 | 7.38E-01 |
| ANXA11 | mRNA | 0.47 | 3.62E-02 | 7.38E-01 |
| RPA2 | mRNA | 0.47 | 5.75E-02 | 7.38E-01 |
| BRCA2 | mRNA | 0.48 | 7.41E-02 | 7.38E-01 |
| CLP1 | mRNA | 0.48 | 7.03E-02 | 7.38E-01 |
| NUDC | mRNA | 0.48 | 4.01E-02 | 7.38E-01 |
| ATP5MC1 | mRNA | 0.48 | 6.93E-02 | 7.38E-01 |
| PSMC4 | mRNA | 0.48 | 1.72E-02 | 7.38E-01 |
| SLC25A10 | mRNA | 0.48 | 7.15E-02 | 7.38E-01 |
| PDAP1 | mRNA | 0.48 | 1.47E-02 | 7.38E-01 |
| CSTB | mRNA | 0.48 | 2.97E-02 | 7.38E-01 |
| PRMT1 | mRNA | 0.48 | 5.96E-02 | 7.38E-01 |
| ISOC2 | mRNA | 0.48 | 3.38E-02 | 7.38E-01 |
| COPS6 | mRNA | 0.48 | 1.67E-02 | 7.38E-01 |
| CDC7 | mRNA | 0.48 | 8.07E-02 | 7.38E-01 |
| SUV39H2 | mRNA | 0.48 | 8.11E-02 | 7.38E-01 |
| AHCYL2 | mRNA | 0.48 | 6.18E-02 | 7.38E-01 |
| PSMC3 | mRNA | 0.48 | 2.86E-02 | 7.38E-01 |
| SLC35E1 | mRNA | 0.48 | 3.43E-02 | 7.38E-01 |
| RECQL4 | mRNA | 0.48 | 6.62E-02 | 7.38E-01 |
| CALB2 | mRNA | 0.49 | 1.72E-02 | 7.38E-01 |
| UTP4 | mRNA | 0.49 | 1.96E-02 | 7.38E-01 |
| HK2 | mRNA | 0.49 | 8.55E-02 | 7.40E-01 |
| SRPRB | mRNA | 0.49 | 3.05E-02 | 7.38E-01 |
| JMJD4 | mRNA | 0.49 | 8.51E-02 | 7.39E-01 |
| CCDC86 | mRNA | 0.49 | 2.54E-02 | 7.38E-01 |
| RPP25 | mRNA | 0.49 | 4.01E-02 | 7.38E-01 |
| EZH2 | mRNA | 0.49 | 1.91E-02 | 7.38E-01 |
| ZNF468 | mRNA | 0.49 | 8.68E-02 | 7.42E-01 |
| CHTF18 | mRNA | 0.49 | 7.56E-02 | 7.38E-01 |
| BRI3 | mRNA | 0.49 | 6.78E-02 | 7.38E-01 |
| IRAK1 | mRNA | 0.49 | 5.64E-02 | 7.38E-01 |
| CTPS1 | mRNA | 0.49 | 1.93E-02 | 7.38E-01 |
| SLCO4A1 | mRNA | 0.49 | 1.81E-02 | 7.38E-01 |
| CHCHD3 | mRNA | 0.49 | 9.78E-02 | 7.42E-01 |
| PSMC5 | mRNA | 0.49 | 2.89E-02 | 7.38E-01 |
| FBXL6 | mRNA | 0.49 | 7.56E-02 | 7.38E-01 |
| SIRT6 | mRNA | 0.49 | 4.50E-02 | 7.38E-01 |
| COA4 | mRNA | 0.49 | 2.51E-02 | 7.38E-01 |
| MGAT4B | mRNA | 0.49 | 5.95E-02 | 7.38E-01 |
| TNFSF13 | mRNA | 0.50 | 8.49E-02 | 7.39E-01 |
| EXOSC3 | mRNA | 0.50 | 8.43E-02 | 7.38E-01 |
| RNASEH2A | mRNA | 0.50 | 5.36E-02 | 7.38E-01 |
| IMP3 | mRNA | 0.50 | 7.70E-02 | 7.38E-01 |
| TIMM10 | mRNA | 0.50 | 5.49E-02 | 7.38E-01 |
| MPDU1 | mRNA | 0.50 | 8.08E-02 | 7.38E-01 |
| MRPL58 | mRNA | 0.50 | 5.39E-02 | 7.38E-01 |
| PKN1 | mRNA | 0.50 | 1.81E-02 | 7.38E-01 |
| TMEM104 | mRNA | 0.50 | 3.89E-02 | 7.38E-01 |
| CRYBB2 | mRNA | 0.50 | 8.96E-02 | 7.42E-01 |
| PCDH1 | mRNA | 0.50 | 4.86E-02 | 7.38E-01 |
| MYO19 | mRNA | 0.50 | 2.81E-02 | 7.38E-01 |
| RFT1 | mRNA | 0.50 | 3.79E-02 | 7.38E-01 |
| ZNF587B | mRNA | 0.50 | 6.06E-02 | 7.38E-01 |
| SLC27A4 | mRNA | 0.50 | 3.47E-02 | 7.38E-01 |
| PDXP | mRNA | 0.50 | 7.18E-02 | 7.38E-01 |
| MRPL40 | mRNA | 0.50 | 6.69E-02 | 7.38E-01 |
| BCS1L | mRNA | 0.51 | 4.82E-02 | 7.38E-01 |
| LSM7 | mRNA | 0.51 | 2.69E-02 | 7.38E-01 |
| PFKFB3 | mRNA | 0.51 | 3.27E-02 | 7.38E-01 |
| MROH6 | mRNA | 0.51 | 8.78E-02 | 7.42E-01 |
| SAMD1 | mRNA | 0.51 | 1.44E-02 | 7.38E-01 |
| SCFD2 | mRNA | 0.51 | 5.99E-02 | 7.38E-01 |
| BLCAP | mRNA | 0.51 | 1.67E-02 | 7.38E-01 |
| CUL3 | mRNA | 0.51 | 1.16E-02 | 7.38E-01 |
| ALG12 | mRNA | 0.51 | 8.18E-02 | 7.38E-01 |
| TIMM13 | mRNA | 0.51 | 5.36E-02 | 7.38E-01 |
| EXOSC2 | mRNA | 0.51 | 3.86E-02 | 7.38E-01 |
| NCLN | mRNA | 0.51 | 1.86E-02 | 7.38E-01 |
| TMEM201 | mRNA | 0.52 | 2.56E-02 | 7.38E-01 |
| DCTPP1 | mRNA | 0.52 | 3.24E-02 | 7.38E-01 |
| BRMS1L | mRNA | 0.52 | 9.68E-02 | 7.42E-01 |
| DHFR | mRNA | 0.52 | 4.28E-02 | 7.38E-01 |
| NUBP1 | mRNA | 0.52 | 7.85E-02 | 7.38E-01 |
| PMM2 | mRNA | 0.52 | 4.71E-02 | 7.38E-01 |
| FANCM | mRNA | 0.52 | 6.60E-02 | 7.38E-01 |
| ARMCX5 | mRNA | 0.52 | 8.34E-02 | 7.38E-01 |
| CARD10 | mRNA | 0.52 | 1.67E-02 | 7.38E-01 |
| POLR2C | mRNA | 0.52 | 2.12E-02 | 7.38E-01 |
| DDX31 | mRNA | 0.53 | 4.26E-02 | 7.38E-01 |
| SLC25A22 | mRNA | 0.53 | 2.52E-02 | 7.38E-01 |
| CEP152 | mRNA | 0.53 | 8.24E-02 | 7.38E-01 |
| UCK1 | mRNA | 0.53 | 3.78E-02 | 7.38E-01 |
| PSME2 | mRNA | 0.53 | 4.00E-02 | 7.38E-01 |
| POLR1C | mRNA | 0.53 | 5.51E-02 | 7.38E-01 |
| NFKBIZ | mRNA | 0.53 | 7.84E-02 | 7.38E-01 |
| HPDL | mRNA | 0.53 | 5.92E-02 | 7.38E-01 |
| CEACAM19 | mRNA | 0.53 | 6.44E-02 | 7.38E-01 |
| TRAPPC2L | mRNA | 0.53 | 2.68E-02 | 7.38E-01 |
| UQCC3 | mRNA | 0.53 | 4.97E-02 | 7.38E-01 |
| GMDS | mRNA | 0.53 | 6.84E-02 | 7.38E-01 |
| ZNF701 | mRNA | 0.53 | 7.39E-02 | 7.38E-01 |
| NABP2 | mRNA | 0.54 | 1.83E-02 | 7.38E-01 |
| ALDH1B1 | mRNA | 0.54 | 4.69E-02 | 7.38E-01 |
| YKT6 | mRNA | 0.54 | 1.37E-02 | 7.38E-01 |
| SRSF7 | mRNA | 0.54 | 7.03E-02 | 7.38E-01 |
| MRPL23 | mRNA | 0.54 | 1.67E-02 | 7.38E-01 |
| UFD1 | mRNA | 0.54 | 4.09E-02 | 7.38E-01 |
| CHRM3 | mRNA | 0.54 | 7.45E-02 | 7.38E-01 |
| PLEKHN1 | mRNA | 0.54 | 9.53E-02 | 7.42E-01 |
| TRAF4 | mRNA | 0.54 | 2.07E-02 | 7.38E-01 |
| DNAJC9 | mRNA | 0.54 | 1.44E-02 | 7.38E-01 |
| PHPT1 | mRNA | 0.54 | 2.37E-02 | 7.38E-01 |
| MRPL20 | mRNA | 0.54 | 2.13E-02 | 7.38E-01 |
| POLE | mRNA | 0.54 | 1.39E-02 | 7.38E-01 |
| SOX7 | mRNA | 0.54 | 7.14E-02 | 7.38E-01 |
| SLC25A19 | mRNA | 0.54 | 6.92E-02 | 7.38E-01 |
| FEZ2 | mRNA | 0.55 | 1.40E-02 | 7.38E-01 |
| MRPL46 | mRNA | 0.55 | 7.21E-02 | 7.38E-01 |
| TUBGCP5 | mRNA | 0.55 | 5.70E-02 | 7.38E-01 |
| ZNF503 | mRNA | 0.55 | 3.41E-02 | 7.38E-01 |
| ZMYND19 | mRNA | 0.55 | 1.78E-02 | 7.38E-01 |
| DARS2 | mRNA | 0.55 | 2.08E-02 | 7.38E-01 |
| XBP1 | mRNA | 0.55 | 3.35E-02 | 7.38E-01 |
| FOXRED2 | mRNA | 0.55 | 2.99E-02 | 7.38E-01 |
| COMMD4 | mRNA | 0.55 | 3.38E-02 | 7.38E-01 |
| NUP35 | mRNA | 0.55 | 6.72E-02 | 7.38E-01 |
| RPS19BP1 | mRNA | 0.55 | 4.58E-02 | 7.38E-01 |
| FAM217B | mRNA | 0.55 | 8.32E-02 | 7.38E-01 |
| TRA2B | mRNA | 0.55 | 1.79E-02 | 7.38E-01 |
| RAP1GAP | mRNA | 0.56 | 7.84E-02 | 7.38E-01 |
| LRRC59 | mRNA | 0.56 | 1.05E-02 | 7.38E-01 |
| DAGLA | mRNA | 0.56 | 9.19E-02 | 7.42E-01 |
| HAUS7 | mRNA | 0.56 | 6.06E-02 | 7.38E-01 |
| RRP1 | mRNA | 0.56 | 7.69E-02 | 7.38E-01 |
| PTGES2 | mRNA | 0.56 | 8.94E-03 | 7.38E-01 |
| PDE8A | mRNA | 0.56 | 3.74E-02 | 7.38E-01 |
| NCAPH2 | mRNA | 0.56 | 2.48E-02 | 7.38E-01 |
| EMC10 | mRNA | 0.56 | 8.78E-02 | 7.42E-01 |
| SLC4A2 | mRNA | 0.56 | 9.95E-02 | 7.42E-01 |
| CDK2AP1 | mRNA | 0.56 | 3.33E-02 | 7.38E-01 |
| SLC19A1 | mRNA | 0.56 | 4.63E-02 | 7.38E-01 |
| FANCC | mRNA | 0.56 | 4.28E-02 | 7.38E-01 |
| DTYMK | mRNA | 0.57 | 1.50E-02 | 7.38E-01 |
| AKR1B10 | mRNA | 0.57 | 4.06E-02 | 7.38E-01 |
| TYMS | mRNA | 0.57 | 9.84E-02 | 7.42E-01 |
| NDUFB2 | mRNA | 0.57 | 5.22E-02 | 7.38E-01 |
| SGO1 | mRNA | 0.57 | 6.64E-02 | 7.38E-01 |
| INTS2 | mRNA | 0.57 | 6.40E-02 | 7.38E-01 |
| EIF4EBP2 | mRNA | 0.57 | 1.58E-02 | 7.38E-01 |
| MRPL12 | mRNA | 0.57 | 7.34E-03 | 7.38E-01 |
| ST3GAL1 | mRNA | 0.57 | 3.08E-02 | 7.38E-01 |
| IFT27 | mRNA | 0.57 | 1.34E-02 | 7.38E-01 |
| CMSS1 | mRNA | 0.57 | 2.18E-02 | 7.38E-01 |
| RPUSD1 | mRNA | 0.57 | 7.23E-02 | 7.38E-01 |
| MCRIP2 | mRNA | 0.57 | 4.27E-02 | 7.38E-01 |
| SIVA1 | mRNA | 0.58 | 2.91E-02 | 7.38E-01 |
| SCO2 | mRNA | 0.58 | 6.26E-02 | 7.38E-01 |
| XRCC2 | mRNA | 0.58 | 9.26E-02 | 7.42E-01 |
| THOP1 | mRNA | 0.58 | 1.12E-02 | 7.38E-01 |
| RFC3 | mRNA | 0.58 | 5.57E-02 | 7.38E-01 |
| ABCB8 | mRNA | 0.58 | 2.32E-02 | 7.38E-01 |
| PKN3 | mRNA | 0.58 | 8.48E-02 | 7.38E-01 |
| RFC5 | mRNA | 0.58 | 4.81E-02 | 7.38E-01 |
| CENPU | mRNA | 0.59 | 3.03E-02 | 7.38E-01 |
| BOP1 | mRNA | 0.59 | 2.78E-02 | 7.38E-01 |
| MRPL57 | mRNA | 0.59 | 2.39E-02 | 7.38E-01 |
| HGH1 | mRNA | 0.59 | 8.04E-02 | 7.38E-01 |
| CPLX1 | mRNA | 0.59 | 8.47E-02 | 7.38E-01 |
| POP7 | mRNA | 0.59 | 1.15E-02 | 7.38E-01 |
| GINS3 | mRNA | 0.59 | 2.92E-02 | 7.38E-01 |
| NFKBIB | mRNA | 0.59 | 3.57E-02 | 7.38E-01 |
| CDC6 | mRNA | 0.59 | 5.22E-02 | 7.38E-01 |
| RBM19 | mRNA | 0.59 | 5.05E-02 | 7.38E-01 |
| TIMM8A | mRNA | 0.59 | 6.70E-02 | 7.38E-01 |
| UTP14A | mRNA | 0.60 | 4.72E-02 | 7.38E-01 |
| ALDH16A1 | mRNA | 0.60 | 5.15E-02 | 7.38E-01 |
| BRMS1 | mRNA | 0.60 | 2.62E-02 | 7.38E-01 |
| USP16 | mRNA | 0.60 | 8.14E-02 | 7.38E-01 |
| OSGEP | mRNA | 0.60 | 6.09E-02 | 7.38E-01 |
| SLC43A3 | mRNA | 0.60 | 6.11E-02 | 7.38E-01 |
| FAM83A | mRNA | 0.60 | 2.53E-02 | 7.38E-01 |
| RPL27 | mRNA | 0.60 | 1.41E-02 | 7.38E-01 |
| PPARGC1B | mRNA | 0.60 | 8.42E-02 | 7.38E-01 |
| NSUN6 | mRNA | 0.60 | 6.84E-02 | 7.38E-01 |
| XRCC3 | mRNA | 0.60 | 1.31E-02 | 7.38E-01 |
| PAQR4 | mRNA | 0.60 | 9.52E-03 | 7.38E-01 |
| RMI1 | mRNA | 0.60 | 3.87E-02 | 7.38E-01 |
| TMEM30B | mRNA | 0.60 | 1.40E-02 | 7.38E-01 |
| CCER2 | mRNA | 0.60 | 7.42E-02 | 7.38E-01 |
| BATF | mRNA | 0.60 | 6.38E-02 | 7.38E-01 |
| FBXL16 | mRNA | 0.61 | 1.57E-02 | 7.38E-01 |
| ATP5PO | mRNA | 0.61 | 1.00E-02 | 7.38E-01 |
| LRR1 | mRNA | 0.61 | 2.80E-02 | 7.38E-01 |
| DPY19L3 | mRNA | 0.61 | 7.01E-02 | 7.38E-01 |
| GINS4 | mRNA | 0.61 | 3.62E-02 | 7.38E-01 |
| ICAM3 | mRNA | 0.61 | 3.85E-02 | 7.38E-01 |
| RNF222 | mRNA | 0.61 | 8.42E-02 | 7.38E-01 |
| TCF19 | mRNA | 0.61 | 2.66E-02 | 7.38E-01 |
| ZNF488 | mRNA | 0.61 | 4.34E-02 | 7.38E-01 |
| SRSF3 | mRNA | 0.61 | 9.81E-02 | 7.42E-01 |
| CAMK2N2 | mRNA | 0.61 | 4.97E-02 | 7.38E-01 |
| CHCHD10 | mRNA | 0.62 | 4.94E-02 | 7.38E-01 |
| SFXN4 | mRNA | 0.62 | 5.74E-02 | 7.38E-01 |
| SLIRP | mRNA | 0.62 | 5.32E-02 | 7.38E-01 |
| RANBP1 | mRNA | 0.62 | 1.47E-02 | 7.38E-01 |
| SPNS2 | mRNA | 0.62 | 2.21E-02 | 7.38E-01 |
| EXOSC4 | mRNA | 0.62 | 4.50E-02 | 7.38E-01 |
| SIK1B | mRNA | 0.62 | 8.19E-02 | 7.38E-01 |
| B3GNT9 | mRNA | 0.62 | 3.22E-02 | 7.38E-01 |
| ZPR1 | mRNA | 0.63 | 3.47E-02 | 7.38E-01 |
| ACSL5 | mRNA | 0.63 | 2.39E-02 | 7.38E-01 |
| POLA2 | mRNA | 0.63 | 1.32E-02 | 7.38E-01 |
| HPS1 | mRNA | 0.63 | 7.30E-02 | 7.38E-01 |
| VEGFC | mRNA | 0.63 | 5.05E-02 | 7.38E-01 |
| B3GALT6 | mRNA | 0.63 | 7.25E-02 | 7.38E-01 |
| ZNF557 | mRNA | 0.63 | 9.81E-02 | 7.42E-01 |
| DOCK10 | mRNA | 0.63 | 1.76E-02 | 7.38E-01 |
| NDUFAF8 | mRNA | 0.63 | 2.04E-02 | 7.38E-01 |
| GLRX5 | mRNA | 0.63 | 1.34E-02 | 7.38E-01 |
| POLA1 | mRNA | 0.63 | 1.24E-02 | 7.38E-01 |
| SIGMAR1 | mRNA | 0.63 | 9.79E-03 | 7.38E-01 |
| LIG1 | mRNA | 0.64 | 4.00E-02 | 7.38E-01 |
| IKBKE | mRNA | 0.64 | 4.04E-02 | 7.38E-01 |
| CTNS | mRNA | 0.64 | 1.72E-02 | 7.38E-01 |
| TOMM40 | mRNA | 0.64 | 9.30E-03 | 7.38E-01 |
| TNFRSF11A | mRNA | 0.64 | 2.78E-02 | 7.38E-01 |
| RRP9 | mRNA | 0.64 | 5.26E-02 | 7.38E-01 |
| CENPP | mRNA | 0.64 | 9.01E-02 | 7.42E-01 |
| MCM4 | mRNA | 0.64 | 1.27E-02 | 7.38E-01 |
| GPS2 | mRNA | 0.64 | 7.26E-02 | 7.38E-01 |
| FANCA | mRNA | 0.64 | 7.46E-02 | 7.38E-01 |
| CORO1A | mRNA | 0.64 | 6.81E-02 | 7.38E-01 |
| QTRT1 | mRNA | 0.64 | 6.90E-02 | 7.38E-01 |
| RAD51 | mRNA | 0.65 | 7.71E-02 | 7.38E-01 |
| COMMD1 | mRNA | 0.65 | 9.33E-02 | 7.42E-01 |
| ADM5 | mRNA | 0.65 | 3.77E-02 | 7.38E-01 |
| POLE3 | mRNA | 0.65 | 5.32E-03 | 7.38E-01 |
| CENPX | mRNA | 0.65 | 7.56E-02 | 7.38E-01 |
| OIP5 | mRNA | 0.65 | 6.66E-02 | 7.38E-01 |
| E2F1 | mRNA | 0.65 | 2.30E-02 | 7.38E-01 |
| IL20RA | mRNA | 0.65 | 9.85E-02 | 7.42E-01 |
| AARSD1 | mRNA | 0.65 | 3.80E-02 | 7.38E-01 |
| TREX1 | mRNA | 0.65 | 6.35E-02 | 7.38E-01 |
| CHAF1A | mRNA | 0.65 | 1.79E-02 | 7.38E-01 |
| SLC47A1 | mRNA | 0.65 | 7.79E-02 | 7.38E-01 |
| ABHD16B | mRNA | 0.66 | 4.10E-02 | 7.38E-01 |
| PCBD2 | mRNA | 0.66 | 4.57E-02 | 7.38E-01 |
| HNRNPAB | mRNA | 0.66 | 6.25E-03 | 7.38E-01 |
| PCLAF | mRNA | 0.66 | 2.44E-02 | 7.38E-01 |
| TRAIP | mRNA | 0.66 | 5.26E-02 | 7.38E-01 |
| ZNF836 | mRNA | 0.66 | 9.80E-02 | 7.42E-01 |
| MCM6 | mRNA | 0.66 | 2.66E-02 | 7.38E-01 |
| XYLB | mRNA | 0.66 | 9.63E-02 | 7.42E-01 |
| ZNF107 | mRNA | 0.66 | 4.21E-02 | 7.38E-01 |
| PYCR1 | mRNA | 0.67 | 3.00E-02 | 7.38E-01 |
| FUS | mRNA | 0.67 | 9.90E-02 | 7.42E-01 |
| BTBD6 | mRNA | 0.67 | 8.71E-02 | 7.42E-01 |
| GRHL3 | mRNA | 0.67 | 5.48E-02 | 7.38E-01 |
| KLF4 | mRNA | 0.67 | 4.58E-02 | 7.38E-01 |
| COMMD10 | mRNA | 0.67 | 3.57E-02 | 7.38E-01 |
| TELO2 | mRNA | 0.67 | 1.77E-02 | 7.38E-01 |
| RPL12 | mRNA | 0.67 | 1.04E-02 | 7.38E-01 |
| ORC1 | mRNA | 0.68 | 5.55E-02 | 7.38E-01 |
| ZNF614 | mRNA | 0.68 | 5.67E-02 | 7.38E-01 |
| TIPIN | mRNA | 0.68 | 3.79E-02 | 7.38E-01 |
| NFKBID | mRNA | 0.68 | 2.81E-02 | 7.38E-01 |
| TONSL | mRNA | 0.68 | 5.63E-03 | 7.38E-01 |
| CENPS-CORT | mRNA | 0.68 | 5.38E-02 | 7.38E-01 |
| TK1 | mRNA | 0.68 | 3.59E-02 | 7.38E-01 |
| ZNF367 | mRNA | 0.68 | 5.87E-02 | 7.38E-01 |
| MARS2 | mRNA | 0.68 | 5.47E-02 | 7.38E-01 |
| ADIRF | mRNA | 0.68 | 6.40E-02 | 7.38E-01 |
| BIRC5 | mRNA | 0.68 | 7.61E-02 | 7.38E-01 |
| NDUFAF6 | mRNA | 0.68 | 4.00E-02 | 7.38E-01 |
| BRI3BP | mRNA | 0.68 | 3.72E-02 | 7.38E-01 |
| EDN1 | mRNA | 0.68 | 1.29E-02 | 7.38E-01 |
| NOP16 | mRNA | 0.69 | 1.07E-02 | 7.38E-01 |
| RFC2 | mRNA | 0.69 | 7.08E-03 | 7.38E-01 |
| HELLS | mRNA | 0.69 | 7.86E-02 | 7.38E-01 |
| PEMT | mRNA | 0.69 | 1.42E-02 | 7.38E-01 |
| GLRX2 | mRNA | 0.69 | 5.89E-02 | 7.38E-01 |
| GPAT3 | mRNA | 0.69 | 8.38E-02 | 7.38E-01 |
| SRM | mRNA | 0.69 | 2.14E-02 | 7.38E-01 |
| MTG1 | mRNA | 0.69 | 8.67E-02 | 7.42E-01 |
| TMEM199 | mRNA | 0.69 | 5.09E-02 | 7.38E-01 |
| ACKR2 | mRNA | 0.69 | 1.46E-02 | 7.38E-01 |
| PCDHB9 | mRNA | 0.69 | 8.93E-02 | 7.42E-01 |
| ENKD1 | mRNA | 0.69 | 6.27E-02 | 7.38E-01 |
| ORC6 | mRNA | 0.69 | 3.77E-02 | 7.38E-01 |
| SLC37A1 | mRNA | 0.69 | 8.99E-03 | 7.38E-01 |
| GDF15 | mRNA | 0.70 | 2.80E-02 | 7.38E-01 |
| AMDHD2 | mRNA | 0.70 | 9.14E-02 | 7.42E-01 |
| SDCBP2 | mRNA | 0.71 | 2.05E-02 | 7.38E-01 |
| NCEH1 | mRNA | 0.71 | 2.19E-02 | 7.38E-01 |
| MYBL2 | mRNA | 0.71 | 9.24E-03 | 7.38E-01 |
| PRR15L | mRNA | 0.71 | 4.79E-02 | 7.38E-01 |
| GATD3B | mRNA | 0.71 | 6.07E-02 | 7.38E-01 |
| ATAD3A | mRNA | 0.71 | 4.39E-03 | 7.38E-01 |
| PCNA | mRNA | 0.71 | 6.51E-03 | 7.38E-01 |
| CHCHD1 | mRNA | 0.71 | 1.35E-02 | 7.38E-01 |
| PPAN | mRNA | 0.71 | 4.80E-02 | 7.38E-01 |
| IQCH | mRNA | 0.72 | 9.89E-02 | 7.42E-01 |
| DPF1 | mRNA | 0.72 | 7.94E-02 | 7.38E-01 |
| KHK | mRNA | 0.72 | 4.29E-02 | 7.38E-01 |
| FAM222A | mRNA | 0.72 | 7.29E-02 | 7.38E-01 |
| CENPS | mRNA | 0.72 | 2.69E-02 | 7.38E-01 |
| ZNF600 | mRNA | 0.73 | 7.85E-02 | 7.38E-01 |
| FRRS1 | mRNA | 0.73 | 6.16E-02 | 7.38E-01 |
| STRA6 | mRNA | 0.73 | 3.04E-03 | 7.38E-01 |
| ZNF829 | mRNA | 0.74 | 6.85E-02 | 7.38E-01 |
| GTF2IRD2 | mRNA | 0.74 | 7.62E-02 | 7.38E-01 |
| AEN | mRNA | 0.74 | 3.29E-02 | 7.38E-01 |
| SRSF2 | mRNA | 0.74 | 4.38E-02 | 7.38E-01 |
| SMIM11B | mRNA | 0.74 | 6.52E-02 | 7.38E-01 |
| MRTO4 | mRNA | 0.75 | 1.31E-02 | 7.38E-01 |
| DTL | mRNA | 0.75 | 2.58E-02 | 7.38E-01 |
| NOL12 | mRNA | 0.75 | 4.03E-02 | 7.38E-01 |
| C19orf25 | mRNA | 0.75 | 1.30E-02 | 7.38E-01 |
| MCM3 | mRNA | 0.75 | 6.34E-03 | 7.38E-01 |
| BCL2 | mRNA | 0.75 | 8.30E-02 | 7.38E-01 |
| SNRNP25 | mRNA | 0.76 | 2.20E-02 | 7.38E-01 |
| RNASEH2C | mRNA | 0.76 | 9.95E-03 | 7.38E-01 |
| EXO1 | mRNA | 0.76 | 2.86E-02 | 7.38E-01 |
| SYNM | mRNA | 0.76 | 6.81E-02 | 7.38E-01 |
| SRRT | mRNA | 0.76 | 1.59E-02 | 7.38E-01 |
| SPHK1 | mRNA | 0.76 | 2.68E-02 | 7.38E-01 |
| FEN1 | mRNA | 0.76 | 8.52E-03 | 7.38E-01 |
| TOE1 | mRNA | 0.77 | 3.39E-02 | 7.38E-01 |
| ZNF485 | mRNA | 0.77 | 3.74E-02 | 7.38E-01 |
| ZNF511 | mRNA | 0.77 | 7.31E-02 | 7.38E-01 |
| NME1 | mRNA | 0.77 | 5.32E-03 | 7.38E-01 |
| ZNF124 | mRNA | 0.77 | 8.58E-02 | 7.41E-01 |
| WDR76 | mRNA | 0.77 | 6.01E-02 | 7.38E-01 |
| NOP56 | mRNA | 0.77 | 2.17E-02 | 7.38E-01 |
| ASRGL1 | mRNA | 0.77 | 3.77E-02 | 7.38E-01 |
| DDX39A | mRNA | 0.77 | 1.63E-02 | 7.38E-01 |
| GPATCH4 | mRNA | 0.77 | 5.02E-02 | 7.38E-01 |
| IFI35 | mRNA | 0.77 | 5.12E-02 | 7.38E-01 |
| HSD11B2 | mRNA | 0.77 | 8.21E-02 | 7.38E-01 |
| PRPF31 | mRNA | 0.77 | 4.59E-03 | 7.38E-01 |
| SFXN5 | mRNA | 0.78 | 1.52E-02 | 7.38E-01 |
| BOLA2 | mRNA | 0.78 | 3.97E-03 | 7.38E-01 |
| BOLA2B | mRNA | 0.78 | 3.97E-03 | 7.38E-01 |
| MCM7 | mRNA | 0.78 | 6.04E-03 | 7.38E-01 |
| MTHFD1 | mRNA | 0.78 | 4.66E-03 | 7.38E-01 |
| HYPK | mRNA | 0.78 | 7.24E-03 | 7.38E-01 |
| ALYREF | mRNA | 0.78 | 9.74E-03 | 7.38E-01 |
| CLCN5 | mRNA | 0.78 | 3.15E-02 | 7.38E-01 |
| CCNE2 | mRNA | 0.79 | 3.39E-02 | 7.38E-01 |
| NETO2 | mRNA | 0.79 | 6.12E-02 | 7.38E-01 |
| HOXC8 | mRNA | 0.79 | 6.34E-02 | 7.38E-01 |
| MCM2 | mRNA | 0.79 | 3.09E-03 | 7.38E-01 |
| TUBA8 | mRNA | 0.80 | 7.02E-02 | 7.38E-01 |
| WDR4 | mRNA | 0.80 | 1.46E-02 | 7.38E-01 |
| CLSPN | mRNA | 0.80 | 1.87E-02 | 7.38E-01 |
| GPX2 | mRNA | 0.80 | 8.45E-02 | 7.38E-01 |
| HCN3 | mRNA | 0.80 | 3.09E-02 | 7.38E-01 |
| MPHOSPH6 | mRNA | 0.80 | 7.55E-02 | 7.38E-01 |
| TRMT61A | mRNA | 0.80 | 4.33E-02 | 7.38E-01 |
| PPP1R11 | mRNA | 0.81 | 4.97E-03 | 7.38E-01 |
| GMPPB | mRNA | 0.81 | 2.14E-02 | 7.38E-01 |
| ABCA12 | mRNA | 0.82 | 6.84E-03 | 7.38E-01 |
| COMTD1 | mRNA | 0.82 | 7.29E-03 | 7.38E-01 |
| FUT10 | mRNA | 0.82 | 4.38E-02 | 7.38E-01 |
| YY2 | mRNA | 0.82 | 9.38E-02 | 7.42E-01 |
| FNTB | mRNA | 0.82 | 6.45E-02 | 7.38E-01 |
| NUDT14 | mRNA | 0.82 | 6.85E-02 | 7.38E-01 |
| CDCA7 | mRNA | 0.82 | 5.25E-02 | 7.38E-01 |
| RAB19 | mRNA | 0.82 | 4.30E-02 | 7.38E-01 |
| TEDC1 | mRNA | 0.82 | 1.62E-02 | 7.38E-01 |
| C4BPB | mRNA | 0.83 | 7.02E-02 | 7.38E-01 |
| TIMM8B | mRNA | 0.83 | 1.67E-02 | 7.38E-01 |
| CTRC | mRNA | 0.83 | 7.53E-02 | 7.38E-01 |
| CDC25A | mRNA | 0.83 | 1.88E-02 | 7.38E-01 |
| HAS3 | mRNA | 0.83 | 3.45E-02 | 7.38E-01 |
| JAM3 | mRNA | 0.84 | 9.06E-02 | 7.42E-01 |
| RIMKLA | mRNA | 0.84 | 2.07E-02 | 7.38E-01 |
| ICOSLG | mRNA | 0.84 | 4.64E-02 | 7.38E-01 |
| ANXA9 | mRNA | 0.84 | 5.74E-02 | 7.38E-01 |
| UCP3 | mRNA | 0.85 | 8.46E-02 | 7.38E-01 |
| MCM10 | mRNA | 0.85 | 1.92E-02 | 7.38E-01 |
| RRM2 | mRNA | 0.85 | 2.42E-02 | 7.38E-01 |
| ADORA2B | mRNA | 0.85 | 7.62E-03 | 7.38E-01 |
| EEF1AKMT4 | mRNA | 0.86 | 7.93E-03 | 7.38E-01 |
| CENPM | mRNA | 0.86 | 8.56E-03 | 7.38E-01 |
| TNFRSF18 | mRNA | 0.86 | 2.04E-02 | 7.38E-01 |
| ANO1 | mRNA | 0.86 | 7.12E-03 | 7.38E-01 |
| SAPCD2 | mRNA | 0.87 | 1.53E-03 | 7.38E-01 |
| CLDND2 | mRNA | 0.87 | 4.18E-02 | 7.38E-01 |
| WNT3A | mRNA | 0.87 | 4.14E-02 | 7.38E-01 |
| HTRA4 | mRNA | 0.87 | 6.56E-02 | 7.38E-01 |
| MOG | mRNA | 0.87 | 8.36E-02 | 7.38E-01 |
| PUSL1 | mRNA | 0.87 | 3.92E-03 | 7.38E-01 |
| SMIM4 | mRNA | 0.88 | 1.92E-02 | 7.38E-01 |
| DNER | mRNA | 0.88 | 3.94E-02 | 7.38E-01 |
| SKIDA1 | mRNA | 0.88 | 5.47E-02 | 7.38E-01 |
| NHLRC1 | mRNA | 0.88 | 6.21E-02 | 7.38E-01 |
| LARGE1 | mRNA | 0.88 | 9.27E-02 | 7.42E-01 |
| MED22 | mRNA | 0.88 | 1.58E-02 | 7.38E-01 |
| BCAN | mRNA | 0.88 | 3.81E-02 | 7.38E-01 |
| PDSS1 | mRNA | 0.88 | 1.15E-02 | 7.38E-01 |
| NOB1 | mRNA | 0.89 | 2.79E-03 | 7.38E-01 |
| MREG | mRNA | 0.89 | 6.59E-03 | 7.38E-01 |
| CLN6 | mRNA | 0.89 | 2.97E-02 | 7.38E-01 |
| PAFAH2 | mRNA | 0.89 | 9.92E-03 | 7.38E-01 |
| NMB | mRNA | 0.89 | 6.81E-03 | 7.38E-01 |
| NPTX1 | mRNA | 0.91 | 7.29E-02 | 7.38E-01 |
| KCNK15 | mRNA | 0.91 | 8.08E-02 | 7.38E-01 |
| TCTA | mRNA | 0.91 | 6.25E-02 | 7.38E-01 |
| ZFP69B | mRNA | 0.91 | 6.26E-02 | 7.38E-01 |
| DNAH3 | mRNA | 0.92 | 5.55E-02 | 7.38E-01 |
| CREB3L2 | mRNA | 0.92 | 3.44E-03 | 7.38E-01 |
| CDH5 | mRNA | 0.92 | 4.01E-03 | 7.38E-01 |
| PTPRH | mRNA | 0.92 | 1.98E-02 | 7.38E-01 |
| MRM1 | mRNA | 0.92 | 2.94E-02 | 7.38E-01 |
| CENPV | mRNA | 0.92 | 2.14E-02 | 7.38E-01 |
| NT5DC2 | mRNA | 0.92 | 5.28E-03 | 7.38E-01 |
| ZWINT | mRNA | 0.93 | 3.89E-03 | 7.38E-01 |
| ISG15 | mRNA | 0.93 | 8.83E-02 | 7.42E-01 |
| ATAD3C | mRNA | 0.93 | 3.59E-02 | 7.38E-01 |
| ZNF578 | mRNA | 0.93 | 4.32E-02 | 7.38E-01 |
| B9D2 | mRNA | 0.94 | 7.89E-02 | 7.38E-01 |
| RHD | mRNA | 0.94 | 9.13E-02 | 7.42E-01 |
| MAP1LC3B2 | mRNA | 0.94 | 9.07E-02 | 7.42E-01 |
| MEFV | mRNA | 0.94 | 5.22E-02 | 7.38E-01 |
| MTERF1 | mRNA | 0.94 | 1.31E-02 | 7.38E-01 |
| NRGN | mRNA | 0.94 | 3.49E-02 | 7.38E-01 |
| MNS1 | mRNA | 0.94 | 2.27E-02 | 7.38E-01 |
| ATAD3B | mRNA | 0.95 | 5.15E-03 | 7.38E-01 |
| CBWD6 | mRNA | 0.95 | 4.81E-02 | 7.38E-01 |
| FJX1 | mRNA | 0.95 | 7.00E-03 | 7.38E-01 |
| PHOSPHO2 | mRNA | 0.96 | 8.58E-02 | 7.41E-01 |
| MDP1 | mRNA | 0.96 | 5.89E-02 | 7.38E-01 |
| POLE2 | mRNA | 0.96 | 2.19E-02 | 7.38E-01 |
| FAM151B | mRNA | 0.97 | 6.62E-02 | 7.38E-01 |
| ABCA9 | mRNA | 0.97 | 6.60E-02 | 7.38E-01 |
| GHRL | mRNA | 0.97 | 8.80E-02 | 7.42E-01 |
| FOSL1 | mRNA | 0.98 | 3.16E-02 | 7.38E-01 |
| TYW1B | mRNA | 0.98 | 4.38E-02 | 7.38E-01 |
| MRO | mRNA | 0.98 | 9.92E-02 | 7.42E-01 |
| GDF9 | mRNA | 0.99 | 9.86E-02 | 7.42E-01 |
| MIOX | mRNA | 0.99 | 3.94E-02 | 7.38E-01 |
| DNLZ | mRNA | 0.99 | 7.55E-02 | 7.38E-01 |
| MCM5 | mRNA | 0.99 | 9.46E-04 | 7.38E-01 |
| PTGES3L | mRNA | 1.00 | 7.81E-02 | 7.38E-01 |
| NPFF | mRNA | 1.00 | 9.33E-02 | 7.42E-01 |
| B3GAT1 | mRNA | 1.00 | 7.05E-02 | 7.38E-01 |
| UNC5B | mRNA | 1.00 | 4.21E-02 | 7.38E-01 |
| CAPN3 | mRNA | 1.00 | 8.58E-02 | 7.41E-01 |
| CARD9 | mRNA | 1.00 | 9.24E-02 | 7.42E-01 |
| UHRF1 | mRNA | 1.01 | 9.21E-03 | 7.38E-01 |
| CCDC189 | mRNA | 1.02 | 4.65E-02 | 7.38E-01 |
| SMIM27 | mRNA | 1.02 | 9.21E-02 | 7.42E-01 |
| MTHFS | mRNA | 1.03 | 5.67E-03 | 7.38E-01 |
| APOM | mRNA | 1.03 | 2.26E-02 | 7.38E-01 |
| CDC45 | mRNA | 1.03 | 3.88E-03 | 7.38E-01 |
| TRPC4 | mRNA | 1.04 | 6.69E-02 | 7.38E-01 |
| CYP8B1 | mRNA | 1.04 | 4.47E-02 | 7.38E-01 |
| ACOT1 | mRNA | 1.04 | 4.81E-02 | 7.38E-01 |
| SIGLEC11 | mRNA | 1.04 | 9.37E-02 | 7.42E-01 |
| KCNG3 | mRNA | 1.04 | 7.98E-02 | 7.38E-01 |
| TUBB8 | mRNA | 1.05 | 9.91E-02 | 7.42E-01 |
| RSPH1 | mRNA | 1.05 | 2.11E-02 | 7.38E-01 |
| ALOXE3 | mRNA | 1.05 | 1.70E-02 | 7.38E-01 |
| SHE | mRNA | 1.06 | 9.99E-02 | 7.42E-01 |
| PDF | mRNA | 1.06 | 7.29E-02 | 7.38E-01 |
| MALL | mRNA | 1.06 | 1.27E-02 | 7.38E-01 |
| CRLF2 | mRNA | 1.06 | 4.93E-02 | 7.38E-01 |
| FAM111B | mRNA | 1.07 | 1.42E-03 | 7.38E-01 |
| LGALS9B | mRNA | 1.07 | 9.27E-02 | 7.42E-01 |
| PGBD2 | mRNA | 1.07 | 9.36E-02 | 7.42E-01 |
| FGFBP3 | mRNA | 1.07 | 7.95E-02 | 7.38E-01 |
| NEURL3 | mRNA | 1.08 | 8.58E-03 | 7.38E-01 |
| ZG16 | mRNA | 1.08 | 7.38E-02 | 7.38E-01 |
| POC1B-GALNT4 | mRNA | 1.08 | 2.54E-02 | 7.38E-01 |
| GINS2 | mRNA | 1.08 | 8.71E-03 | 7.38E-01 |
| RIBC2 | mRNA | 1.08 | 5.08E-03 | 7.38E-01 |
| GP2 | mRNA | 1.08 | 2.65E-02 | 7.38E-01 |
| SURF2 | mRNA | 1.09 | 6.70E-02 | 7.38E-01 |
| CCL26 | mRNA | 1.10 | 6.28E-02 | 7.38E-01 |
| ALDH1A1 | mRNA | 1.10 | 6.99E-02 | 7.38E-01 |
| DACT2 | mRNA | 1.10 | 9.14E-02 | 7.42E-01 |
| NPIPB8 | mRNA | 1.11 | 2.85E-02 | 7.38E-01 |
| PPIF | mRNA | 1.11 | 4.09E-04 | 7.38E-01 |
| TFF1 | mRNA | 1.11 | 5.18E-02 | 7.38E-01 |
| FOSB | mRNA | 1.12 | 3.54E-02 | 7.38E-01 |
| SLC46A2 | mRNA | 1.12 | 9.05E-02 | 7.42E-01 |
| RPE65 | mRNA | 1.13 | 5.99E-02 | 7.38E-01 |
| B4GALNT2 | mRNA | 1.13 | 4.55E-02 | 7.38E-01 |
| RHCE | mRNA | 1.14 | 8.92E-02 | 7.42E-01 |
| CATSPER2 | mRNA | 1.14 | 4.03E-02 | 7.38E-01 |
| CXCL8 | mRNA | 1.15 | 1.56E-02 | 7.38E-01 |
| BTBD11 | mRNA | 1.15 | 1.67E-02 | 7.38E-01 |
| KYAT1 | mRNA | 1.16 | 3.38E-02 | 7.38E-01 |
| CCDC121 | mRNA | 1.16 | 5.46E-02 | 7.38E-01 |
| NKX1-2 | mRNA | 1.16 | 7.59E-03 | 7.38E-01 |
| PCDHA7 | mRNA | 1.17 | 8.81E-02 | 7.42E-01 |
| CYSRT1 | mRNA | 1.17 | 2.77E-02 | 7.38E-01 |
| MAMSTR | mRNA | 1.17 | 3.34E-02 | 7.38E-01 |
| CACNA1D | mRNA | 1.17 | 5.19E-02 | 7.38E-01 |
| ENHO | mRNA | 1.18 | 7.79E-02 | 7.38E-01 |
| PRR22 | mRNA | 1.20 | 8.02E-02 | 7.38E-01 |
| PSMC3IP | mRNA | 1.20 | 7.69E-03 | 7.38E-01 |
| SH3GL2 | mRNA | 1.20 | 9.61E-02 | 7.42E-01 |
| SUPT20HL2 | mRNA | 1.20 | 9.61E-02 | 7.42E-01 |
| FGFR2 | mRNA | 1.21 | 2.83E-02 | 7.38E-01 |
| ACOX2 | mRNA | 1.21 | 6.15E-02 | 7.38E-01 |
| NT5C1B-RDH14 | mRNA | 1.21 | 9.59E-02 | 7.42E-01 |
| AKR7L | mRNA | 1.21 | 5.84E-02 | 7.38E-01 |
| EFCAB3 | mRNA | 1.22 | 6.89E-02 | 7.38E-01 |
| C19orf67 | mRNA | 1.22 | 9.68E-02 | 7.42E-01 |
| ANKRD35 | mRNA | 1.22 | 9.89E-02 | 7.42E-01 |
| MAGEA2B | mRNA | 1.23 | 6.04E-02 | 7.38E-01 |
| HSPA2 | mRNA | 1.23 | 6.17E-02 | 7.38E-01 |
| SLC16A11 | mRNA | 1.23 | 8.66E-02 | 7.42E-01 |
| KCNH6 | mRNA | 1.24 | 8.17E-02 | 7.38E-01 |
| GJB1 | mRNA | 1.24 | 8.12E-02 | 7.38E-01 |
| SPDYE16 | mRNA | 1.25 | 1.97E-02 | 7.38E-01 |
| DGKG | mRNA | 1.25 | 1.39E-02 | 7.38E-01 |
| GLOD5 | mRNA | 1.26 | 7.38E-02 | 7.38E-01 |
| FOXP3 | mRNA | 1.26 | 9.90E-02 | 7.42E-01 |
| LVRN | mRNA | 1.26 | 9.90E-02 | 7.42E-01 |
| CA5A | mRNA | 1.27 | 6.94E-02 | 7.38E-01 |
| SLC22A18AS | mRNA | 1.29 | 2.56E-02 | 7.38E-01 |
| IL1RN | mRNA | 1.29 | 4.71E-02 | 7.38E-01 |
| ZNF888 | mRNA | 1.30 | 3.69E-02 | 7.38E-01 |
| CALML5 | mRNA | 1.31 | 2.94E-02 | 7.38E-01 |
| CLEC4E | mRNA | 1.31 | 4.02E-02 | 7.38E-01 |
| AKR7A3 | mRNA | 1.32 | 7.29E-02 | 7.38E-01 |
| CDH7 | mRNA | 1.32 | 8.86E-02 | 7.42E-01 |
| ABCA6 | mRNA | 1.32 | 9.95E-02 | 7.42E-01 |
| FAM110B | mRNA | 1.32 | 8.45E-02 | 7.38E-01 |
| SLC1A1 | mRNA | 1.33 | 3.87E-02 | 7.38E-01 |
| TACR1 | mRNA | 1.34 | 8.70E-02 | 7.42E-01 |
| EFCAB5 | mRNA | 1.34 | 7.85E-02 | 7.38E-01 |
| KCND3 | mRNA | 1.34 | 7.31E-02 | 7.38E-01 |
| STEAP1 | mRNA | 1.34 | 4.87E-03 | 7.38E-01 |
| MTRNR2L5 | mRNA | 1.35 | 7.12E-02 | 7.38E-01 |
| C4BPA | mRNA | 1.35 | 9.87E-02 | 7.42E-01 |
| FAT3 | mRNA | 1.35 | 2.92E-02 | 7.38E-01 |
| SLC28A2 | mRNA | 1.35 | 9.86E-02 | 7.42E-01 |
| HGD | mRNA | 1.37 | 9.96E-02 | 7.42E-01 |
| GPR75 | mRNA | 1.39 | 8.97E-02 | 7.42E-01 |
| BMP2 | mRNA | 1.41 | 3.04E-02 | 7.38E-01 |
| IL1A | mRNA | 1.41 | 7.86E-02 | 7.38E-01 |
| ZNF705E | mRNA | 1.41 | 6.78E-02 | 7.38E-01 |
| DUSP9 | mRNA | 1.42 | 4.51E-02 | 7.38E-01 |
| LRRC26 | mRNA | 1.43 | 5.17E-03 | 7.38E-01 |
| EIF3CL | mRNA | 1.43 | 6.64E-03 | 7.38E-01 |
| OXCT2 | mRNA | 1.44 | 3.66E-02 | 7.38E-01 |
| MPZ | mRNA | 1.44 | 5.53E-02 | 7.38E-01 |
| CLEC18B | mRNA | 1.45 | 8.30E-02 | 7.38E-01 |
| HIGD1B | mRNA | 1.46 | 7.47E-02 | 7.38E-01 |
| RIMBP3 | mRNA | 1.48 | 2.24E-02 | 7.38E-01 |
| KMO | mRNA | 1.49 | 1.76E-02 | 7.38E-01 |
| ASAH2 | mRNA | 1.49 | 1.32E-02 | 7.38E-01 |
| CATSPERB | mRNA | 1.50 | 6.22E-02 | 7.38E-01 |
| HCN4 | mRNA | 1.51 | 7.34E-02 | 7.38E-01 |
| SHISAL1 | mRNA | 1.52 | 2.18E-02 | 7.38E-01 |
| CHAC2 | mRNA | 1.53 | 5.68E-02 | 7.38E-01 |
| SPRR1B | mRNA | 1.53 | 2.27E-02 | 7.38E-01 |
| UBE2L5 | mRNA | 1.53 | 4.65E-02 | 7.38E-01 |
| RASL10B | mRNA | 1.55 | 9.04E-02 | 7.42E-01 |
| PATL2 | mRNA | 1.55 | 9.18E-02 | 7.42E-01 |
| TCHH | mRNA | 1.55 | 1.72E-02 | 7.38E-01 |
| LRRC31 | mRNA | 1.56 | 4.03E-02 | 7.38E-01 |
| CBFA2T3 | mRNA | 1.58 | 3.49E-03 | 7.38E-01 |
| DCX | mRNA | 1.58 | 7.84E-02 | 7.38E-01 |
| SYT6 | mRNA | 1.58 | 3.69E-02 | 7.38E-01 |
| POPDC2 | mRNA | 1.63 | 5.28E-02 | 7.38E-01 |
| PLXNA4 | mRNA | 1.64 | 4.43E-02 | 7.38E-01 |
| IL17C | mRNA | 1.65 | 5.23E-02 | 7.38E-01 |
| NR1I3 | mRNA | 1.66 | 4.47E-02 | 7.38E-01 |
| FZD8 | mRNA | 1.68 | 5.03E-02 | 7.38E-01 |
| IGFBP7 | mRNA | 1.69 | 4.54E-02 | 7.38E-01 |
| TMED6 | mRNA | 1.69 | 3.08E-02 | 7.38E-01 |
| CLDN20 | mRNA | 1.70 | 7.92E-02 | 7.38E-01 |
| SERPINB10 | mRNA | 1.71 | 7.63E-02 | 7.38E-01 |
| SSTR5 | mRNA | 1.71 | 9.26E-03 | 7.38E-01 |
| CHST13 | mRNA | 1.71 | 4.46E-02 | 7.38E-01 |
| CYP26A1 | mRNA | 1.71 | 4.39E-02 | 7.38E-01 |
| SNTN | mRNA | 1.73 | 8.28E-02 | 7.38E-01 |
| MYOT | mRNA | 1.73 | 1.73E-02 | 7.38E-01 |
| NECAB2 | mRNA | 1.73 | 8.88E-02 | 7.42E-01 |
| CXCL2 | mRNA | 1.74 | 5.62E-03 | 7.38E-01 |
| C11orf96 | mRNA | 1.75 | 8.38E-02 | 7.38E-01 |
| CXCL11 | mRNA | 1.75 | 8.38E-02 | 7.38E-01 |
| DPYS | mRNA | 1.75 | 8.38E-02 | 7.38E-01 |
| GADL1 | mRNA | 1.75 | 8.38E-02 | 7.38E-01 |
| KCNK9 | mRNA | 1.75 | 8.38E-02 | 7.38E-01 |
| NCR3 | mRNA | 1.75 | 8.38E-02 | 7.38E-01 |
| SLC24A3 | mRNA | 1.75 | 8.38E-02 | 7.38E-01 |
| OR10A3 | mRNA | 1.75 | 6.85E-02 | 7.38E-01 |
| PCDHB7 | mRNA | 1.76 | 6.71E-02 | 7.38E-01 |
| BPIFA2 | mRNA | 1.77 | 7.97E-02 | 7.38E-01 |
| CYP2C18 | mRNA | 1.77 | 6.77E-02 | 7.38E-01 |
| DBH | mRNA | 1.77 | 6.77E-02 | 7.38E-01 |
| FBLL1 | mRNA | 1.77 | 6.77E-02 | 7.38E-01 |
| GNAT1 | mRNA | 1.77 | 6.77E-02 | 7.38E-01 |
| GPC3 | mRNA | 1.77 | 6.77E-02 | 7.38E-01 |
| RPEL1 | mRNA | 1.77 | 6.77E-02 | 7.38E-01 |
| SLC25A2 | mRNA | 1.77 | 6.77E-02 | 7.38E-01 |
| TPSD1 | mRNA | 1.77 | 6.77E-02 | 7.38E-01 |
| CST4 | mRNA | 1.77 | 6.21E-02 | 7.38E-01 |
| PMCH | mRNA | 1.78 | 4.00E-02 | 7.38E-01 |
| ANXA10 | mRNA | 1.79 | 2.62E-02 | 7.38E-01 |
| ADGB | mRNA | 1.80 | 8.82E-02 | 7.42E-01 |
| PCYT1B | mRNA | 1.81 | 2.54E-02 | 7.38E-01 |
| ACRV1 | mRNA | 1.81 | 4.88E-02 | 7.38E-01 |
| CATSPERZ | mRNA | 1.81 | 9.50E-02 | 7.42E-01 |
| IFNW1 | mRNA | 1.81 | 7.98E-02 | 7.38E-01 |
| LRCH2 | mRNA | 1.81 | 7.98E-02 | 7.38E-01 |
| OR2A2 | mRNA | 1.81 | 7.98E-02 | 7.38E-01 |
| CYP2D6 | mRNA | 1.82 | 1.17E-02 | 7.38E-01 |
| SERPINA3 | mRNA | 1.82 | 2.13E-02 | 7.38E-01 |
| CCL20 | mRNA | 1.83 | 2.61E-02 | 7.38E-01 |
| LRRC71 | mRNA | 1.84 | 9.90E-03 | 7.38E-01 |
| BICC1 | mRNA | 1.84 | 4.60E-02 | 7.38E-01 |
| ZNF443 | mRNA | 1.88 | 1.34E-02 | 7.38E-01 |
| LPAR3 | mRNA | 1.91 | 3.35E-02 | 7.38E-01 |
| ASB12 | mRNA | 1.94 | 7.58E-02 | 7.38E-01 |
| C11orf91 | mRNA | 1.94 | 7.58E-02 | 7.38E-01 |
| PAQR9 | mRNA | 1.94 | 7.58E-02 | 7.38E-01 |
| FNDC5 | mRNA | 1.95 | 2.74E-02 | 7.38E-01 |
| KIF1A | mRNA | 1.95 | 7.45E-02 | 7.38E-01 |
| SERPIND1 | mRNA | 1.96 | 4.56E-02 | 7.38E-01 |
| GRIA3 | mRNA | 1.97 | 3.88E-02 | 7.38E-01 |
| BOLL | mRNA | 1.97 | 6.90E-02 | 7.38E-01 |
| COL20A1 | mRNA | 1.97 | 6.90E-02 | 7.38E-01 |
| TRIM61 | mRNA | 1.97 | 6.90E-02 | 7.38E-01 |
| RHEX | mRNA | 1.97 | 8.30E-02 | 7.38E-01 |
| DPP6 | mRNA | 1.98 | 6.78E-02 | 7.38E-01 |
| KIAA2012 | mRNA | 1.98 | 6.78E-02 | 7.38E-01 |
| KIRREL3 | mRNA | 1.98 | 1.55E-02 | 7.38E-01 |
| WNK3 | mRNA | 1.99 | 2.51E-02 | 7.38E-01 |
| KNG1 | mRNA | 2.00 | 3.61E-02 | 7.38E-01 |
| SETSIP | mRNA | 2.01 | 7.38E-02 | 7.38E-01 |
| TAS2R14 | mRNA | 2.01 | 7.38E-02 | 7.38E-01 |
| CTAGE4 | mRNA | 2.05 | 3.79E-02 | 7.38E-01 |
| PRSS55 | mRNA | 2.05 | 7.30E-02 | 7.38E-01 |
| CASQ2 | mRNA | 2.05 | 1.82E-02 | 7.38E-01 |
| JPH4 | mRNA | 2.05 | 9.64E-02 | 7.42E-01 |
| CSF2 | mRNA | 2.06 | 7.97E-02 | 7.38E-01 |
| FOXJ1 | mRNA | 2.08 | 1.15E-03 | 7.38E-01 |
| CCDC196 | mRNA | 2.09 | 6.73E-02 | 7.38E-01 |
| PHGR1 | mRNA | 2.10 | 3.75E-02 | 7.38E-01 |
| C2orf72 | mRNA | 2.10 | 9.46E-02 | 7.42E-01 |
| HECW1 | mRNA | 2.11 | 2.17E-02 | 7.38E-01 |
| CXCL5 | mRNA | 2.11 | 3.43E-02 | 7.38E-01 |
| MTNR1A | mRNA | 2.12 | 3.19E-02 | 7.38E-01 |
| FAM25G | mRNA | 2.13 | 9.87E-02 | 7.42E-01 |
| XKR6 | mRNA | 2.13 | 9.87E-02 | 7.42E-01 |
| CLEC3B | mRNA | 2.15 | 4.29E-02 | 7.38E-01 |
| NACA2 | mRNA | 2.15 | 4.29E-02 | 7.38E-01 |
| ADCY2 | mRNA | 2.15 | 6.42E-02 | 7.38E-01 |
| SPAG8 | mRNA | 2.16 | 5.32E-02 | 7.38E-01 |
| KLHDC7A | mRNA | 2.19 | 4.01E-03 | 7.38E-01 |
| C1orf167 | mRNA | 2.20 | 3.25E-02 | 7.38E-01 |
| PCDH19 | mRNA | 2.20 | 3.25E-02 | 7.38E-01 |
| TAL1 | mRNA | 2.20 | 3.25E-02 | 7.38E-01 |
| AMY1B | mRNA | 2.21 | 4.16E-02 | 7.38E-01 |
| BAAT | mRNA | 2.21 | 4.16E-02 | 7.38E-01 |
| NBPF6 | mRNA | 2.21 | 4.16E-02 | 7.38E-01 |
| SSX4 | mRNA | 2.21 | 4.16E-02 | 7.38E-01 |
| CTNND2 | mRNA | 2.23 | 1.64E-02 | 7.38E-01 |
| SPRR3 | mRNA | 2.23 | 5.32E-03 | 7.38E-01 |
| ZNF474 | mRNA | 2.24 | 6.34E-02 | 7.38E-01 |
| NKAIN1 | mRNA | 2.25 | 6.00E-02 | 7.38E-01 |
| LRRC19 | mRNA | 2.27 | 1.16E-02 | 7.38E-01 |
| SNRPN | mRNA | 2.27 | 8.43E-02 | 7.38E-01 |
| SNURF | mRNA | 2.27 | 8.43E-02 | 7.38E-01 |
| RNASE6 | mRNA | 2.27 | 5.50E-02 | 7.38E-01 |
| TRIM72 | mRNA | 2.27 | 5.50E-02 | 7.38E-01 |
| C10orf71 | mRNA | 2.28 | 5.64E-02 | 7.38E-01 |
| LEP | mRNA | 2.30 | 4.96E-02 | 7.38E-01 |
| ARRDC5 | mRNA | 2.39 | 1.10E-02 | 7.38E-01 |
| GPR182 | mRNA | 2.40 | 5.16E-02 | 7.38E-01 |
| S100Z | mRNA | 2.41 | 5.31E-02 | 7.38E-01 |
| CD247 | mRNA | 2.41 | 4.11E-02 | 7.38E-01 |
| DDX3Y | mRNA | 2.41 | 4.11E-02 | 7.38E-01 |
| MYOZ2 | mRNA | 2.41 | 4.11E-02 | 7.38E-01 |
| CXCL1 | mRNA | 2.41 | 1.21E-04 | 7.19E-01 |
| ADAMTS8 | mRNA | 2.45 | 4.12E-02 | 7.38E-01 |
| DRD1 | mRNA | 2.45 | 4.12E-02 | 7.38E-01 |
| LECT2 | mRNA | 2.45 | 4.12E-02 | 7.38E-01 |
| APELA | mRNA | 2.46 | 2.24E-02 | 7.38E-01 |
| UGT2B15 | mRNA | 2.46 | 2.24E-02 | 7.38E-01 |
| CYP4A11 | mRNA | 2.50 | 4.75E-02 | 7.38E-01 |
| TSGA13 | mRNA | 2.50 | 4.75E-02 | 7.38E-01 |
| UGT1A4 | mRNA | 2.51 | 6.88E-03 | 7.38E-01 |
| ATP1A4 | mRNA | 2.55 | 1.36E-02 | 7.38E-01 |
| SCHIP1 | mRNA | 2.56 | 8.89E-02 | 7.42E-01 |
| SRSF12 | mRNA | 2.58 | 5.66E-02 | 7.38E-01 |
| C2orf88 | mRNA | 2.58 | 3.63E-02 | 7.38E-01 |
| ABRA | mRNA | 2.59 | 1.63E-02 | 7.38E-01 |
| FGG | mRNA | 2.59 | 1.63E-02 | 7.38E-01 |
| OTOP1 | mRNA | 2.59 | 1.63E-02 | 7.38E-01 |
| ZACN | mRNA | 2.59 | 1.63E-02 | 7.38E-01 |
| CSH2 | mRNA | 2.60 | 4.39E-02 | 7.38E-01 |
| ZNF763 | mRNA | 2.63 | 3.15E-02 | 7.38E-01 |
| MYL2 | mRNA | 2.64 | 2.06E-02 | 7.38E-01 |
| VSTM2A | mRNA | 2.64 | 3.48E-02 | 7.38E-01 |
| KRT4 | mRNA | 2.66 | 2.01E-03 | 7.38E-01 |
| CORT | mRNA | 2.67 | 1.04E-02 | 7.38E-01 |
| PSG3 | mRNA | 2.69 | 1.41E-02 | 7.38E-01 |
| TRIM31 | mRNA | 2.77 | 1.00E-02 | 7.38E-01 |
| MS4A15 | mRNA | 2.85 | 1.19E-02 | 7.38E-01 |
| ACCSL | mRNA | 2.89 | 6.65E-02 | 7.38E-01 |
| GGTLC3 | mRNA | 3.09 | 7.42E-03 | 7.38E-01 |
| KLF15 | mRNA | 3.09 | 7.42E-03 | 7.38E-01 |
| RIIAD1 | mRNA | 3.15 | 7.20E-03 | 7.38E-01 |
| FER1L6 | mRNA | 3.24 | 6.46E-03 | 7.38E-01 |
| PCDHGA5 | mRNA | 3.28 | 1.41E-02 | 7.38E-01 |
| CEMP1 | mRNA | 3.30 | 9.43E-02 | 7.42E-01 |
| CSF3 | mRNA | 3.36 | 1.89E-02 | 7.38E-01 |
| GFRA3 | mRNA | 3.43 | 4.95E-03 | 7.38E-01 |
| ZNF177 | mRNA | 3.50 | 9.12E-02 | 7.42E-01 |
| TSSK2 | mRNA | 3.80 | 6.16E-03 | 7.38E-01 |
| PRODH | mRNA | 3.91 | 2.67E-03 | 7.38E-01 |
| TMEM140 | mRNA | 4.18 | 3.89E-02 | 7.38E-01 |
| SERF1A | mRNA | 4.22 | 2.24E-02 | 7.38E-01 |
| CDRT4 | mRNA | 4.58 | 4.28E-02 | 7.38E-01 |
