# Supplementary material for: TPX2-mediated autophagy maintains cancer stemness in LUAD: bioinformatic screening and functional validation
Source: Front Oncol. 2026 Jun 2;16:1724797. doi: 10.3389/fonc.2026.1724797 (PMC13269291; doi:10.3389/fonc.2026.1724797)
Supplement: Supplementary file 9 [file Table4.docx]

| **Supplementary Table 4. Primer Sequences for qRT-PCR and reverse transcription** | |
| --- | --- |
| Gene | Sequence (5’-3’) |
| TPX2 | Forward: ATGGAACTGGAGGGCTTTTTC |
|  | Reverse: TGTTGTCAACTGGTTTCAAAGGT |
| β-Actin | Forward: AGCGAGCATCCCCCAAAGTT |
|  | Reverse: GGGCACGAAGGCTCATCATT |
| SOX2 | Forward: GCCGAGTGGAAACTTTTGTCG |
|  | Reverse: GGCAGCGTGTACTTATCCTTCT |
| c-MYC | Forward: GGCTCCTGGCAAAAGGTCA |
|  | Reverse: CTGCGTAGTTGTGCTGATGT |
