# Supplementary material for: TPX2-mediated autophagy maintains cancer stemness in LUAD: bioinformatic screening and functional validation
Source: Front Oncol. 2026 Jun 2;16:1724797. doi: 10.3389/fonc.2026.1724797 (PMC13269291; doi:10.3389/fonc.2026.1724797)
Supplement: Supplementary file 10 [file Table5.docx]

| **Supplementary Table 5. Correlation between TPX2 Expression and Clinicopathological Characteristics of LUAD Patients** | | | | |
| --- | --- | --- | --- | --- |
|  | | TPX2 | | |
| Characteristics | Patients (n = 517) | Low (n = 258) | High (n = 259) | *p* Value |
| Gender |  |  |  | 0.05745 |
| Male | 239 | 108 | 131 |  |
| Female | 278 | 150 | 128 |  |
| Age (years) |  |  |  | 0.00247 |
| ≤ 65 | 259 | 114 | 145 |  |
| > 65 | 258 | 144 | 114 |  |
| TNM stage |  |  |  | 0.0362 |
| I-II | 407 | 212 | 195 |  |
| III-IV | 110 | 46 | 64 |  |
| Treatment type |  |  |  |  |
| Pharmaceutical Therapy | 263 | 120 | 143 | 0.047 |
| Radiation Therapy | 254 | 138 | 116 |  |
| The data were analyzed by chi-square test. | | | | |
